# High-entropy alloy nanopatterns by prescribed metallization of DNA origami templates

**DOI:** 10.1038/s41467-023-37333-y

**Published:** 2023-03-29

**Authors:** Mo Xie, Weina Fang, Zhibei Qu, Yang Hu, Yichi Zhang, Jie Chao, Jiye Shi, Lihua Wang, Lianhui Wang, Yang Tian, Chunhai Fan, Huajie Liu

**Affiliations:** 1 State Key Laboratory of Organic Electronics and Information Displays & Jiangsu Key Laboratory for Biosensors, Institute of Advanced Materials (IAM), Jiangsu National Synergetic Innovation Center for Advanced Materials (SICAM), Nanjing University of Posts and Telecommunications, 9 Wenyuan Road, 210023 Nanjing, China; 2 Division of Physical Biology, CAS Key Laboratory of Interfacial Physics and Technology, Shanghai Institute of Applied Physics, Chinese Academy of Sciences, 201800 Shanghai, China; 3 Shanghai Key Laboratory of Green Chemistry and Chemical Processes, Department of Chemistry, School of Chemistry and Molecular Engineering, East China Normal University, Dongchuan Road 500, 200241 Shanghai, China; 4 Key Laboratory of Advanced Civil Engineering Materials of Ministry of Education, School of Chemical Science and Engineering, Shanghai Research Institute for Intelligent Autonomous Systems, Tongji University, 200092 Shanghai, China; 5 Department of Medicinal Chemistry, School of Pharmacy, Fudan University, 826 Zhangheng Road, 201203 Shanghai, China; 6 School of Chemistry and Chemical Engineering, Frontiers Science Center for Transformative Molecules and National Center for Translational Medicine, Shanghai Jiao Tong University, 200240 Shanghai, China

**Keywords:** Organizing materials with DNA, Self-assembly

## Abstract

High-entropy multimetallic nanopatterns with controlled morphology, composition and uniformity hold great potential for developing nanoelectronics, nanophotonics and catalysis. Nevertheless, the lack of general methods for patterning multiple metals poses a limit. Here, we develop a DNA origami-based metallization reaction system to prescribe multimetallic nanopatterns with peroxidase-like activities. We find that strong coordination between metal elements and DNA bases enables the accumulation of metal ions on protruding clustered DNA (pcDNA) that are prescribed on DNA origami. As a result of the condensation of pcDNA, these sites can serve as nucleation site for metal plating. We have synthesized multimetallic nanopatterns composed of up to five metal elements (Co, Pd, Pt, Ag and Ni), and obtained insights on elemental uniformity control at the nanoscale. This method provides an alternative pathway to construct a library of multimetallic nanopatterns.

## Abstract (web summary)

Morphology, composition, and uniformity of highly entropic nanoalloys are critical to their properties and applications. Here, the authors develop a DNA origami-based metallization reaction concept for the precise synthesis of multimetallic nanopatterns.

## Introduction

Morphology- and element-control of metallic nanomaterials are of paramount importance in tailoring their intrinsic properties. While morphology-control of single metal element-based nanomaterials has been studied extensively^1,2^, multimetallic nanomaterials composed of two or more metal elements offer new potential and flexibility in tuning synergistic interactions, such as interfacing effect, surface strain, electronic structure, charge transfer, and plasmonic properties^3–12^. Typically, the success of wet synthesis of multimetallic nanomaterials in bulk solution has enabled diverse applications spanning from nanoelectronics, and nanophotonics, to catalysis^13–16^. Very recently, the emergence of substrate-supported approaches including dip-pen nanolithography^17–19^ and carbothermal shock synthesis^20,21^ has further promoted the development of multimetallic nanoparticles consisting of more elements. Nevertheless, patterning multimetallic nanomaterials remains challenging, mainly due to the difficulty in tunning multiple metal–substrate and metal–metal interactions simultaneously at predefined positions, especially for patterns with more than three metal elements^22,23^. Only in combination with top-down techniques, multimetallic nanoparticles could be assembled in a confined space that still has geometrical restrictions from imprinting methodology^24^.

With its intrinsic feature of nano-addressability^25,26^, DNA nanostructures have been considered as superb platforms for freestyle and site-specific organization of molecules and nanoparticles with sub-10 nm resolution^27–29^. Moreover, thanks to the programmability of the DNA hybridization process, the positioning of DNA nanostructures can strictly follow prescribed programs to make simultaneous morphology- and element-control possible, from both physical and chemical aspects^30–34^. Depending on design and materials, DNA nanostructure-directed patterning has profound implications in the fields of nanodevices, nanorobotics, nanomedicine, and molecular computation^35–37^. Especially, metal-patterning aided with DNA is a long-term focus and is fundamental to electrical and optical properties^38–42^.

Recently, we achieved the constructions of metal arrays on DNA origami templates through either nano-addressable anchoring strategy^43^ or in situ metallization^44,45^. For the latter, based on coordination between metal cations and bases in protruding clustered DNA (pcDNA), we established a nano-addressable pcDNA condensation and intrinsic metallization patterning (DCIMP) principle on all-DNA origami templates. Like in a recent report on DNA condensation on nanointerfaces^46^, metal cations can site-specifically coordinate with DNA bases in pcDNA prescribed on DNA origami substrate (osDNA) to increase the local concentration of metal ions. The condensed pcDNA with metal ions in turn serves as a nucleation site for the subsequent metal-plating process. Owing to the precision of the DNA origami template, the method can in principle paint metal lines with down to ~10 nm linewidth. Furthermore, the site-specific hybridization programmability endows the potential for fabricating single- and double-layer printed circuit board-like nanopatterns at the nanoscale.

Here, we exploit the nonconical condensation and metallization of the DCIMP strategy to develop multimetallic nanopatterns with peroxidase-like activities. We find that the atomic distribution uniformity of multimetallic nanopatterns can be regulated by specific elements. We have also demonstrated that this approach can be used to fabricate quinary multimetallic nanopatterns consisting of five different elements.

## Results

### Design principle

**Fig. 1:**
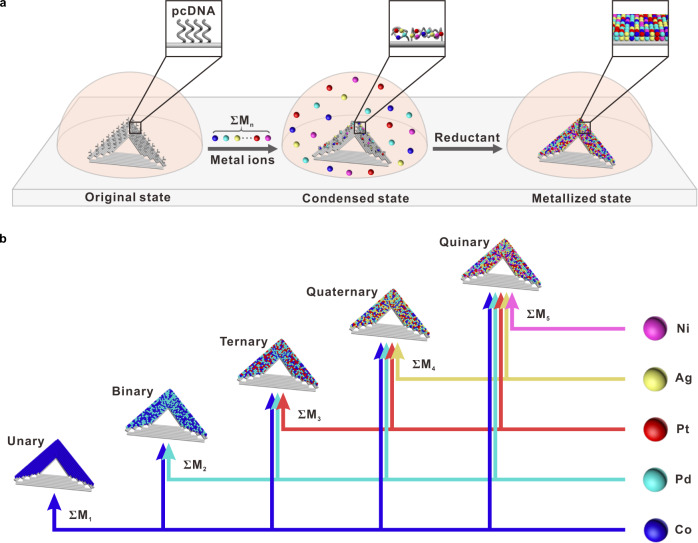
Templated synthesis of patterned multimetallic nanoparticles. **a** Scheme depicting three states of the synthetic process. Original state: DNA origami adsorbed on the substrate. Condensed state: various metal ion precursors selectively accumulated and coordination on pcDNA of DNA origami template. Metallized state: metal precursors reduced to multimetallic nanoparticles and site-specific metal-plating pattern generated on the template. Each colored ball represents a distinct metal ion or atom. **b** A five-element library of monometallic and multimetallic nanoparticles made via this method.

The design principle is shown in Fig. 1. A triangular DNA origami with side lengths of 120 nm, on which two edges were pre-anchored with pcDNAs and the other one edge was kept unchanged, was chosen as the template for DCIMP (referenced previous design^44^). According to our previous evidence about DCIMP, metal ions are expected to coordinate with pcDNAs that have abundant imine groups^44,45^. Since every single pcDNA located at its defined position has a high degree of flexibility and a lot of exposed imine groups, it supports multiple metal–imine interactions locally confined in the corresponding space. On the contrary, the stiffness of osDNA substrate and the embedded imines in base pairs hinder the free coordination between the metal atoms and osDNA and eliminate the localization of metal species. Given multiple coordination in a tiny apace, the pcDNAs undergo condensation with accumulated metal ions, leading to nano-addressable metallization on-origami substrate, with the help of a certain reductant. In the previous work, we have demonstrated that low-valence metal cations, including Cu^2+^ and Ag^+^, can accumulate, coordinate, and be metallized on pcDNAs by the DCIMP process to form metal nanoparticle patterns^44,45^. Here, we suggest that the DCIMP principle could be applied to both low-valence and high-valence metal cations, and furthermore, is not limited to cations but metal complex anions should work as well. We reason those imines in pcDNA are able to compete with weak ligands in a metal complex and form coordinating interactions with the metal atom.

To test our hypothesis that the DCIMP is effective for both metal cations and metal complex anions, we chose Ag, Co, Ni, Rh, Pt, and Pd as model elements. For Ag, Co, Ni, and Rh, their corresponding cations Ag^+^, Co^2+^, Ni^2+^, and Rh^3+^ were used. For Pt and Pd, we chose their common chloro complexes [PtCl_4_]^2−^ and [PdCl_4_]^2−^ with the consideration that Cl^−^ is a weak ligand for Pt^2+^ and Pd^2+^ and could be substituted by imine on pcDNA. Thus, multimetallic nanopatterning would be carried out with a solution of metal precursor mixture. As illustrated in Fig. 1a, following depositing DNA origami templates on the substrate (original state), DCIMP processes is initiated by adding the multimetallic plating solution to induce pcDNA-selective coordination (condensed state), which in turn benefits the highly localized metallization reaction occurring in the presence of reducing agents (metallized state). We note that due to the flexibility of two-dimensional origami structures, the deposition of origami templates before metal plating is important. In addition, the particles formed homogeneously in the solution are easy to be removed after on-origami metallization.

In our design, depending on the combination of metal precursors, unary, binary, ternary, quaternary, and even quinary alloys are expected to be prepared straightforwardly (Fig. 1b). The combination of metal species is denoted by Σ*M*_*n*_, where *M* is the metal element type and *n* is the number of metal species. For example, if one metal ion precursor (Co^2+^) is introduced, unary (Co, *n* = 1) metallized nanopatterns are expected to be constructed on the two pcDNA-decorated edges of the nano-triangle. Additionally, the combination of Co and Pd will lead to binary (CoPd, *n* = 2) nanopatterns with the same shape. In the same way ternary (CoPdPt, *n* = 3), quaternary (CoPdPtAg, *n* = 4), and quinary (CoPdPtAgNi, *n* = 5) nanopatterns would be obtained by adding Pt, Ag, and Ni precursors in the plating solution.

**Fig. 2:**
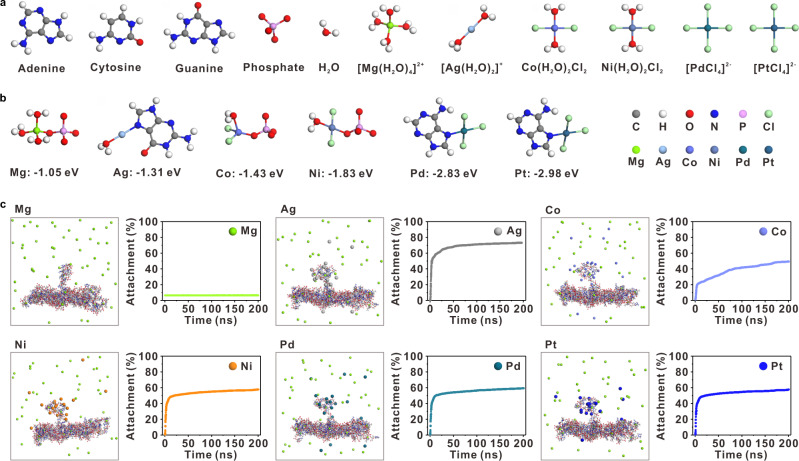
Theoretical simulations of interactions between metal ions and DNA molecules. **a** Geometric optimized structures from DFT calculations for molecules/ions (adenine, cytosine, guanine, phosphate, H_2_O, [Mg(H_2_O)_4_]^2+^, [Ag(H_2_O)_2_]^+^, Co(H_2_O)_2_Cl_2_, Ni(H_2_O)_2_Cl_2_, [PdCl_4_]^2−^, and [PtCl_4_]^2−^). **b** The most stable coordination complexes of different metal ions and bases/phosphate from first-principle calculated binding energy: magnesium-phosphate, silver-guanine, cobalt-phosphate, nickel-phosphate, palladium-adenine, and platinum-adenine. Each colored ball represents a distinct atom. **c** Full atomic MD simulations of typical types of metal ions attached to pcDNA strands prescribed on DNA origami templates, and variations in the attachment of metal ions to DNA within 200 ns. Source data are provided as a Source Data file.

Accordingly, the binding affinities of metal precursors to DNA were evaluated with density functional theory (DFT) simulations. As shown in Fig. 2a, imine-containing bases A, C, and G were employed as the models for the study. Considering the metal–nucleotide Columbic interactions, the phosphate group was also considered in the simulation. Besides, magnesium ion was taken into account, since the DNA origami template was prepared in a buffer containing Mg^2+^, which is always present in the metallization reaction system. The ion forms of selected metal elements Mg, Ag, Co, Ni, Pd, and Pt used for simulation were [Mg(H_2_O)_4_]^2+^, [Ag(H_2_O)_2_]^+^, Co(H_2_O)_2_Cl_2_, Ni(H_2_O)_2_Cl_2_, [PdCl_4_]^2−^, [PtCl_4_]^2−^, respectively. Based on first-principles calculations, the geometrically optimized structures of metal-base/phosphate complexes in the reaction system were obtained (Fig. 2b). For these metal species, three of them have high priorities to bind with bases, forming stable Ag-G (N7), Pd-A (N7), Pt-A (N7) complexes, while Co^2+^ and Ni^2+^ could bind with phosphate stably, and Mg^2+^ has the weakest binding capacity (Supplementary Table 1). We note that upon binding with bases, imine groups are preferred due to their higher basicities than amine groups^47^. Taken together, the order of binding capacities of metal ions to DNA follows the sequence of Mg < Ag < Co < Ni< Pd < Pt.

To reveal the localized accumulation of metal species around pcDNA on DNA templates, we performed full atomic molecular dynamics (MD) simulations on a DNA origami fragment model. The simulation accounted for bonded and non-bonded interactions between metal ions and templates. The results showed that Mg^2+^ did not accumulate around pcDNA or osDNA. In contrast, the other metal ions can be rapidly attached to pcDNA within a few nanoseconds, leading to condensation of pcDNA (Fig. 2c). Experimentally, from atomic force microscopy (AFM) images, some tiny bright spots that were slightly higher than the origami surface appeared at pcDNA positions, demonstrating the metal ions-induced pcDNA condensation after incubation (Supplementary Figs. 1 and 2).

### Unary metallization

**Fig. 3:**
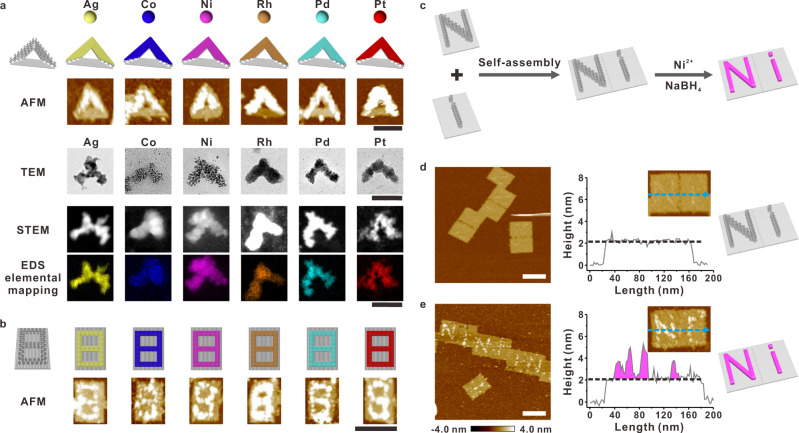
Synthesis of unary metal nanopatterns. **a** AFM images, TEM images, HAADF-STEM images, and EDS elemental mapping of each unary nanoparticle (Ag, Co, Ni, Rh, Pd, Pt) fabricated on two sides of a triangular DNA origami template. Each colored ball represents a distinct metal atom. **b** AFM images of the unary nanoparticle (Ag, Co, Ni, Rh, Pd, Pt) fabricated on a digital 8 of rectangle DNA origami template. **c** Schematic of assembly alphabet ‘Ni’ patterned rectangle origami dimer structure and Ni metallization process. AFM characterization of **d** before and **e** after Ni metallization of alphabet ‘Ni’ patterned DNA origami dimer and the corresponding height analysis. Scale bars: 100 nm. Color scales of all AFM images: from −4.0 to 4.0 nm. Source data are provided as a Source Data file.

Having obtained the binding capability sequence, we first investigated the nanopatterning of unary metallization following the DCIMP process with either a single low-valence metal cation (Ag^+^/Co^2+^/Ni^2+^), high-valence metal cation (Rh^3+^) or metal complex anion ([PdCl_4_]^2−^/[PtCl_4_]^2−^). As shown in Fig. 3a and Supplementary Fig. 3, under AFM measurements the expected V-shaped nanopatterns that are much higher than the origami surface could be clearly observed and are in accordance with the design. The third edge without pcDNA anchoring is unchanged after metal plating, confirming the site-specific feature of DCIMP that originated from the high condensation selectivity of pcDNA. Similar conclusions could be obtained from transmission electron microscopy (TEM) characterizations (Fig. 3a and Supplementary Figs. 4a–9a). Without any staining procedures, the metallized V-shaped patterns composed of small nanoparticles are demonstrated in these images, while the third edge with only osDNA is totally invisible. Furthermore, high-angle annular dark-field scanning TEM (HAADF-STEM) and energy-dispersive X-ray spectroscopy (EDX) were employed to correlate the morphology–composition relationship. By conducting both point scan and elemental mapping on the patterns, strong unary metal element signals were observed (Fig. 3a and Supplementary Figs. 4b–9b). Especially, for all six metal species the EDS mapping indicated that the metal element was distributed evenly throughout the whole V-shaped pattern, being in accordance with the corresponding morphological measurement. Moreover, we confirmed all six unary metal crystal structures are polycrystalline with selected area electron diffraction (SAED) and high-resolution TEM (HR-TEM) images (Supplementary Figs. 4–9).

Subsequently, the reaction process at different time points was monitored in order to understand the evolution of metal growth. As a representative, we explored the growth of Pd nanoparticles in the process of metallization. After a short reaction time of 0.5 min, tiny particles were observed in TEM imaging, showing incomplete V-shaped distribution. With the extension of reaction time to 2 min, these particles gradually grew, forming larger particles and a more complete V-shaped distribution. After 4 min of reaction, the particles grew into a more continuous structure. Based on the quantitative analysis of the metallized area, the size of the metallized area changed little in longer reactions, indicating that the reaction has been completed (Supplementary Fig. 10).

Next, we studied the diversity in patterning pcDNAs on different origami substrates and positions. A ‘digit-8’-shaped template with pcDNAs decorated on seven segments of narrow lines composing the ‘8’ shape on a tall rectangle origami was tested for plating metallic nanocircuits (Fig. 3b). After reduction, the height analysis shows that the metal nanoparticles are at least ~2 nm high from the DNA origami surface (Supplementary Fig. 11), slightly lower than the metal nanoparticles on the triangular DNA origami template (at least ~2.1 nm), which was related to the number of pcDNA^44^. We also systematically studied the influences of metal precursor concentration, reductant type, and concentration on nanopattern formation. It was found that there was an optimal metal precursor concentration range for each metal species, and insufficient or excess metal ions led to either discontinuous patterns or blurred images, respectively. The quantitative metallized area analysis showed the growth enhanced in the appropriate concentration range, while out of this range higher concentrations did not lead to greater enhancement (Supplementary Figs. 12–14). In addition, while metal ion concentration and reaction time were constant, more reducing agents would produce larger particles (Supplementary Fig. 15). With respect to different reducing agents, we found that when the stronger reductant was used, enhanced metallization was observed. Furthermore, while the same reducing agent acted on different metals, the more active metal was more likely to be metallized (Supplementary Figs. 16, 17).

Furthermore, regarding a single origami as a unit, integration of different metallic nanopatterns would be possible. To mimic nanocircuits integration, we explored the Ni metallization on two joint tall rectangle units with predefined pcDNAs patterns of alphabets ‘N’ and ‘i’, respectively (Fig. 3c). This dimer structure has a size of 100 nm × 150 nm and the original patterns were faintly visible under AFM characterizations (Fig. 3d). After Ni metallization, distinct ‘Ni’ patterns with an average height of 2 nm above origami substrate was observed, and the metallized lines have a width of ~10 nm calculated from the full width at half maximum (FWHM) (Fig. 3e). Similarly, other patterns such as ‘Ag’, ‘Co’, ‘Rh’, ‘Pt’ and ‘Pd’ could be fabricated from their corresponding metal elements (Supplementary Fig. 18).

### Precise multimetallic nanopatterns

**Fig. 4:**
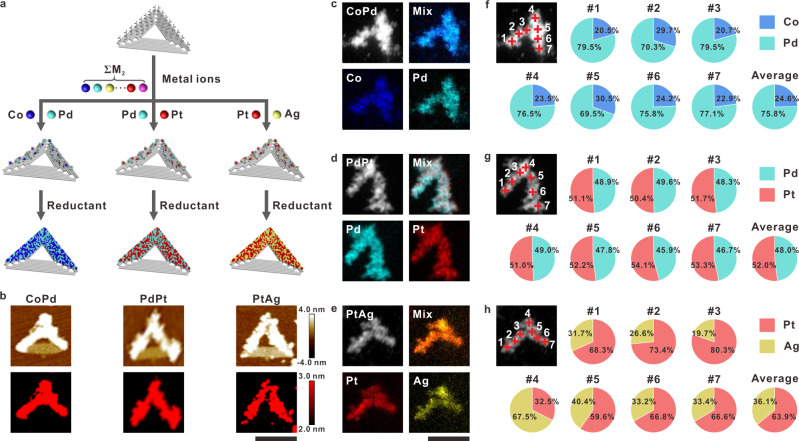
Synthesis of various binary nanoparticles. **a** Scheme depicting the process. Each colored ball represents a distinct metal ion or atom. **b** AFM images of the binary nanoparticle (CoPd, PdPt, PtAg) fabricated on two sides of a triangular DNA origami template. Second line shows tomography images of nanoparticles areas above the origami surface. Color scales of AFM images: from −4.0 to 4.0 nm. Color scales of cross-section images: from 2.0 to 3.0 nm. **c–e** HAADF-STEM images and EDS elemental mapping of each binary nanopattern on template: CoPd, PdPt, and PtAg. **f–h** Atomic percentage distributions for each element at different positions on a binary nanoparticle: CoPd (the average composition is Co (24.6 ± 4.0%), Pd (75.4 ± 4.0%)), PdPt (the average composition is Pd (48.0 ± 1.3%), Pt (52.0 ± 1.3%)), and PtAg (the average composition is Pt (63.9 ± 15.3%), Ag (36.1 ± 15.3%)). Scale bars: 100 nm. Source data are provided as a Source Data file.

Having established the generality of DCIMP for unary metallization, we next extended this strategy to binary systems. To show that different combinations could strongly influence the composition and elemental distribution in the final product, three typical cases of combinations—CoPd, PdPt, and PtAg—were demonstrated. Technically, two species of metal precursors were equally mixed together and dropped onto DNA origami templates. Afterward, a reduction step was carried out following the DCIMP process (Fig. 4a). The morphological and compositional characterizations executed by AFM and TEM confirmed the success in fabricating metallized nanopatterns, from which V-shaped patterns that are 2–9 nm higher than the pcDNA-free third edge of the triangle were observed (Fig. 4b and Supplementary Figs. 19–21). The elemental distributions in these three samples were determined by EDS mapping accompanying with HAADF-STEM measurement. Notably, the results showed that in all three samples, two distinct metal elements are evenly distributed in the patterns (Fig. 4c–e). We further performed point-by-point elemental analyses to substantiate the uniformity by examining elemental compositions at seven selected positions on each sample (Fig. 4f–h and Supplementary Figs. 19–21). With only one exception (the corner point of the PtAg sample), on all positions, the uniform binary nature was proved. It is interesting that while equimolar metal precursors were employed, the contents of varied metals in the as-prepared nanopatterns were different. In addition, all these three binary nanoparticles are polycrystalline and their crystalline structures vary from corresponding individual unary metals, as evidenced by SAED and diffraction pattern intensity profile analysis (Supplementary Figs. 19–21), which indicated the formation of binary alloys.

**Fig. 5:**
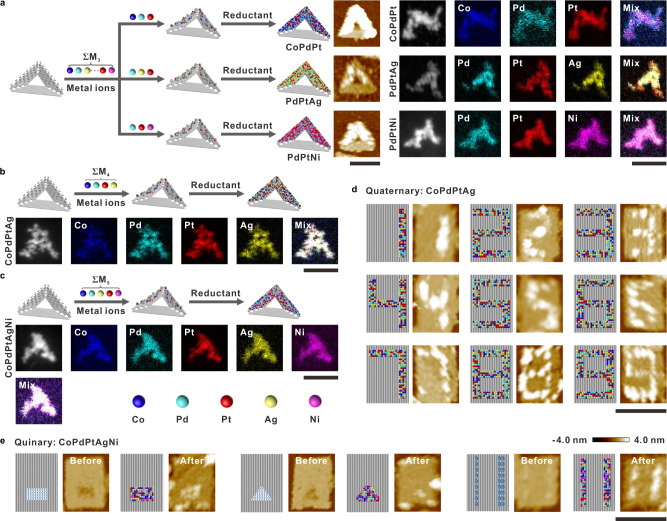
Synthesis of multimetallic nanopatterns. Characterization of nanoparticles on two sides of triangular DNA origami template: **a** various ternary nanoparticles: CoPdPt, PdPtAg, and PdPtNi. **b** Quaternary (CoPdPtAg) nanoparticle. **c** Quinary (CoPdPtAgNi) nanoparticle. To show the generality, **d**, digits from 1 to 9 were fabricated by quaternary (CoPdPtAg) nanoparticles, and **e** several typical shapes (two digging holes and different thicknesses of lines) were fabricated by quinary (CoPdPtAgNi) nanoparticles, as shown in corresponding AFM images. Scale bars: 100 nm. Color scales of AFM images: from −4.0 to 4.0 nm.

Next, the above success on binary systems enabled us to extend the same strategy to multimetallic patterning with three or more components (Fig. 5). Accordingly, a mixture of prescribed metal precursors was cast onto origami substrates with pcDNAs for the subsequent reduction. Based on the studied binary systems, one more metal species was combined. As examples of ternary systems, we explored three combinations of Co–Pd–Pt, Pd–Pt–Ag and Pd–Pt–Ni co-plating on the triangular origami. AFM imaging revealed the expected V-shaped patterns which have 2–5 nm height over the origami substrate (Fig. 5a and Supplementary Figs. 22–24). Together with TEM, EDS, and SAED characterizations, the co-existence of three metals throughout the whole pattern and formed crystalline structures different from individual components were confirmed.

A quaternary system was further constructed by combining Ag to CoPdPt ternary system. Considering that Ag is immiscible with Co and probably more elements in this set of combinations, it would help us elucidating the underlying in situ co-metallization mechanism. As shown in Fig. 5b, as far as similar V-shaped morphologies were observed with AFM and TEM (Supplementary Fig. 25), the employment of Ag caused a significant decrease in elemental uniformity in the patterns. Especially, the point-by-point composition examination indicated that inhomogeneous distribution occurred almost everywhere. The immiscible nature indicates that phase separation exists in this system induced by Ag, while in the former CoPdPt ternary system alloys with high entropy are believed to be the main product. Given metal miscibility decides the intrinsic structural property of nanopatterns, it might be possible to regulate the formation of alloys or phase-separated products with specific elements^17–19^. Besides, we demonstrated that the shape of quaternary nanopatterns could also be well controlled by changing the template. As an example, we designed dights ‘0’ to ‘9’ patterns by applying specific combinations of the seven segments of ‘digit-8’ template. The AFM images carried out on the quaternary CoPdPtAg sample indicated the success of the designs (Fig. 5d and Supplementary Fig. 26).

Finally, to challenge the fabrication of quinary nanopatterns, Ni was combined with these four elements via the DCIMP process. The growth of CoPdPtAgNi was studied at different time points (Supplementary Fig. 27). We found that its growth was similar to that of Pd, in which first tiny particles formed and then grew up to form a complete V-shaped structure. As expected, the contents of five metal elements are not even at the tested seven positions on the V-shaped metallized patterns (Fig. 5c and Supplementary Fig. 28). Due to the fact that Ni is also immiscible with Ag, this result is reasonable and further confirmed the hypothesis that metal–metal interactions play pivotal roles. Besides the V-shape with protruding pcDNAs over the surface of the DNA origami substrate, we also employed two concave templates with omitted staple strands in the rectangle origami for testing the substrate generality (Fig. 5e and Supplementary Fig. 29a, c). The omission of staple strands leads to the exposure of single-stranded scaffold DNA in the formed ‘hole’. Thus, the scaffold here is able to be condensed with metal precursors and works as pcDNA. AFM imaging proved the effectiveness of this substrate, from which the ‘holes’ were found to be filled with metal nanoparticles. This result is in accordance with the metallization on pcDNA extended on-origami surface (Fig. 5e and Supplementary Fig. 29b, d), demonstrating the success of the DCIMP process in creating multimetallic nanopatterns with compositional and morphological complexity. Furthermore, we designed a DNA origami template with single and double lines of pcDNA, which were metallized to form metal lines of different thicknesses (Fig. 5e and Supplementary Fig. 29e), demonstrating that the linewidth can be flexibly adjusted by the design of the template.

In total, it is interesting that the metal contents in the formed patterns roughly follow the order of Ag < Co < Pd < Pt <Ni, which is not consistent with the order of reduction capacity of metal precursors: Co < Ni < Pd < Pt < Ag (Supplementary Table 2). Instead, it correlates well with the binding capability sequence of Ag < Co < Ni < Pd < Pt, with the exception of Ni. These results indicate that the strong binding of metal precursors facilitates their accumulation around pcDNA and the subsequent metallization. As an exceptional case, the interactions between Ni species and pcDNA might be complicated. For example, DFT calculations on the Ni atom, the reduction product, revealed that it can bind strongly to both bases and phosphate (Supplementary Fig. 30).

### Peroxidase-like activities of nanoalloys

Fine metallic nanoparticles are often used as nanozymes with enzyme-like activities, which have received widespread attention due to the high stability, low cost, and comparable catalytic properties with natural enzymes^48^. The combination of two or more metals in a nanozyme structure presents additional properties due to synergistic effects^49,50^. In order to investigate the enzyme-like activities of multimetallic structures, the chromogenic substrate of 3,3′,5,5′-tetramethyl-benzidine (TMB) in the presence of H_2_O_2_ was used to evaluate the catalytic performance. Given oxidation of TMB happens in the presence of H_2_O_2_, a blue product (TMBox) with a characteristic absorption at 652 nm can be formed, otherwise negligible absorbance and color change can be found (Supplementary Fig. 31). Here we tested and compared the catalytic activities of various as-prepared products and found that only Pt among the unary metals exhibited obvious peroxidase-like activity, while the others showed weak or negligible catalytic properties (Supplementary Figs. 32, 33). In contrast, all tested multimetallic nanoproducts exhibited peroxidase-like activities with different intensities. Interestingly, we found that the introduction of Ag in the components led to a decrease in catalytic activity while the introduction of Ni led to enhanced catalytic performance, in accordance with their contributions to elemental uniformity in nanoalloys. These results demonstrated that alloys are better than unary metals in catalytic activity.

**Fig. 6:**
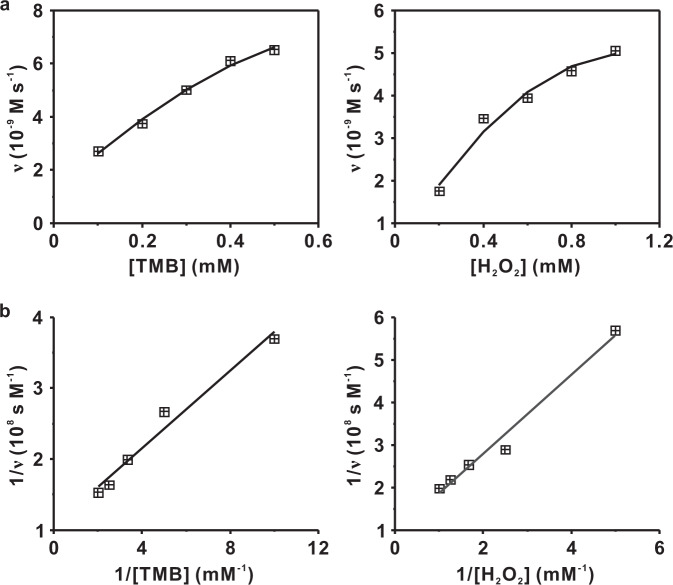
Peroxidase-like activities of multimetallic nanopatterns. **a** Michaelis–Menten fit of catalytic performance of CoPdPt. **b** Lineweaver–Burk plots for CoPdPt. Left panels in **a** and **b**: using constant 20 mM of H_2_O_2_ with different concentrations of TMB (0.1–0.5 mM). Right panels in **a** and **b**: using constant 1 mM of TMB with different concentrations of H_2_O_2_ (0.2–1.0 mM). Source data are provided as a Source Data file.

Among the multimetallic nanoalloys synthesized by the DCIMP process, CoPdPt had the highest catalytic activity. As a model material, we explored the steady-state kinetics by adjusting substrate concentration (Supplementary Fig. 34). As shown in Fig. 6a, b, the fitting curves on both TMB and H_2_O_2_ conformed to the Michaelis–Menten model. Accordingly, the *K*_M_ value was 0.25 mM for TMB and the *V*_max_ value was 0.94 × 10^−8^ M s^−1^ (*y* = 0.272*x* + 1.062, *R*^2^ = 0.973), while the *K*_M_ value was 0.98 mM for H_2_O_2_ and the *V*_max_ value was 1.06 × 10^−8^ M s^−1^ (*y* = 0.925*x* + 0.944, *R*^2^ = 0.981). The lower *K*_M_ value for H_2_O_2_ indicated that CoPdPt has a higher affinity to the substrate than that of horseradish peroxidase (HRP) (3.70 mM)^51^ (Table 1).

**Table 1:** Comparison of apparent kinetic parameters of CoPdPt nanopatterns and HRP enzyme

Enzyme	Substrate	*K*_M_ (mM)	*V*_max_ (10^-8^ M s^−1^)	Reference
CoPdPt	TMB	0.25	0.94	This work
CoPdPt	H_2_O_2_	0.98	1.06	This work
HRP	TMB	0.43	10.00	Ref. ^51^
HRP	H_2_O_2_	3.70	8.71	Ref. ^51^

## Discussion

In summary, we have achieved multimetallic patterning with DCIMP approach. Using DNA origami as a nano-addressable template, nanocircuit-mimic patterns composed of up to five metal elements could be fabricated by in situ metallization at prescribed positions. Importantly, besides low-valence metal cations, high-valence metal cations and metal complex anions were proved to be effective precursors. We also found that metal miscibility can determine the composition uniformity of nanopatterns. For example, with the presence of Ag, phase separation probably occurred. On the contrary, miscible multimetallic nanopatterns with uniform atomic distribution were observed. Thus, the results may give inspiration to developing methodologies for uniformity and phase separation control. We also note that the multimetallic nanopatterns all exhibited peroxidase-like activities. Especially, it is envisioned that by nano-addressable metallization, nanoalloys arrays with specific shapes and components could be prepared, providing implications for nanozymes and nano-factory for catalysis.

## Methods

### Materials and instruments

Single-stranded M13mp18 DNA (7249 nucleotides) was purchased from New England Biolabs. All other DNA strands were synthesized by Invitrogen (China) with PAGE purified (sequences listed in Supporting Information). Reagents were purchased from Sinopharm and Sigma-Aldrich and used without further purification. Metal ion precursors: Ag^+^ (AgNO_3_, ≥99.0%), [Ag(NH_3_)_2_]^+^ (prepared with AgNO_3_ and 25.0–28.0% ammonia in deionized water), Co^2+^ (CoCl_2_·6H_2_O, 98.0%), Ni^2+^ (NiCl_2_, 98.0%), Rh^3+^ (RhCl_3_, 98.0%), [PdCl_4_]^2−^ (K_2_PdCl_4_, 98.0%), [PtCl_4_]^2−^ (K_2_PtCl_4_, 98.0%), and reductants: glutaraldehyde (23.0–27.0%), phenol (>98.0%), glucose (≥99.0%), formaldehyde (37.0–40.0%), dimethylamine borane (97%), sodium borohydride (≥98.0%). TEM measurements were obtained by Tecnai G2 F20 S-TWIN equipped with an EDS analyzer (acceleration voltage: 200 kV). AFM images were carried out with a Bruker multimode VIII atomic force microscope (Veeco Inc., USA).

### Preparation of DNA origami template

Single-stranded M13mp18 DNA and all staple strands with a molar ratio of 1:10 were mixed in 1× TAE–Mg^2+^ buffer (40 mM Tris, 20 mM acetic acid, 2 mM EDTA, 12.5 mM magnesium acetate, pH 8.0). The mixture was annealed from 95 to 4 °C to form the DNA origami template. Excess staples were removed using 100 kDa Ultrafiltration Spin-Columns. For the assembly of the alphabet patterned dimer template, first, the concentrations of the purified monomers were quantified by UV absorbance measuring at 260 nm, then the two monomers were mixed stoichiometrically and 10 times excess of linker chains were added. The mixture was annealed from 45 to 20 °C to form the dimer structure.

### Metallization process

After the DNA origami template was adsorbed on the substrate, a one-step or two-step metallization process was performed. In the one-step process, the metal ion precursor and reducing agent were added separately and carefully onto the EP tube wall to avoid pre-mixing reactions, followed by the addition of 1× TA–Mg^2+^ buffer (40 mM Tris, 20 mM acetic acid, 12.5 mM magnesium acetate, pH 8.0) wash-in to mix and then transfer them (200 μL system with a final concentration of 4 mM metal ion and 20 mM reductants) to the substrate rapidly. In the two-step process, 100 μL of metal ions (in 1× TA–Mg^2+^ buffer) were added to the substrate first, followed by the addition of 100 μL reductants (in 1× TA–Mg^2+^ buffer), which was pipetted and mixed at the time of addition (200 μL system with a final concentration of 4 mM metal ion and 20 mM reductants), then localized metallization reaction occurred and generated metal nanoparticles on DNA origami template. After 10 min reaction at room temperature, wash the products with 200 μL 1× TA–Mg^2+^ buffer 5–6 times. For the synthesis of unary metal and multimetallic nanopatterns without Ag, both one-step and two-step processes were applicable, with no significant differences found in the results. While for multimetallic nanopatterns containing Ag, a one-step process was required to avoid the formation of AgCl. All multimetallic syntheses were performed using equimolar mixes of various metal precursors with a final concentration of 4 mM and a final concentration of 20 mM of reducing agent. Various reducing agents (glutaraldehyde, phenol, glucose, formaldehyde, dimethylamine borane, sodium borohydride) can be used for the reduction of metal ions, in which dimethylamine borane, sodium borohydride being the most versatile. In addition to the study on experimental conditions, other metal structures were synthesized under the above condition, and sodium borohydride was used for the synthesis of Co, Ag, Ni, Rh, and DMAB was used for the synthesis of Pd, Pt, and alloys, respectively.

### Sample preparation of metal nanoparticles for AFM measurement

The preparation method of the nanoparticles was the same as those mentioned in the “Metallization process” section. DNA origami was adsorbed on a mica surface for 2 min, then added reaction solutions to initiate DCIMP processes. After metallization, the sample was washed with 1× TA–Mg^2+^ buffer and imaged in ‘PeakForce QNM in Fluid’ in solution using ‘SCANASYST-FLUID+’ tips.

### Sample preparation of metal nanoparticles for TEM measurement

The carbon-coated copper grids (200 mesh, Beijing Zhongjingkeyi Technology Co., Ltd.) as substrate was cleaned by a Harrick Plasma PDC-32G cleaner for 30 s at a low RF level. The DNA origami was deposited onto a pretreated grid for 2 min. A piece of filter paper was used to remove the excess sample. After the metallization protocol mentioned in the metallization process section, the reaction solution was washed with 1× TA–Mg^2+^ buffer and dried at room temperature. The sample was analyzed using TEM, HAADF-STEM, HR-TEM, and SEAD technology. The data were analyzed using DigitalMicrograph, TIA, and PDF4-2009 software.

### DFT simulation

The Gaussian 09 program and GAUSSVIEW 5.0 visualization program were carried out for all ab initio calculations. The binding energy between the nucleic bases with metal ions/atoms is defined as

1 $${E}_{{{{{{\rm{abs}}}}}}}={E}_{{{{{{\rm{total}}}}}}}-{E}_{{{{{{\rm{metal\; ion}}}}}}/{{{{{\rm{atom}}}}}}}-{E}_{{{{{{\rm{base}}}}}}}$$

*E*_abs_ represent the absorption energy, and *E*_total_, *E*_metal ion/atom_, *E*_base_ represents the energy of the final complex, metal ion/atom, and the corresponding nucleic base, respectively.

### MD simulation

MD simulations were performed with the GROMACS package version 2019 beta^52^ with Amber03 forcefield for all-atom simulations^53^. The extra charges of DNA origami were balanced with Mg^2+^ and Cl^−^ ions and parameters for other metal ions were adapted from universal forcefield^54^. All simulations were performed with a time step of 10 fs and a set temperature of 300 K, with velocity-rescaling thermostats^55^, with a time constant for coupling of 1 ps. An isotropic pressure of 1 bar was maintained with the Berendsen barostat^56^, with a compressibility of 3 × 10^−4^ bar^−1^, and a relaxation time constant of 50 ps. Production runs with a duration of 200 ns for all atomic simulations was used.

### Peroxidase-like activity assay of metal nanoparticles

The peroxidase activity of metal nanoparticles was evaluated by catalytic oxidation of TMB in the presence of H_2_O_2_. 2 μL 2 nM DNA origami was adsorped on the surface of mica, and parallel samples of various metal nanoparticles were prepared by DCIMP process. 50 μL reaction system (Custom TMB Substrate, purchased from NEOGEN) was added to the mica substrate and incubated at room temperature for 2 min. Drawing the reaction system, measuring the UV–vis absorption spectrum, and observing color changes.

### Kinetics assay of CoPdPt nanozymes

The steady-state kinetics investigation of CoPdPt nanozymes by changing the concentration of TMB and H_2_O_2_. Constant 20 mM H_2_O_2_ with different concentrations of TMB (0.1–0.5 mM), while constant 1 mM TMB with different concentrations of H_2_O_2_ (0.2–1.0 mM). Calculation of apparent kinetic parameters (*K*_M_ and *V*_max_) according to Michaelis–Menten saturation curve. Michaelis–Menten equation:

2 $${{{{{\rm{\nu }}}}}}=\frac{{V}_{{{\max }}}[{{{{{\rm{S}}}}}}]}{{K}_{{{{{{\rm{M}}}}}}}+[{{{{{\rm{S}}}}}}]}$$

*ν* represent the reaction rate, *K*_M_ represents the Michaelis–Menten constant, *V*_max_ represents the maximum reaction velocity, [S] represents the concentration of substrate.

### Reporting summary

Further information on research design is available in the Nature Portfolio Reporting Summary linked to this article.

## Supplementary information

Supplementary Information

Reporting Summary

## Data Availability

Source data are provided with this paper. Any additional data is available from the authors upon request. Source data are provided with this paper.

## Source data

Source Data
