# Supplementary material for: High-entropy alloy nanopatterns by prescribed metallization of DNA origami templates
Source: Nat Commun. 2023 Mar 29;14:1745. doi: 10.1038/s41467-023-37333-y (PMC10060391; doi:10.1038/s41467-023-37333-y)
Supplement: Supplementary file 1 — Supplementary Information [file 41467_2023_37333_MOESM1_ESM.pdf]

Supplementary information for

High-entropy alloy nanopatterns by prescribed metallization of DNA origami templates

Mo Xie[1,2†], Weina Fang[3,4†], Zhibei Qu[5†], Yang Hu[1], Yichi Zhang[6], Jie Chao[1], Jiye Shi[2], Lihua Wang[2], Lianhui Wang[1], Yang Tian[3], Chunhai Fan[6*], Huajie Liu[4*]

[1]State Key Laboratory of Organic Electronics and Information Displays & Jiangsu Key Laboratory for Biosensors, Institute of Advanced Materials (IAM), Jiangsu National Synergetic Innovation Center for Advanced Materials (SICAM), Nanjing University of Posts and Telecommunications, 9 Wenyuan Road, Nanjing 210023, China.
[2]Division of Physical Biology, CAS Key Laboratory of Interfacial Physics and Technology, Shanghai Institute of Applied Physics, Chinese Academy of Sciences, Shanghai 201800, China.
[3]Shanghai Key Laboratory of Green Chemistry and Chemical Processes, Department of Chemistry, School of Chemistry and Molecular Engineering, East China Normal University, Dongchuan Road 500, Shanghai 200241, China.
[4]School of Chemical Science and Engineering, Shanghai Research Institute for Intelligent Autonomous Systems, Key Laboratory of Advanced Civil Engineering Materials of Ministry of Education, Tongji University, Shanghai 200092, China.
[5]Department of Medicinal Chemistry, School of Pharmacy, Fudan University, 826 Zhangheng Road, Shanghai 201203, China.
[6]School of Chemistry and Chemical Engineering, Frontiers Science Center for Transformative Molecules and National Center for Translational Medicine, Shanghai Jiao Tong University, Shanghai 200240, China.
†These authors contributed equally: Mo Xie, Weina Fang, Zhibei Qu.
*E-mail: fanchunhai@sjtu.edu.cn; liuhuajie@tongji.edu.cn

This file includes:
Supplementary Figures 1 to 52
Supplementary Table 1 to 2
Supplementary Notes: DNA sequences
Supplementary References

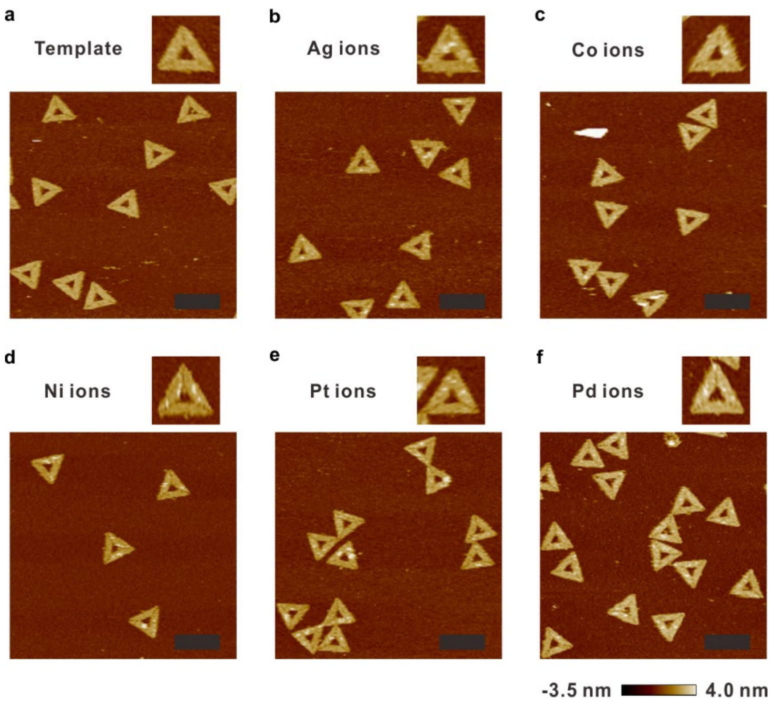

Supplementary Figure 1. Metal ions interact with pcDNA on triangular DNA origami template, causing condensation of pcDNA. AFM images of origami samples treated with different metal ions: (a) DNA origami template without treatment, (b) Ag ions, (c) Co ions, (d) Ni ions, (e) Pt ions, (f) Pd ions. Scale bars: 200 nm. Colour scales of all AFM images: from -3.5 nm to 4.0 nm.

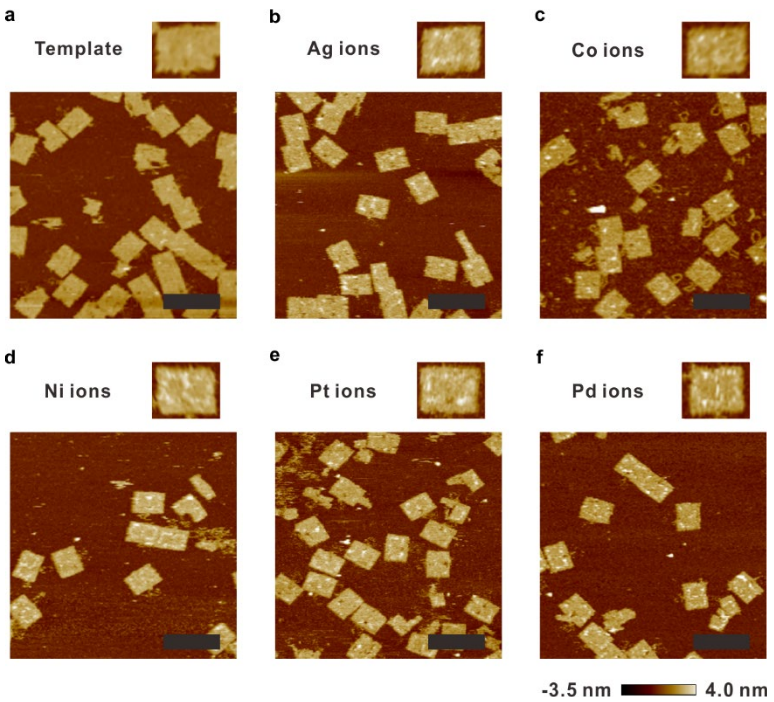

Supplementary Figure 2. Metal ions interact with pcDNA on digit 8 of rectangular DNA origami template, causing condensation of pcDNA. AFM images of origami samples treated with different metal ions: (a) DNA origami template without treatment, (b) Ag ions, (c) Co ions, (d) Ni ions, (e) Pt ions, (f) Pd ions. Scale bars: 200 nm. Colour scales of all AFM images: from -3.5 nm to 4.0 nm.

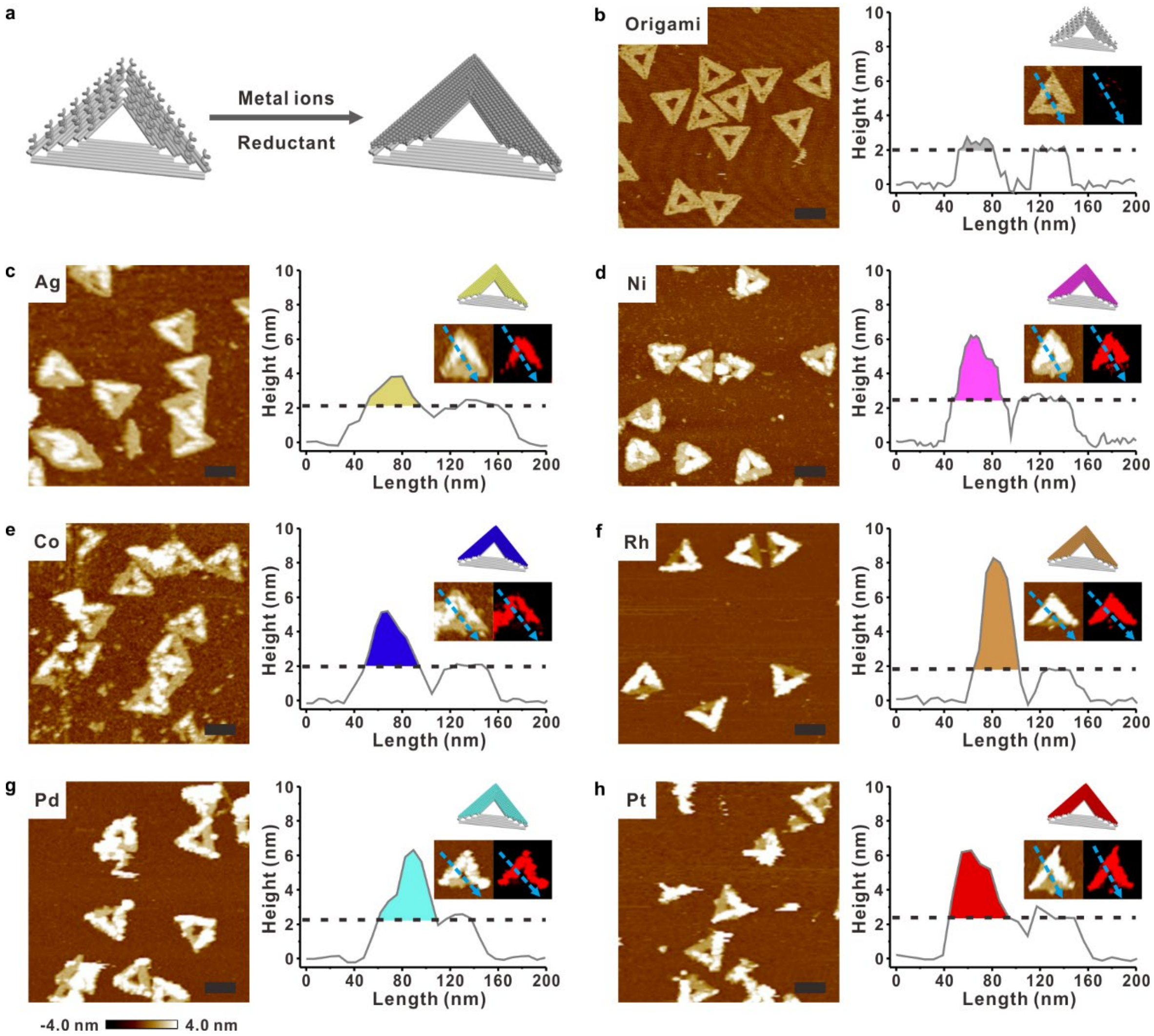

Supplementary Figure 3. AFM images of each unary metal nanoparticles on two sides of triangular DNA origami template and the corresponding height analysis. (a) Scheme depicting the template before and after metallization. (b) Triangular DNA origami structure with pcDNA strands (pcDNA average height: ~0.5 nm (above the origami surface, hereinafter are the same), average FWHM: ~33.1 nm). Each metallization unary nanopatterns: (c) Ag (Ag nanoparticles average height: ~2.1 nm, average FWHM: ~35.5 nm). (d) Ni (Ni nanoparticles average height: ~3.8 nm, average FWHM: ~35.6 nm). (e) Co (Co nanoparticles average height: ~2.1 nm, average FWHM: ~36.6 nm). (f) Rh (Rh nanoparticles average height: ~6.2 nm, average FWHM: ~31.0 nm). (g) Pd (Pd nanoparticles average height: ~4.1 nm, average FWHM: ~34.3 nm). (h)Pt (Pt nanoparticles average height: ~6.2 nm, average FWHM: ~39.1 nm). Scale bar: 100 nm. Colour scales of all AFM images: from -4.0 nm to 4.0 nm. Source data are provided as a Source Data file.

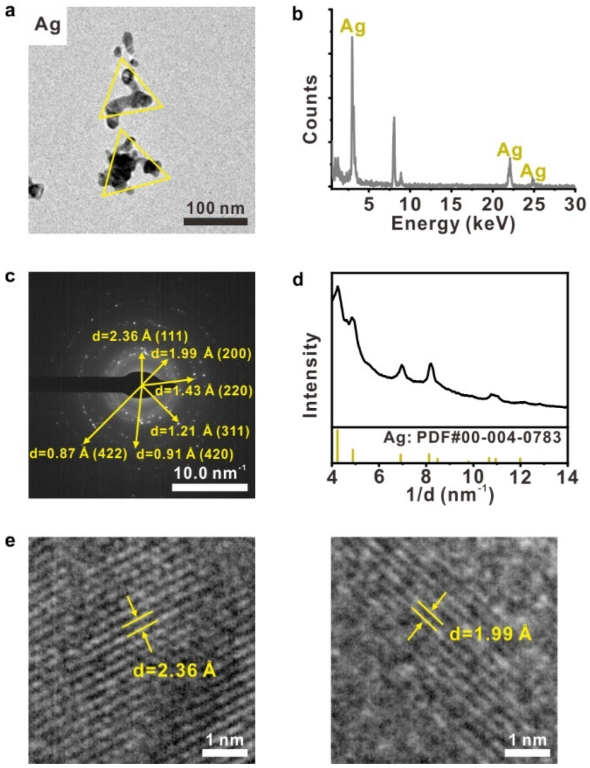

Supplementary Figure 4. TEM characterization of elements on two sides of triangular DNA origami template after DCIMP processes of silver unary system. (a) TEM image of V-shaped silver pattern. (b) EDX spectrum analysis. (c) Selected area electron diffraction (SAED) image presents ring diffraction pattern of a polycrystalline silver specimen. (d) Diffraction pattern intensity profile of silver specimen. (e) High-resolution TEM (HR-TEM) image of a region of Ag nanoparticle. Source data are provided as a Source Data file.

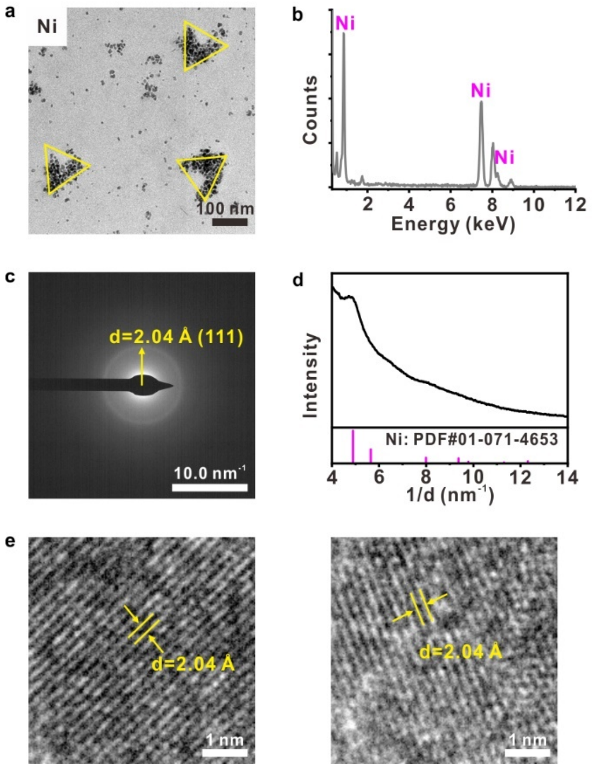

Supplementary Figure 5. TEM characterization of elements on two sides of triangular DNA origami template after DCIMP processes of nickel unary system. (a) TEM image of V-shaped nickel pattern. (b) EDX spectrum analysis. (c) SAED image presents ring diffraction pattern of a polycrystalline nickel specimen. (d) Diffraction pattern intensity profile of nickel specimen. (e) HR-TEM image of a region of Ni nanoparticle. Source data are provided as a Source Data file.

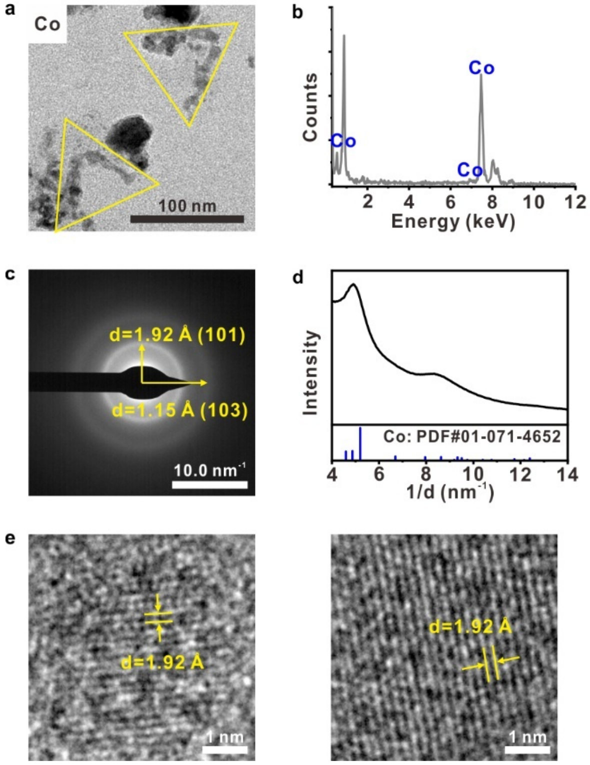

Supplementary Figure 6. TEM characterization of elements on two sides of triangular DNA origami template after DCIMP processes of cobalt unary system. (a) TEM image of V-shaped cobalt pattern. (b) EDX spectrum analysis. (c) SAED image presents ring diffraction pattern of a polycrystalline cobalt specimen. (d) Diffraction pattern intensity profile of cobalt specimen. (e) HR-TEM image of a region of Co nanoparticle. Source data are provided as a Source Data file.

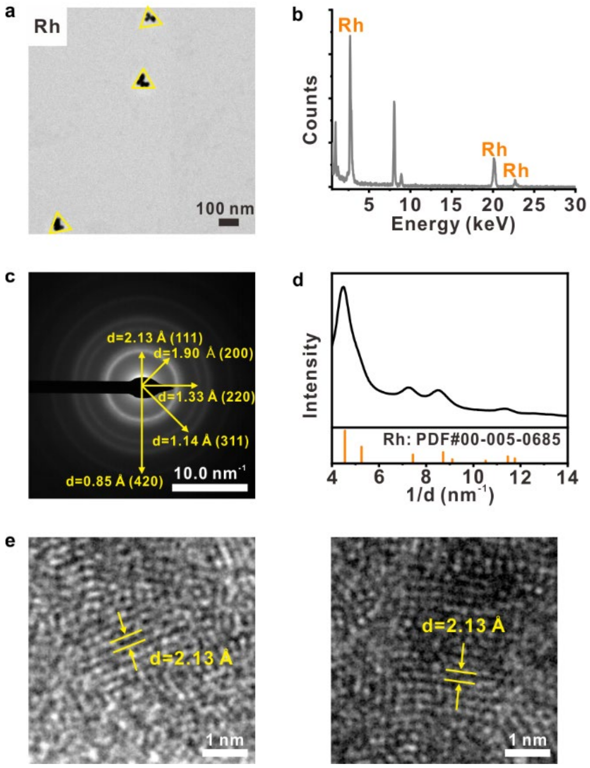

Supplementary Figure 7. TEM characterization of elements on two sides of triangular DNA origami template after DCIMP processes of rhodium unary system. (a) TEM image of V-shaped rhodium pattern. (b) EDX spectrum analysis. (c) SAED image presents ring diffraction pattern of a polycrystalline rhodium specimen. (d) Diffraction pattern intensity profile of rhodium specimen. (e) HR-TEM image of a region of Rh nanoparticle. Source data are provided as a Source Data file.

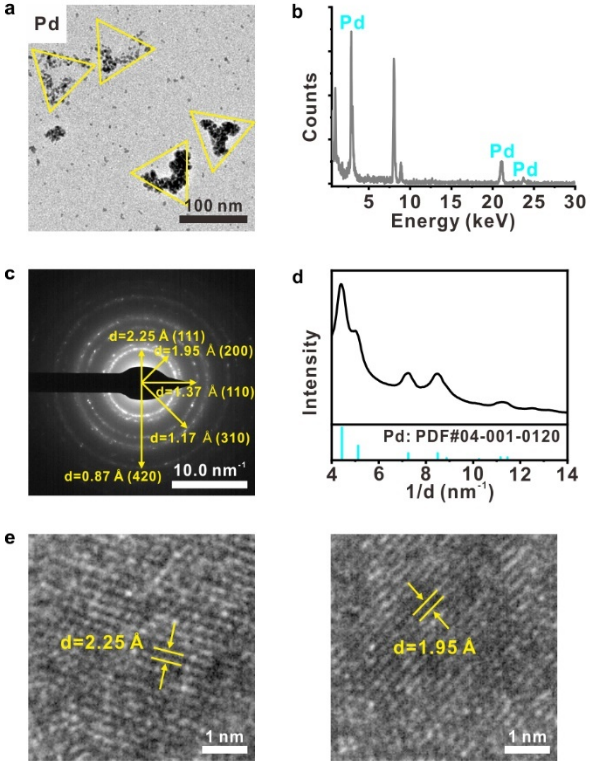

Supplementary Figure 8. TEM characterization of elements on two sides of triangular DNA origami template after DCIMP processes of palladium unary system. (a) TEM image of V-shaped palladium pattern. (b) EDX spectrum analysis. (c) SAED image presents ring diffraction pattern of a polycrystalline palladium specimen. (d) Diffraction pattern intensity profile of palladium specimen. (e) HR-TEM image of a region of Pd nanoparticle. Source data are provided as a Source Data file.

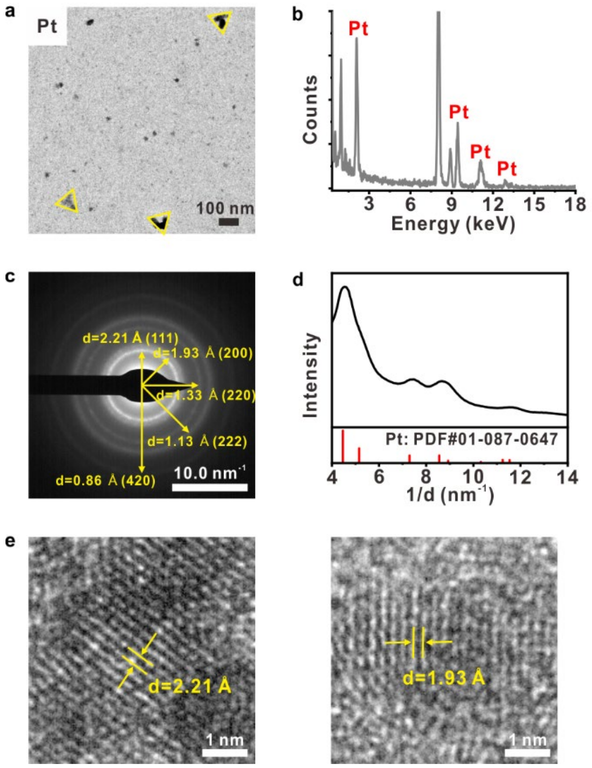

Supplementary Figure 9. TEM characterization of elements on two sides of triangular DNA origami template after DCIMP processes of platinum unary system. (a) TEM image of V-shaped platinum pattern. (b) EDX spectrum analysis. (c) SAED image presents ring diffraction pattern of a polycrystalline platinum specimen. (d) Diffraction pattern intensity profile of platinum specimen. (e) HR-TEM image of a region of Pt nanoparticle. Source data are provided as a Source Data file.

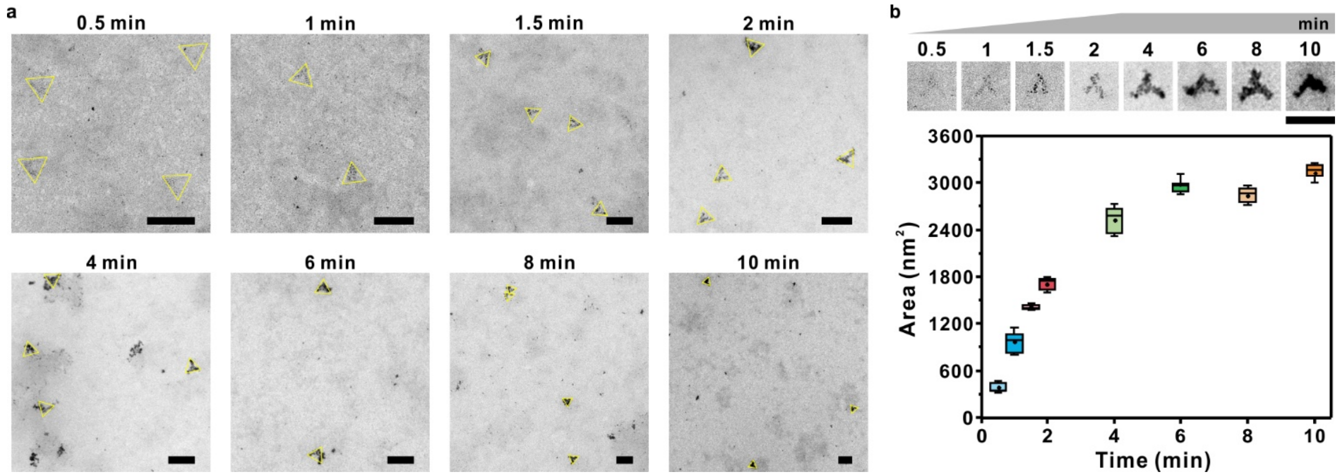

Supplementary Figure 10. Pd morphological evolutions on triangular DNA origami template at different reaction time points. (a) TEM image. (b) Representative structure and quantitative analysis of the Pd area at different reaction time points (N=5, error bars: SD). Scale bars: 200 nm. Source data are provided as a Source Data file.

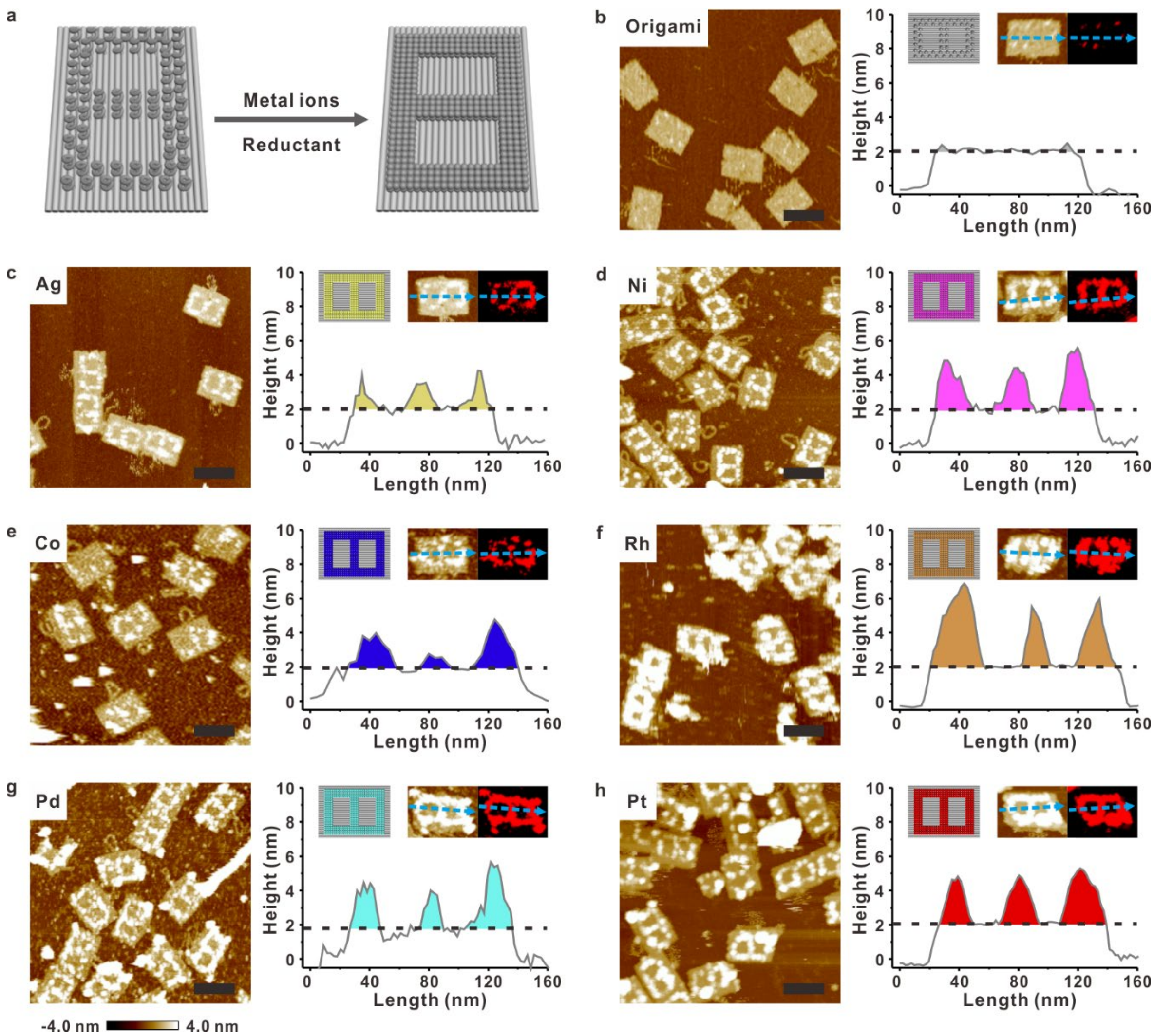

Supplementary Figure 11. AFM images of each unary metal nanoparticles on digit 8 of rectangular DNA origami template and the corresponding height analysis. (a) Scheme depicting the template before and after metallization. (b) Rectangular DNA origami structure with digit 8 parttened pcDNA strands (pcDNA average height: ~0.4 nm (above the origami surface, hereinafter are the same), average FWHM: ~4.6 nm). Each metallization unary nanopatterns: (c) Ag (Ag nanoparticles average height: ~2.0 nm, average FWHM: ~8.8 nm). (d) Ni (Ni nanoparticles average height: ~2.9 nm, average FWHM: ~14.2 nm). (e) Co (Co nanoparticles average height: ~2.0 nm, average FWHM: ~15.9 nm). (f) Rh (Rh nanoparticles average height: ~4.1 nm, average FWHM: ~16.6 nm). (g) Pd (Pd nanoparticles average height: ~2.9 nm, average FWHM: ~13.4 nm). (h)Pt (Pt nanoparticles average height: ~3.0 nm, average FWHM: ~16.3 nm). Scale bar: 100 nm. Colour scales of all AFM images: from -4.0 nm to 4.0 nm. Source data are provided as a Source Data file.

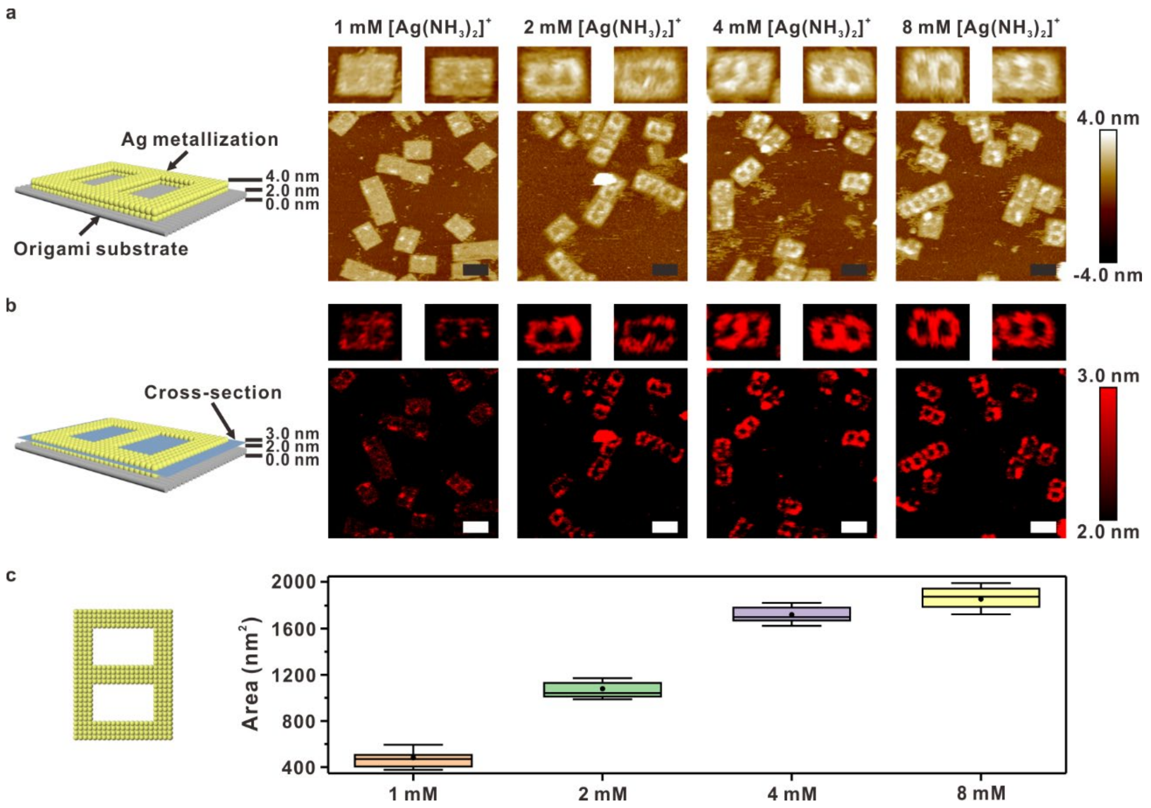

Supplementary Figure 12. Silver nanopatterns constructed on digit 8 templates with different concentrations of silver precursor ($[Ag(NH_3)_2]^+$). Reductant concentration: 20 mM Formaldehyde. Reaction time: 10 min. (a) AFM images of Ag metallization on origami substrate. (b) Tomography images of silver nanopatterns areas above the origami surface. Tomographic of cross-sectional areas at 2 nm to 3 nm height. (c) Quantitative analysis of metal area (N=5, error bars: SD). Scale bars: 100 nm. Source data are provided as a Source Data file.

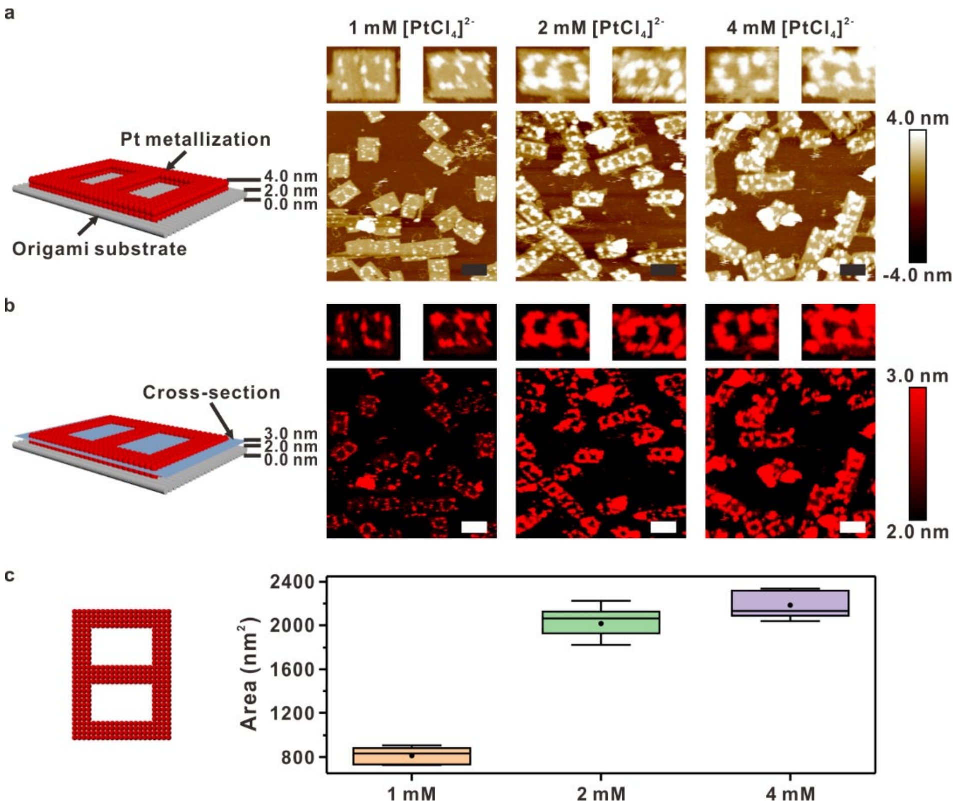

Supplementary Figure 13. Platinum nanopatterns constructed on digit 8 patterns with different concentrations of platinum precursor ($[PtCl_4]^{2-}$). Reductant concentration: 20 mM DMAB. Reaction time: 10 min. (a) AFM images of Pt metallization on origami substrate. (b) Tomography images of platinum nanopatterns areas above the origami surface. Tomographic of cross-sectional areas at 2 nm to 3 nm height. (c) Quantitative analysis of metal area (N=5, error bars: SD). Scale bars: 100 nm. Source data are provided as a Source Data file.

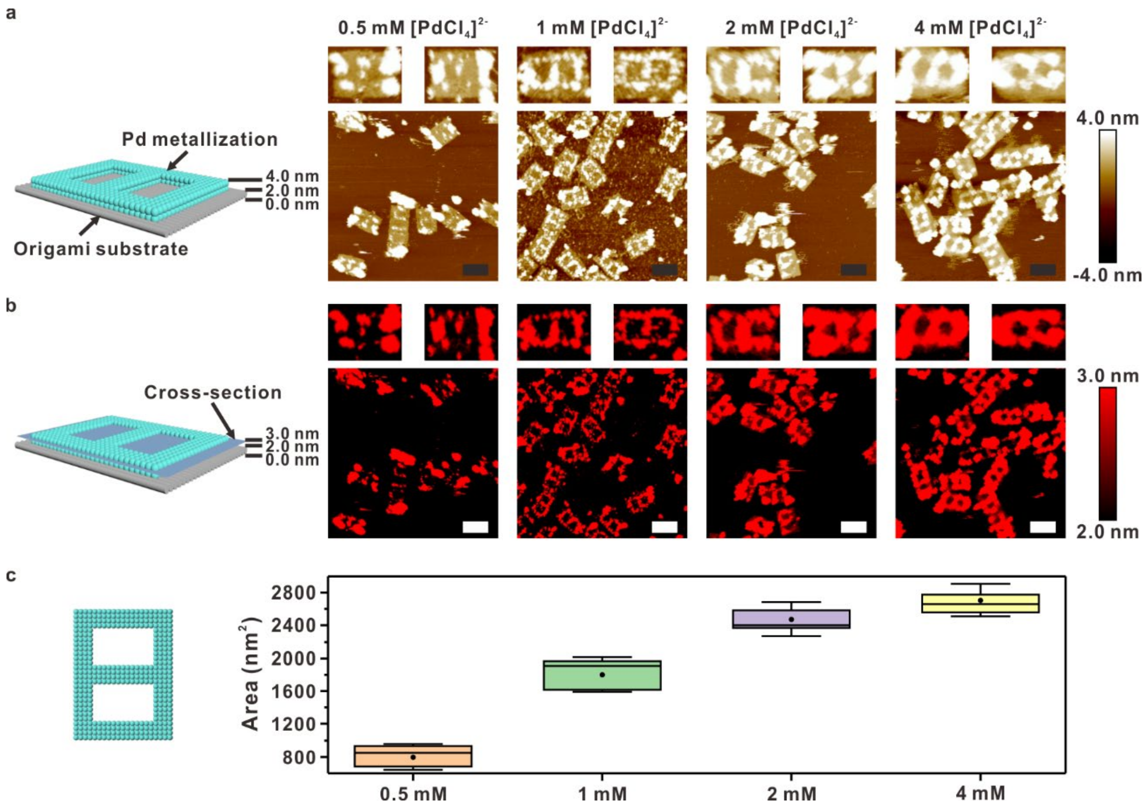

Supplementary Figure 14. Palladium nanopatterns constructed on digit 8 patterns with different concentrations of palladium precursor ($[PdCl_4]^{2-}$). Reductant concentration: 20 mM DMAB. Reaction time: 10 min. (a) AFM images of Pd metallization on origami substrate. (b) Tomography images of palladium nanopatterns areas above the origami surface. Tomographic of cross-sectional areas at 2 nm to 3 nm height. (c) Quantitative analysis of metal area (N=5, error bars: SD). Scale bars: 100 nm. Source data are provided as a Source Data file.

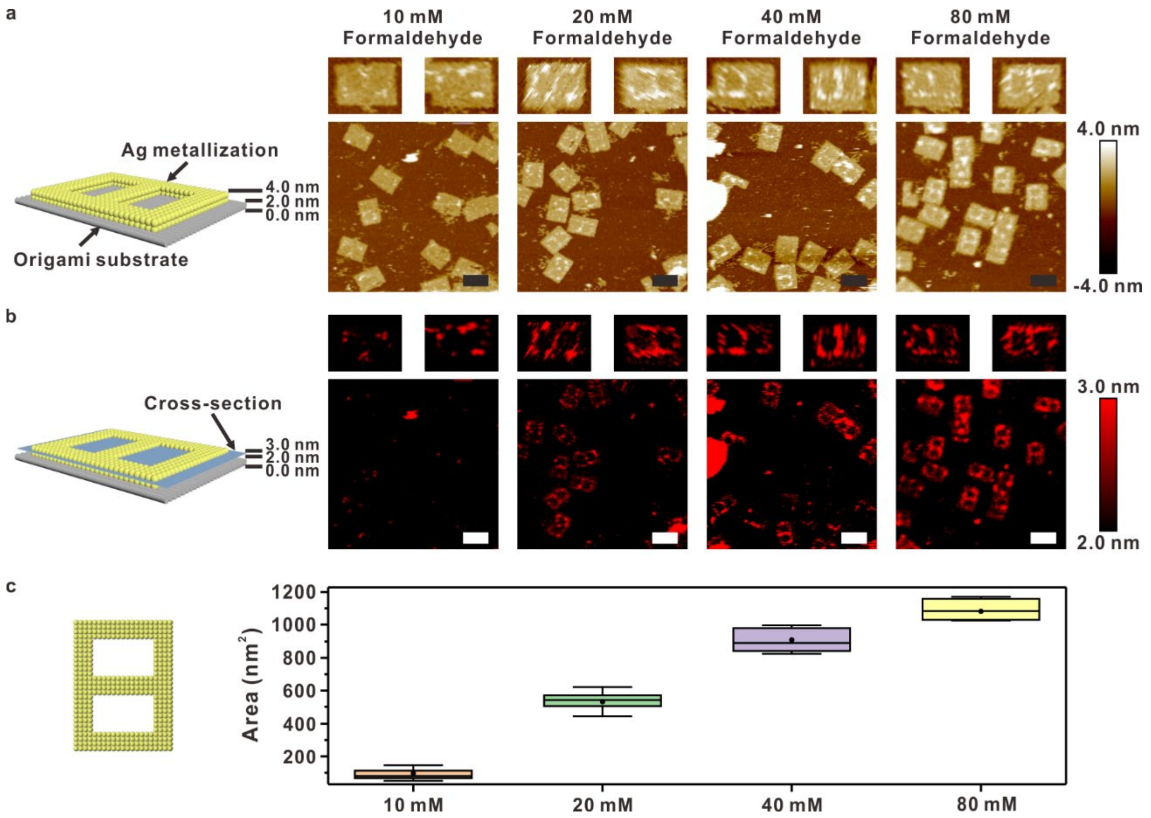

Supplementary Figure 15. Silver nanopatterns constructed on digit 8 patterns with different concentrations of reductant (formaldehyde). Silver precursor concentration: 4 mM $[Ag(NH_3)_2]^+$. Reaction time: 10 min. (a) AFM images of Ag metallization on origami substrate. (b) Tomography images of silver nanopatterns areas above the origami surface. Tomographic of cross-sectional areas at 2 nm to 3 nm height. (c) Quantitative analysis of metal area (N=5, error bars: SD). Scale bars: 100 nm. Source data are provided as a Source Data file.

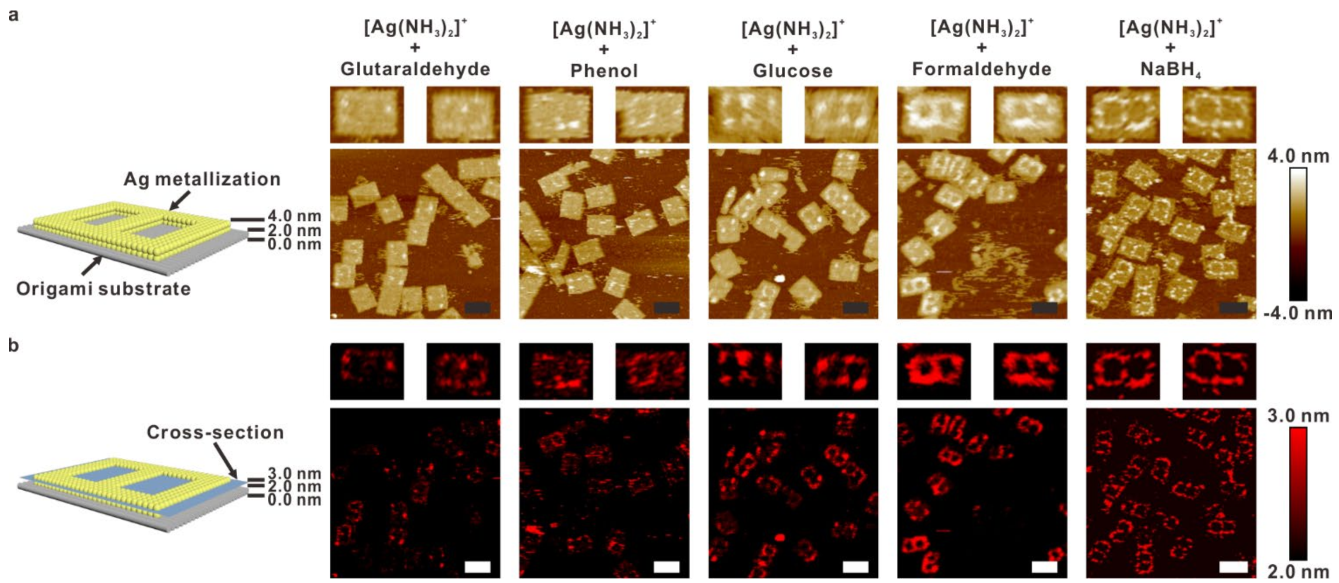

Supplementary Figure 16. Silver nanopatterns constructed on digit 8 patterns with different types of reductants. Silver precursor concentration: 4 mM $[Ag(NH_3)_2]^+$. Reductant concentration: 20 mM. Reaction time: 10 min. (a) AFM images of Ag metallization on origami substrate. (b) Tomography images of silver nanoparticles areas above the origami surface. Tomographic of cross-sectional areas at 2 nm to 3 nm height. Scale bars: 100 nm.

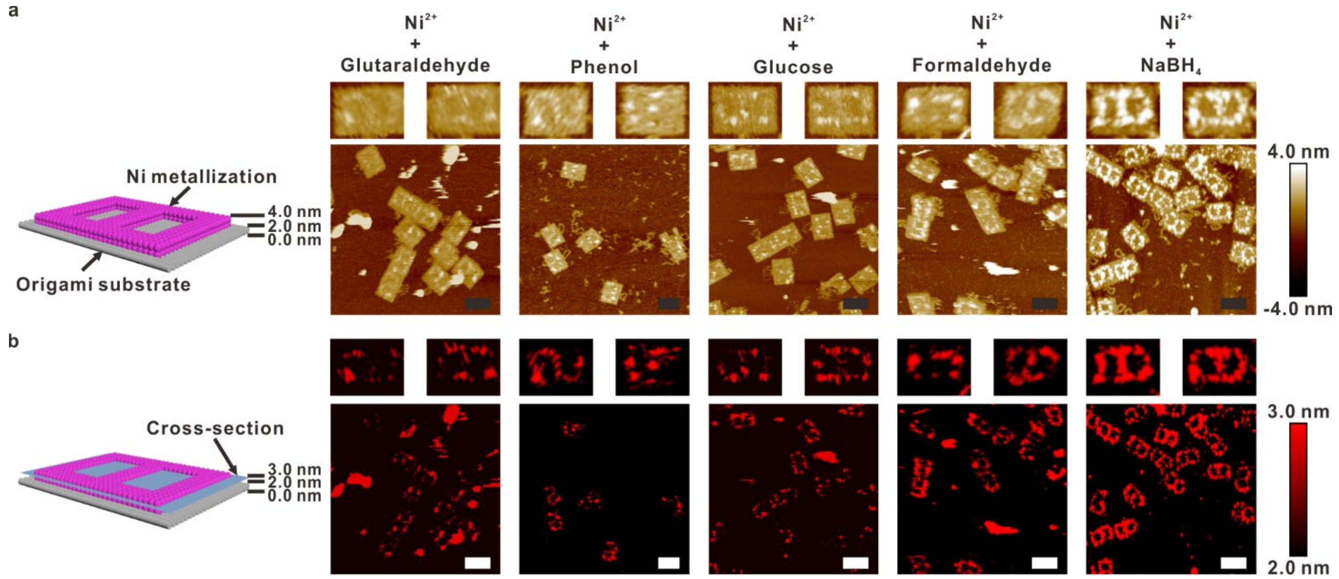

Supplementary Figure 17. Nickel nanopatterns constructed on digit 8 patterns with different types of reductants. Nickel precursor concentration: 4 mM Ni^{2+}. Reductant concentration: 20 mM. Reaction time: 10 min. (a) AFM images of Ni metallization on origami substrate. (b) Tomography images of nickel nanoparticles areas above the origami surface. Tomographic of cross-sectional areas at 2 nm to 3 nm height. Scale bars: 100 nm.

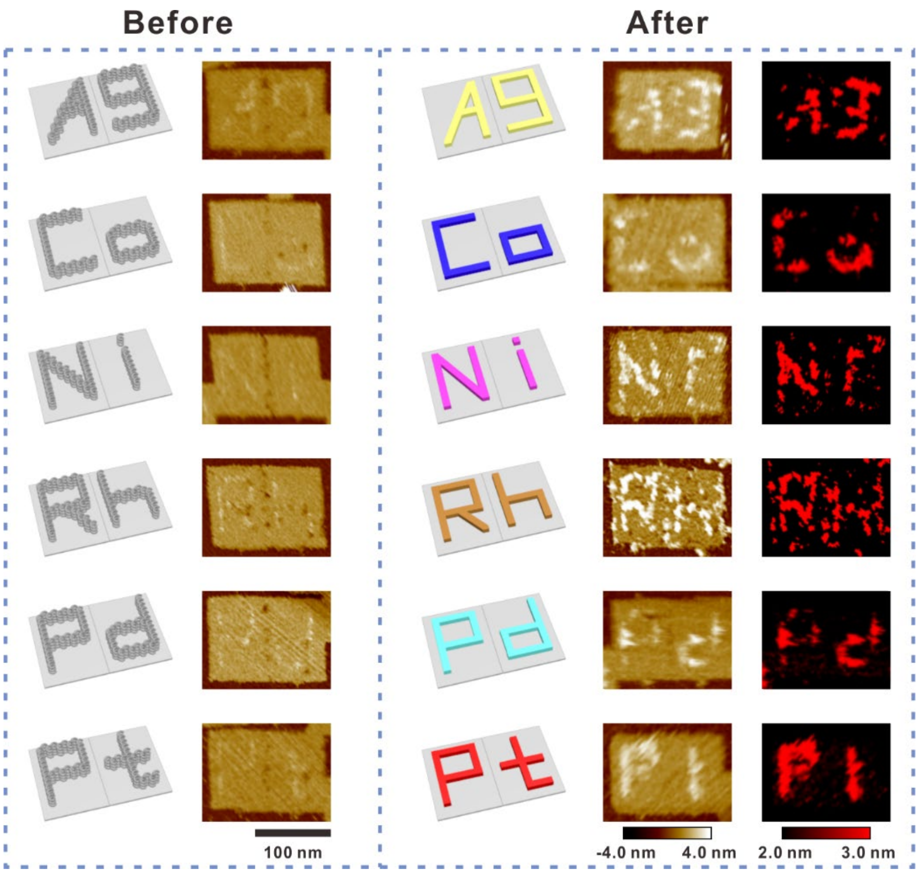

Supplementary Figure 18. Each unary metal nanoparticles on alphabet patterned rectangle origami dimer structure template. Fist column: schematic diagram of elemental symbol patterned of each metal include Ag, Co, Ni, Rh, Pd and Pt before metallization. Second column: AFM images of corresponding rectangle origami dimer structure, and patterns were faintly visible. Third column: schematic diagram of elemental symbol patterned on origami dimer after metallization. Fourth column: AFM images of corresponding metal nanoparticle observed as bright protrusion on origami template after metallization. Fifth column: AFM images depicting metal nanoparticle above the origami surface. Scale bars: 100 nm. Colour scales of AFM images: from -4.0 nm to 4.0 nm. Colour scales of cross-section images: from 2.0 nm to 3.0 nm.

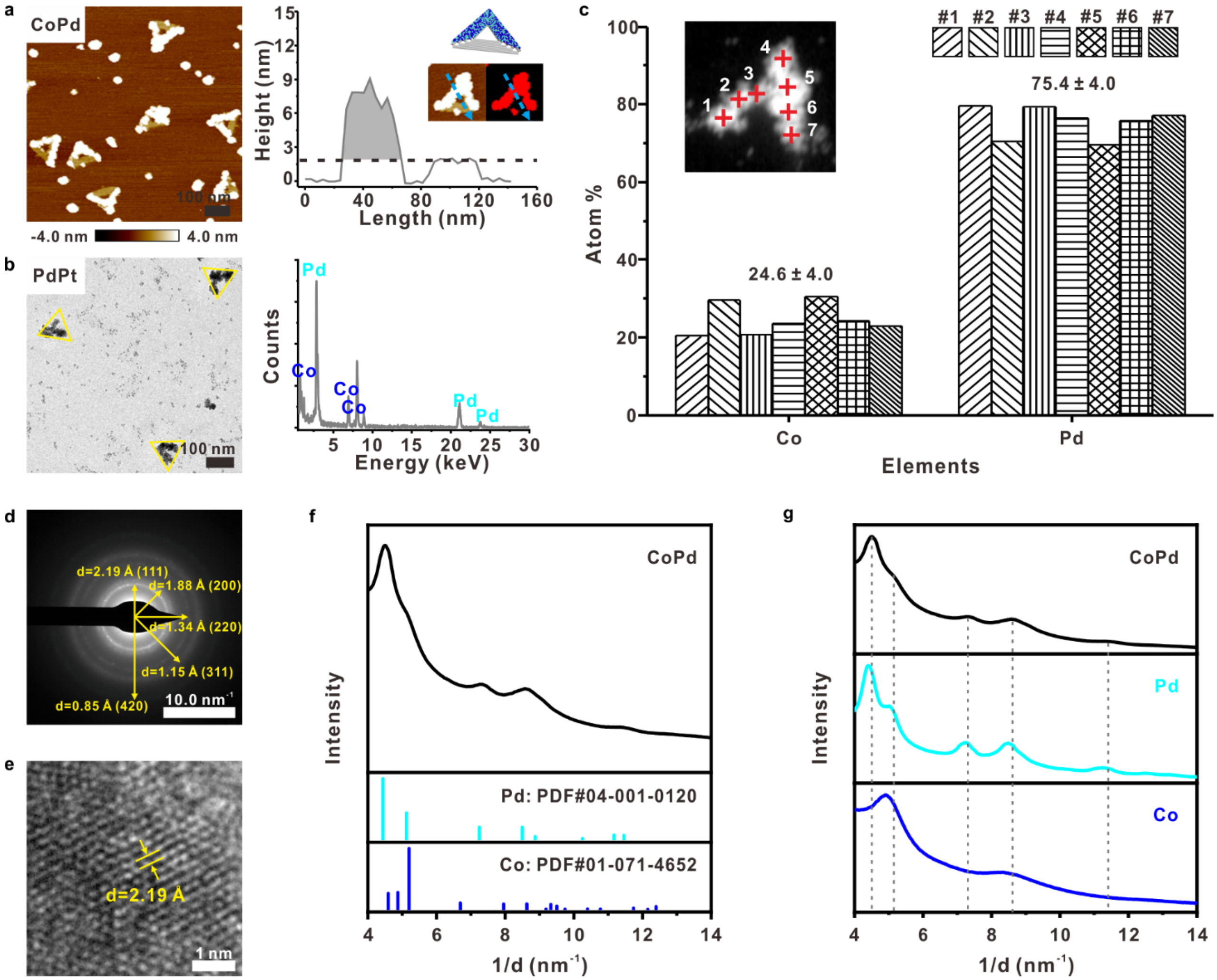

Supplementary Figure 19. Characterization of binary (CoPd) nanoparticles on two sides of triangular DNA origami template after DCIMP processes. (a) AFM images and the corresponding height analysis (nanoparticles average height: ~6.2 nm above the origami surface, average FWHM: ~38.6 nm). (b) TEM image and EDX spectrum analysis. (c) Atomic percentage distributions for each element at different sample regions of a CoPd nanoparticle, the average composition is Co (24.6 ± 4.0%), Pd (75.4 ± 4.0%). (d) SAED image presents ring diffraction pattern of a polycrystalline CoPd specimen. (e) HR-TEM image of a region of CoPd nanoparticle. (f) Diffraction pattern intensity profile of CoPd specimen. (g) Comparison CoPd with individual Co and Pd. Source data are provided as a Source Data file.

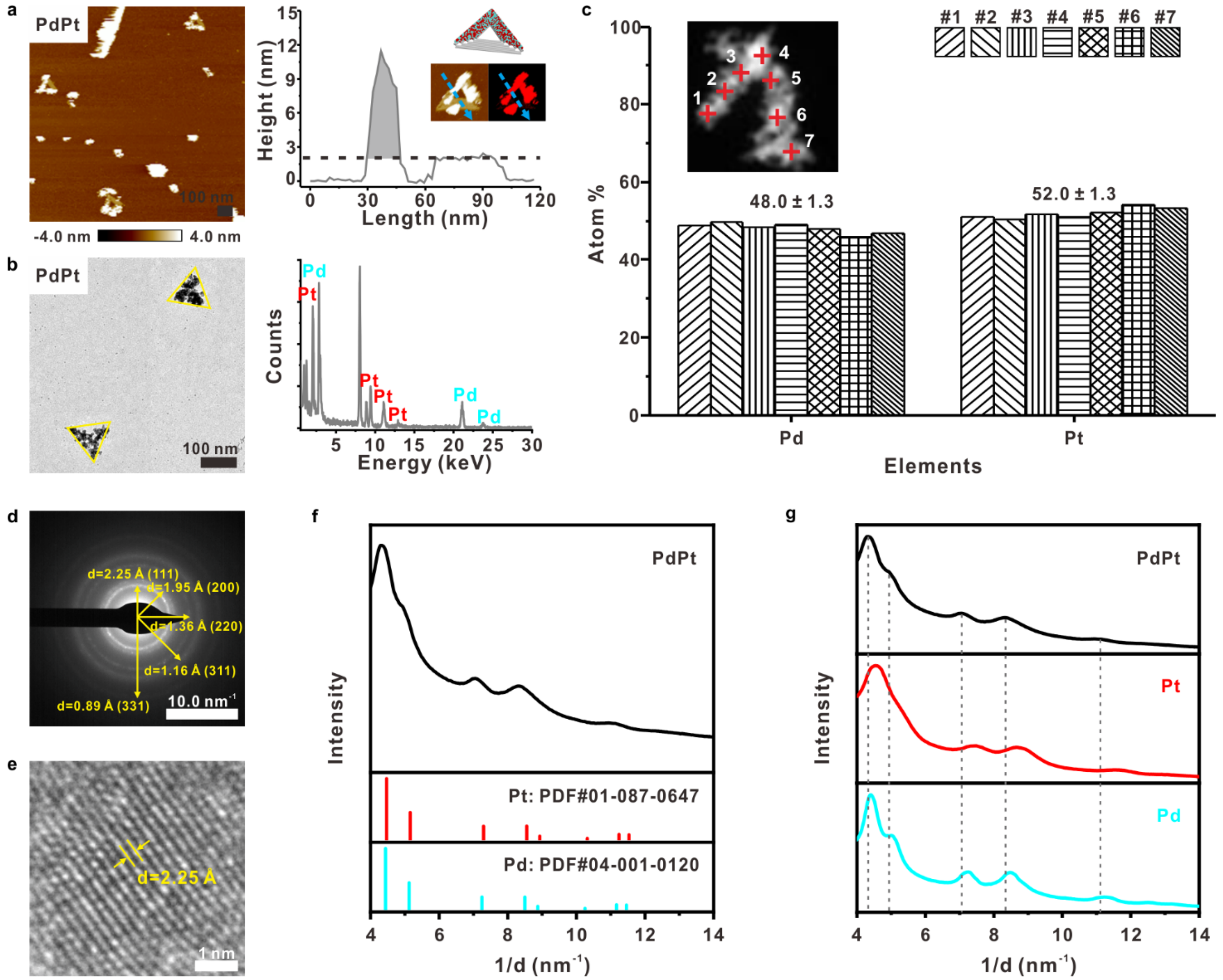

Supplementary Figure 20. Characterization of binary (PdPt) nanoparticles on two sides of triangular DNA origami template after DCIMP processes. (a) AFM images and the corresponding height analysis (nanoparticles average height: ~8.8 nm above the origami surface, average FWHM: ~30.1 nm). (b) TEM image and EDX spectrum analysis. (c) Atomic percentage distributions for each element at different sample regions of a PdPt nanoparticle, the average composition is Pd (48.0 ± 1.3%), Pt (52.0 ± 1.3%). (d) SAED image presents ring diffraction pattern of a polycrystalline PdPt specimen. (e) HR-TEM image of a region of PdPt nanoparticle. (f) Diffraction pattern intensity profile of PdPt specimen. (g) Comparison PdPt with individual Pd and Pt. Source data are provided as a Source Data file.

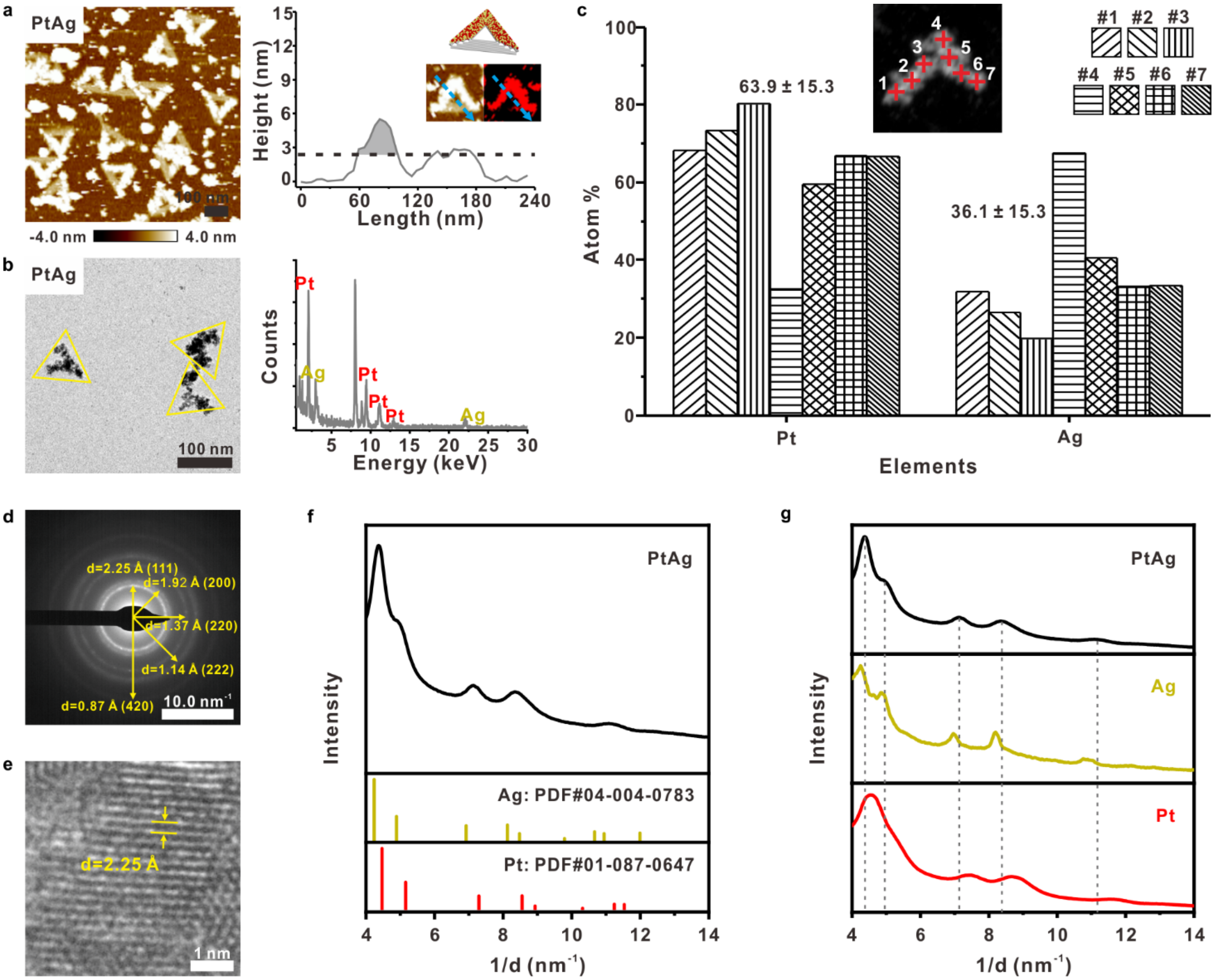

Supplementary Figure 21. Characterization of binary (PtAg) nanoparticles on two sides of triangular DNA origami template after DCIMP processes. (a) AFM images and the corresponding height analysis (nanoparticles average height: ~2.5 nm above the origami surface, average FWHM: ~32.5 nm). (b) TEM image and EDX spectrum analysis. (c) Atomic percentage distributions for each element at different sample regions of a PtAg nanoparticle, the average composition is Pt (63.9 ± 15.3%), Ag (36.1 ± 15.3%). (d) SAED image presents ring diffraction pattern of a polycrystalline PtAg specimen. (e) HR-TEM image of a region of PtAg nanoparticle. (f) Diffraction pattern intensity profile of PtAg specimen. (g) Comparison PtAg with individual Pt and Ag. Source data are provided as a Source Data file.

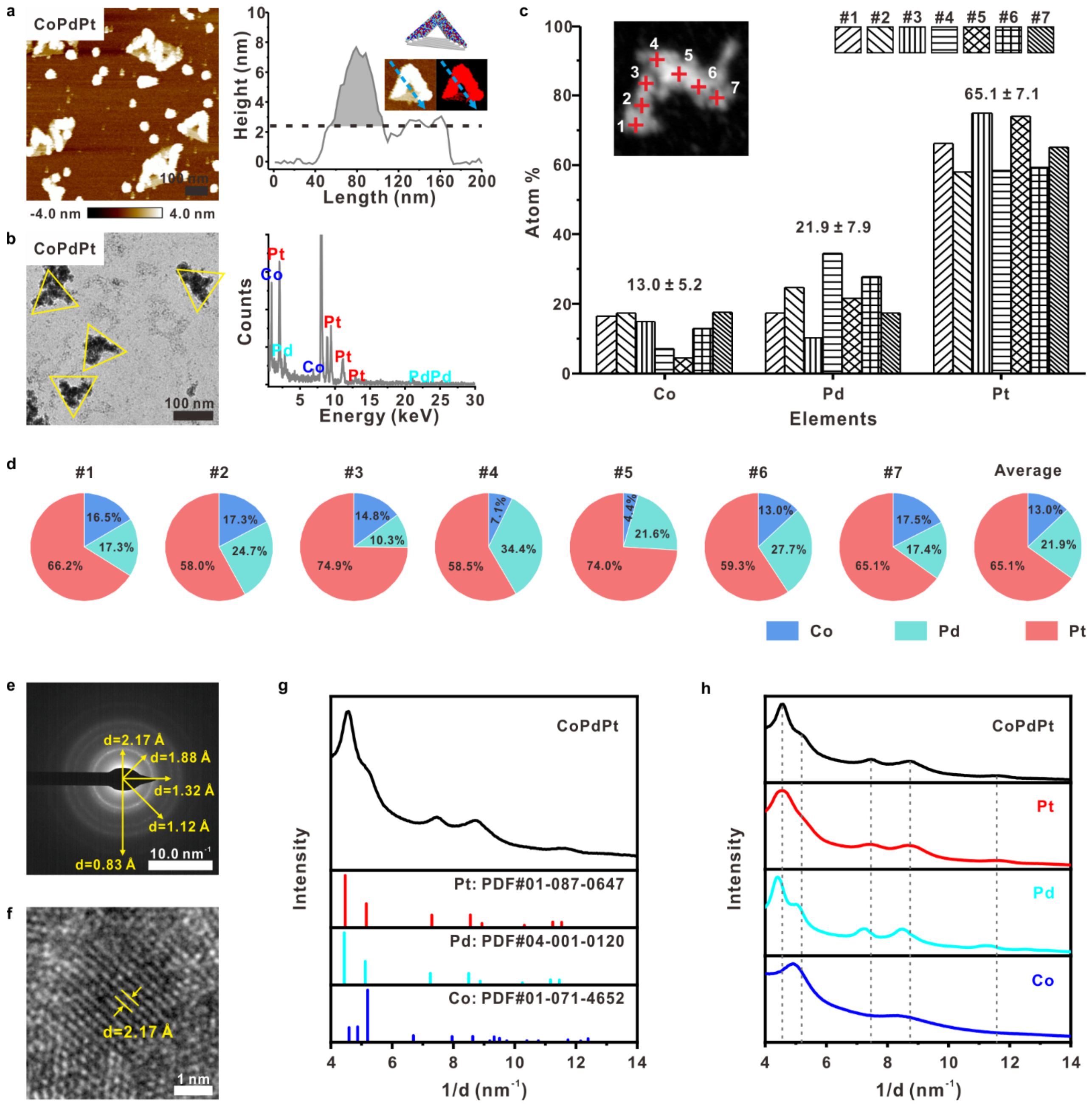

Supplementary Figure 22. Characterization of ternary (CoPdPt) nanoparticles on two sides of triangular DNA origami template after DCIMP processes. (a) AFM images and the corresponding height analysis (nanoparticles average height: ~4.6 nm above the origami surface, average FWHM: ~33.2 nm). (b) TEM image and EDX spectrum analysis. (c and d) Atomic percentage distributions for each element at different sample regions of a CoPdPt nanoparticle, the average composition is Co (13.0 ± 5.2%), Pd (21.9 ± 7.9%), Pt (65.1 ± 7.1%). (e) SAED image presents ring diffraction pattern of a polycrystalline CoPdPt specimen. (f) HR-TEM image of a region of CoPdPt nanoparticle. (g) Diffraction pattern intensity profile of CoPdPt specimen. (h) Comparison CoPdPt with individual Co, Pd and Pt. Source data are provided as a Source Data file.

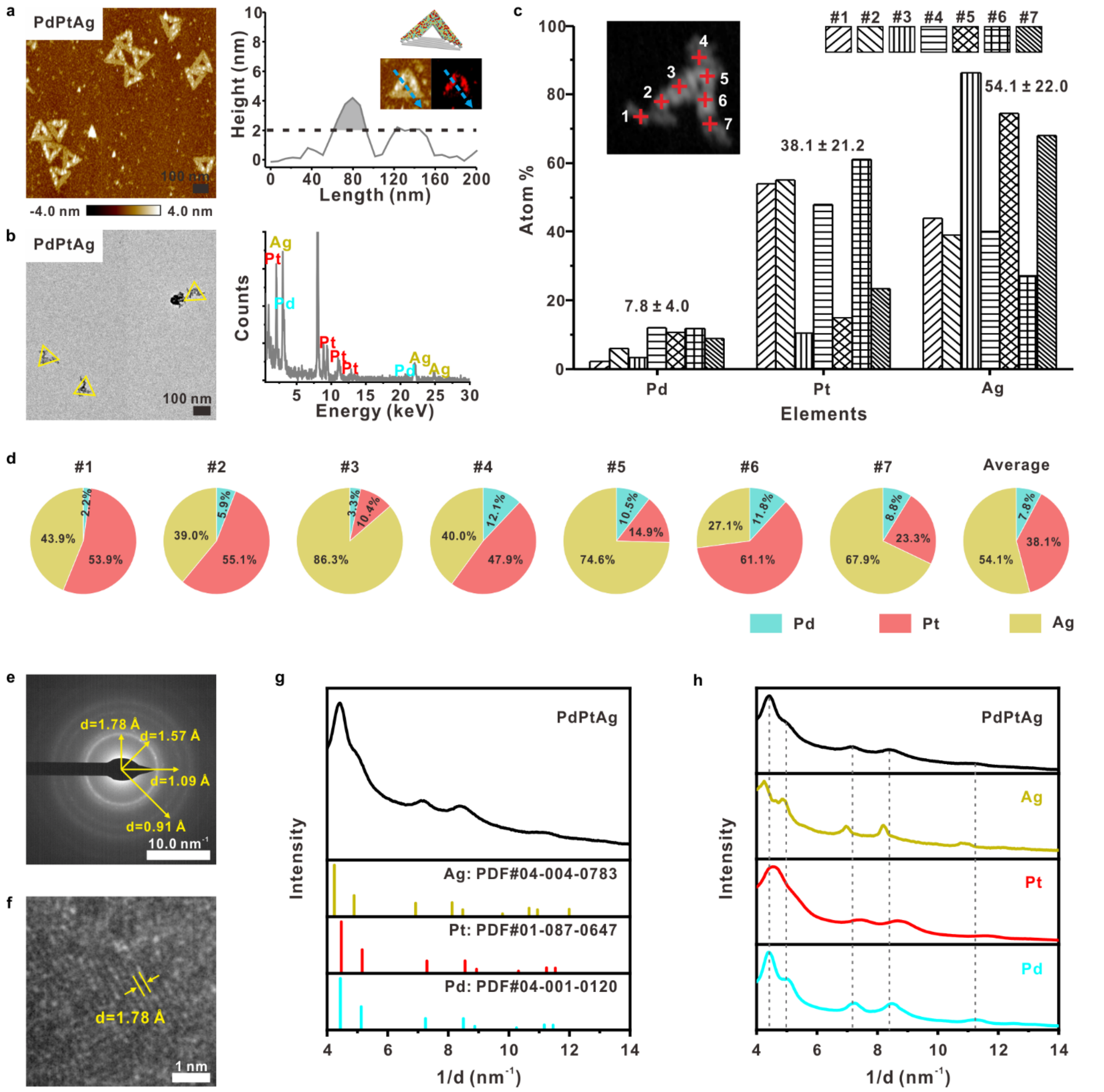

Supplementary Figure 23. Characterization of ternary (PdPtAg) nanoparticles on two sides of triangular DNA origami template after DCIMP processes. (a) AFM images and the corresponding height analysis (nanoparticles average height: ~2.0 nm above the origami surface, average FWHM: ~34.1 nm). (b) TEM image and EDX spectrum analysis. (c and d) Atomic percentage distributions for each element at different sample regions of a PdPtAg nanoparticle, the average composition is Pd (7.8 ± 4.0%), Pt (38.1 ± 21.2%), Ag (54.1 ± 22.0%). (e) SAED image presents ring diffraction pattern of a polycrystalline PdPtAg specimen. (f) HR-TEM image of a region of PdPtAg nanoparticle. (g) Diffraction pattern intensity profile of PdPtAg specimen. (h) Comparison PdPtAg with individual Pd, Pt and Ag. Source data are provided as a Source Data file.

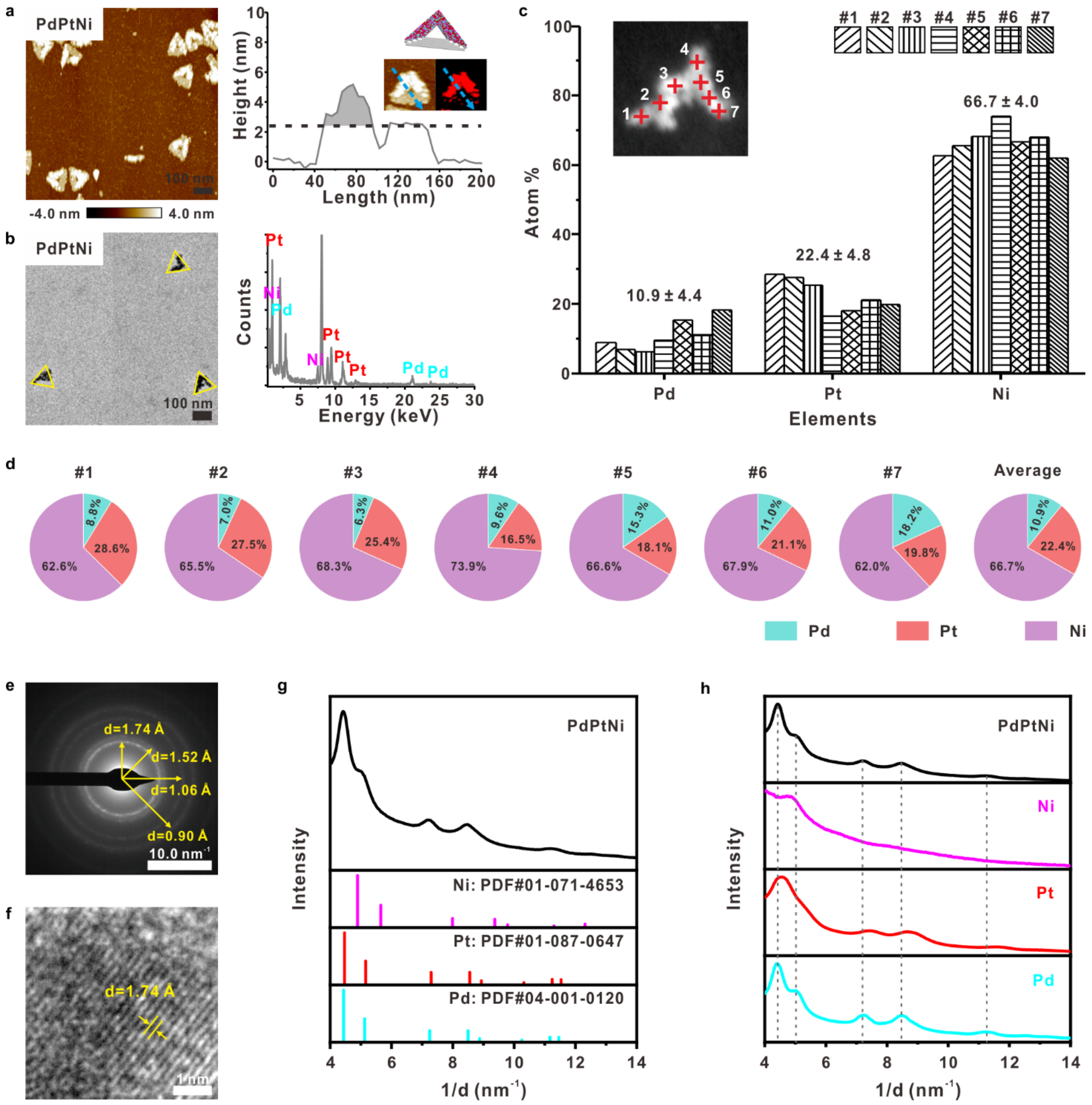

Supplementary Figure 24. Characterization of ternary (PdPtNi) nanoparticles on two sides of triangular DNA origami template after DCIMP processes. (a) AFM images and the corresponding height analysis (nanoparticles average height: ~2.6 nm above the origami surface, average FWHM: ~35.6 nm). (b) TEM image and EDX spectrum analysis. (c and d) Atomic percentage distributions for each element at different sample regions of a PdPtNi nanoparticle, the average composition is Pd (10.9 ± 4.4%), Pt (22.4 ± 4.8%), Ni (66.7 ± 4.0%). (e) SAED image presents ring diffraction pattern of a polycrystalline PdPtNi specimen. (f) HR-TEM image of a region of PdPtNi nanoparticle. (g) Diffraction pattern intensity profile of PdPtNi specimen. (h) Comparison PdPtNi with individual Pd, Pt and Ni. Source data are provided as a Source Data file.

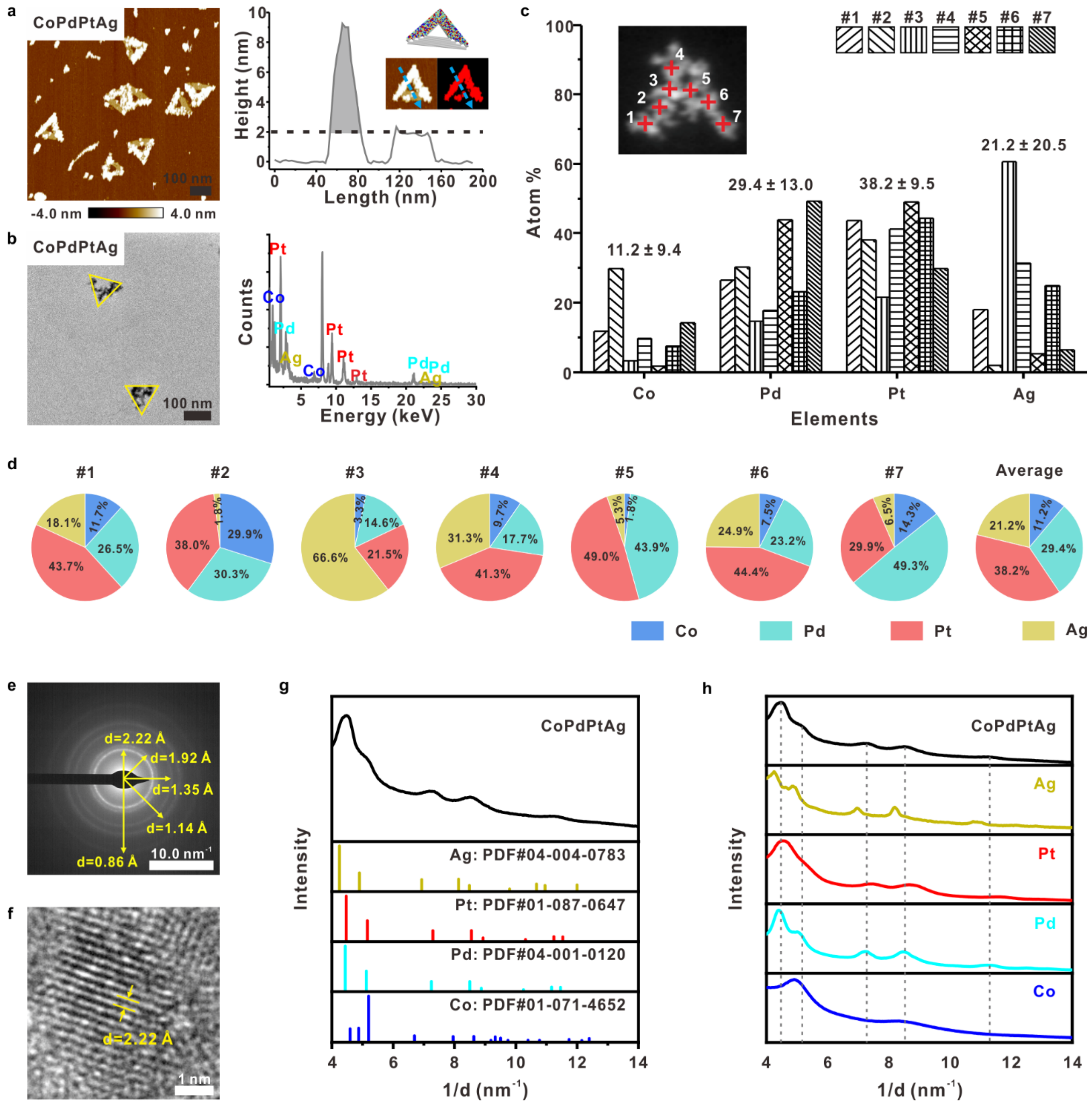

Supplementary Figure 25. Characterization of quaternary (CoPdPtAg) nanoparticles on two sides of triangular DNA origami template after DCIMP processes. (a) AFM images and the corresponding height analysis (nanoparticles average height: ~6.2 nm above the origami surface, average FWHM: ~31.7 nm). (b) TEM image and EDX spectrum analysis. (c and d) Atomic percentage distributions for each element at different sample regions of a CoPdPtAg nanoparticle, the average composition is Co (11.2 ± 9.4%), Pd (29.4 ± 13.0%), Pt (38.2 ± 9.5%), Ag (21.2 ± 20.5%). (e) SAED image presents ring diffraction pattern of a polycrystalline CoPdPtAg specimen. (f) HR-TEM image of a region of CoPdPtAg nanoparticle. (g) Diffraction pattern intensity profile of CoPdPtAg specimen. (h) Comparison CoPdPtAg with individual Co, Pd, Pt and Ni. Source data are provided as a Source Data file.

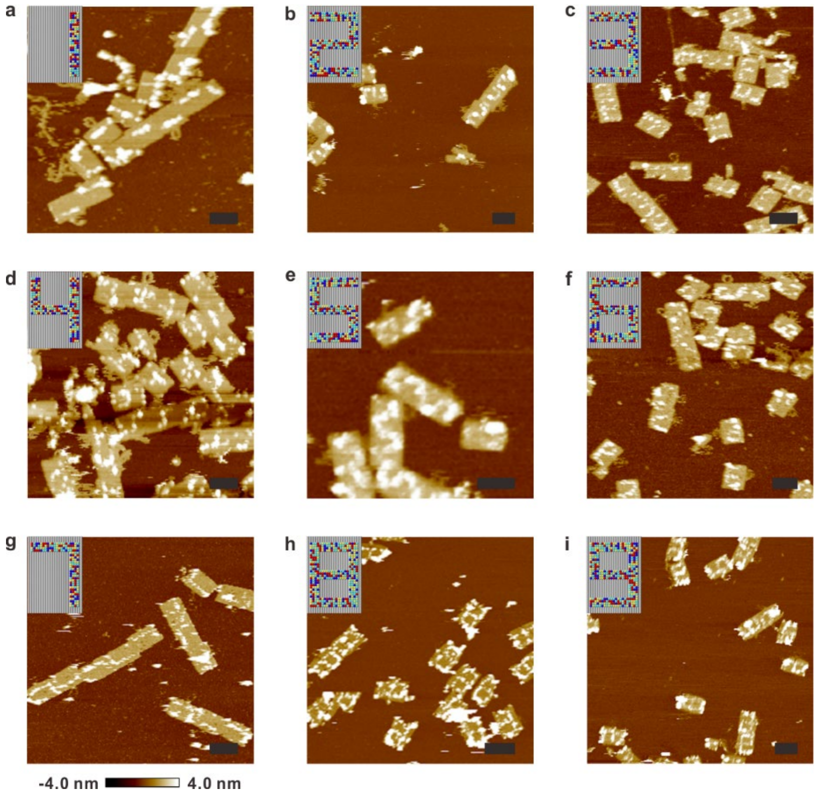

Supplementary Figure 26. AFM images showing digit nanopatterns from 1 to 9 fabricated by quaternary (CoPdPtAg): (a) digit ‘1’, (b) digit ‘2’, (c) digit ‘3’, (d) digit ‘4’, (e) digit ‘5’, (f) digit ‘6’, (g) digit ‘7’, (h) digit ‘8’, (i) digit ‘9’. Scale bar: 100 nm. Colour scales of AFM images: from -4.0 nm to 4.0 nm.

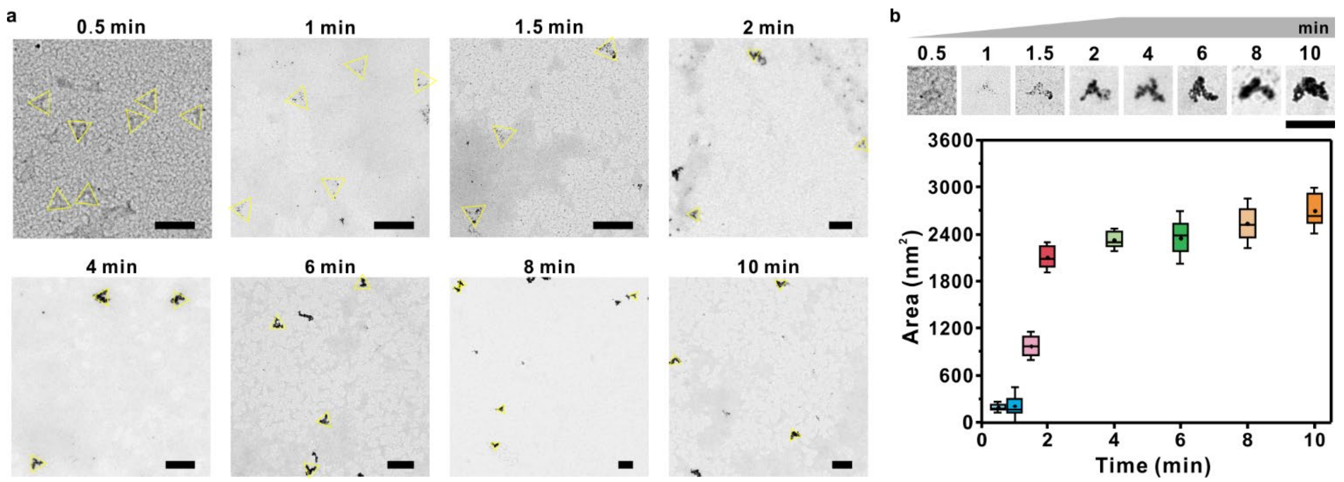

Supplementary Figure 27. CoPdPtAgNi morphological evolutions on triangular DNA origami template at different reaction time points. (a) TEM image. (b) Representative structure and quantitative analysis of the Pd area at different reaction time points (N=5, error bars: SD). Scale bars: 200 nm. Source data are provided as a Source Data file.

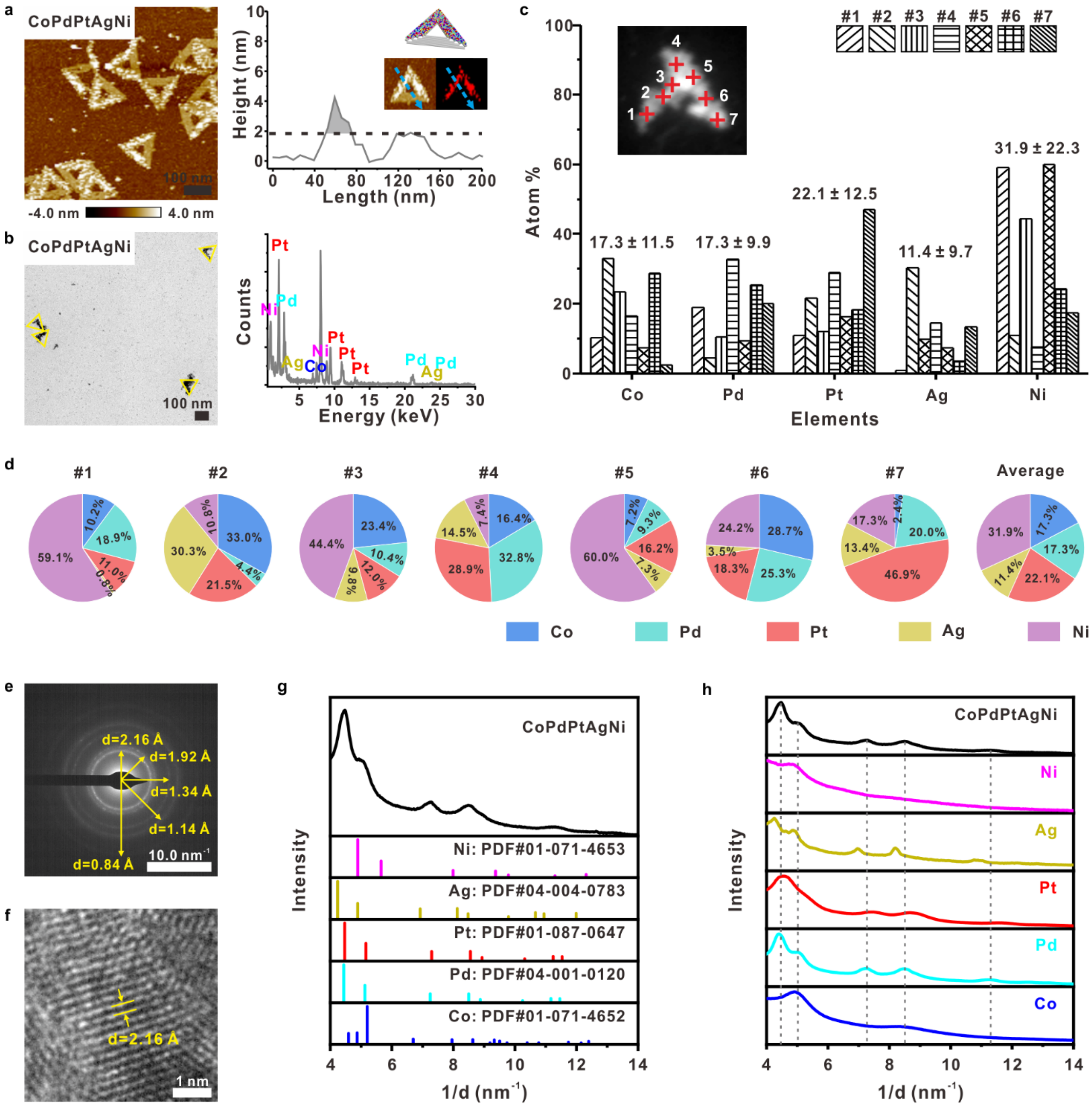

Supplementary Figure 28. Characterization of quinary (CoPdPtAgNi) nanoparticles on two sides of triangular DNA origami template after DCIMP processes. (a) AFM images and the corresponding height analysis (nanoparticles average height: ~2.4 nm above the origami surface, average FWHM: ~25.5 nm). (b) TEM image and EDX spectrum analysis. (c and d) Atomic percentage distributions for each element at different sample regions of a CoPdPtAgNi nanoparticle, the average composition is Co (17.3 ± 11.5%), Pd (17.3 ± 9.9%), Pt (22.1 ± 12.5%), Ag (11.4 ± 9.7%), Ni (31.9 ± 22.3%). (e) SAED image presents ring diffraction pattern of a polycrystalline CoPdPtAgNi specimen. (f) HR-TEM image of a region of CoPdPtAgNi nanoparticle. (g) Diffraction pattern intensity profile of CoPdPtAgNi specimen. (h) Comparison CoPdPtAg with individual Co, Pd, Pt, Ag and Ni. Source data are provided as a Source Data file.

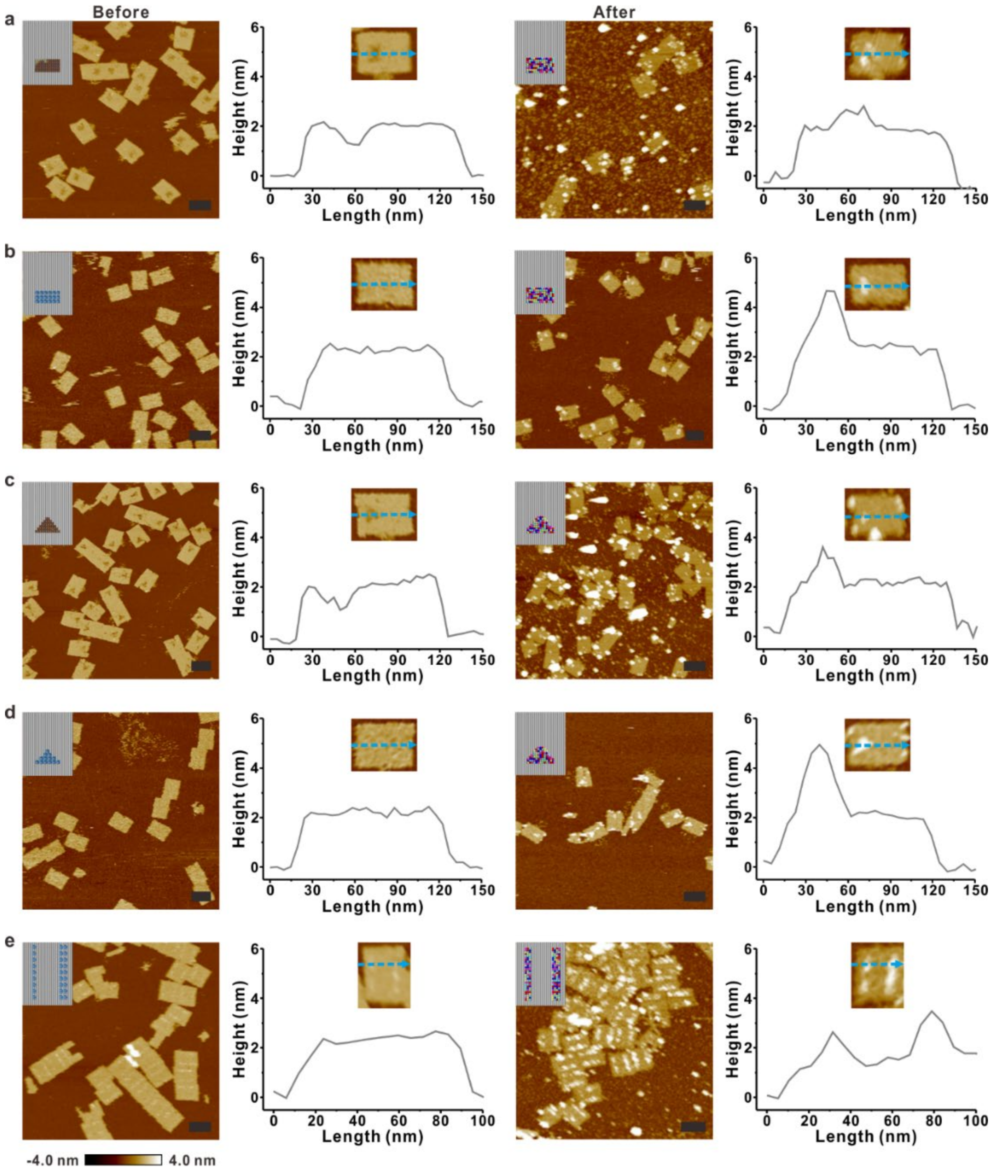

Supplementary Figure 29. AFM images of the quinary alloy (CoPdPtAgNi) constructed on several different origami templates and the corresponding height analysis. (a) Metallization on a DNA origami template with a rectangular hole (depth of hole: ~0.8 nm below the origami surface, height of quinary nanoparticles: ~0.8 nm above the origami surface. Quinary nanoparticles height: ~1.6 nm). (b) Metallization on a rectangular template with protruding pcDNA (quinary nanoparticles height: ~2.1 nm above the origami surface). (c) Metallization on a DNA origami template with a triangular hole (depth of hole: ~0.8 nm below the origami surface, height of quinary nanoparticles: ~1.1 nm above the origami surface. Quinary nanoparticles height: ~1.9 nm). (d) Metallization on a triangular template with protruding pcDNA (quinary nanoparticles height: ~2.5 nm above the origami surface). (e) Metallization on single and double lines of pcDNA on a rectangular DNA origami template (Linewidth of quinary nanoparticles on single line pcDNA: ~10.5 nm, ~1.3 nm high above the origami surface. Linewidth of quinary nanoparticles on double line pcDNA: ~15.8 nm, ~1.7 nm high above the origami surface). Scale bars: 100 nm. Colour scales of AFM images: from -4.0 nm to 4.0 nm. Source data are provided as a Source Data file.

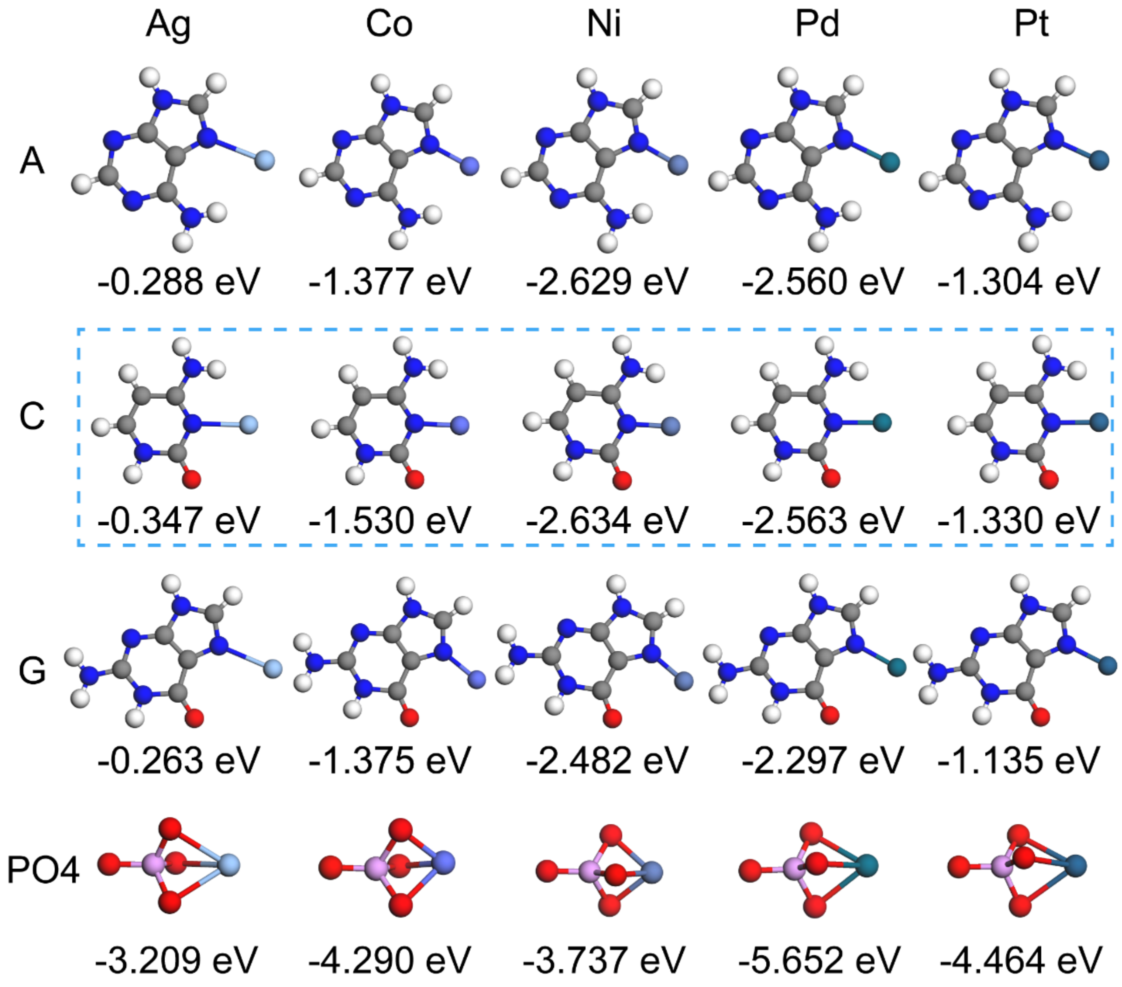

Supplementary Figure 30. Geometric optimized structures and corresponding binding energy between metal atoms and bases from DFT calculations. The blue dash box indicated the most stable complexes from the three bases.

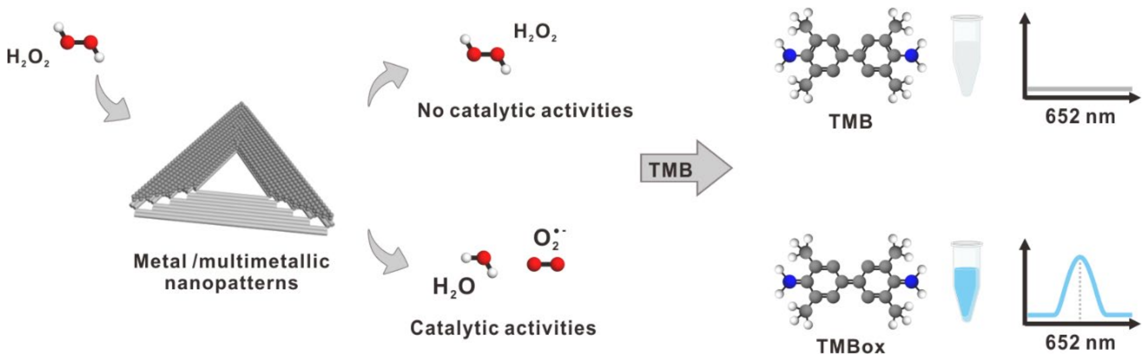

Supplementary Figure 31. Schematic of the peroxidase-like catalytic process.

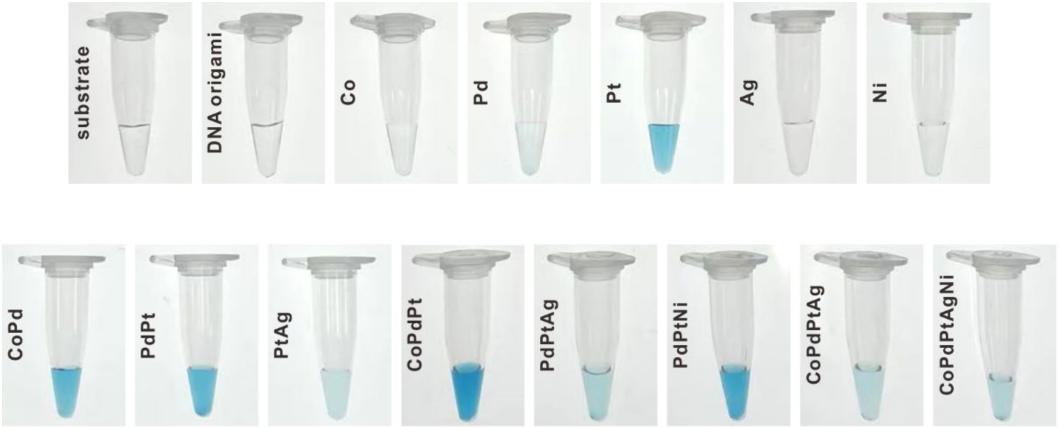

Supplementary Figure 32. Photos of TMB solution after 2 minutes of catalytic reaction.

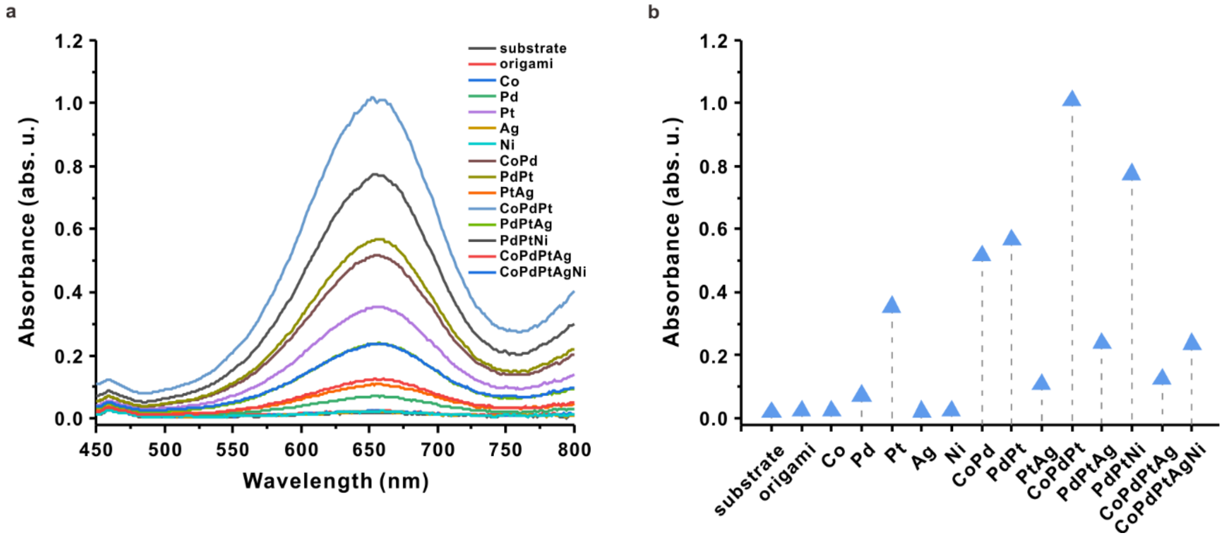

Supplementary Figure 33. Catalytic behaviours of metal and multimetallic nanopatterns. (a) Absorption spectra of nanopattern samples in reaction system. (b) Absorption at 652 nm. Source data are provided as a Source Data file.

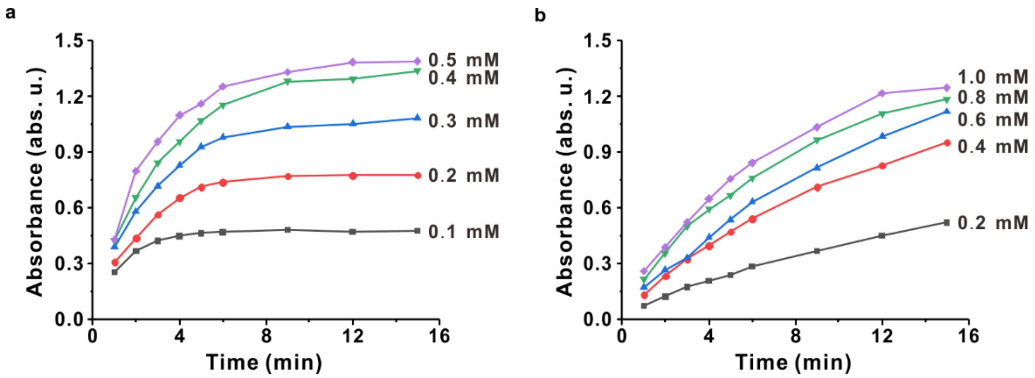

Supplementary Figure 34. Monitoring of time-dependent catalytic kinetics of CoPdPt nanopatterns in different conditions. (a) Constant 20 mM H_2O_2 with different concentrations of TMB (0.1-0.5 mM). (b) Constant 1 mM TMB with different concentrations of H_2O_2 (0.2-1.0 mM). Source data are provided as a Source Data file.

Supplementary Table 1 Coordination bond energies (in eV) of metal ions (geometric optimized structure) to DNA.

**Metal ions**	**A**	**G**	**C**	**T**	**Phosphate**
Magnesium: $[Mg(H_2O)_4]^{2+}$	-0.74	-0.80	-0.59	-0.31	-1.05
Silver: $[Ag(H_2O)]^{+}$	-1.03	-1.31	-1.20	-0.41	0.12
Cobalt: $Co(H_2O)_2Cl_2$	-1.21	-0.89	-0.76	-0.71	-1.43
Nickel: $Ni(H_2O)_2Cl_2$	-1.15	-1.21	-1.65	-0.87	-1.83
Palladium: $[PdCl_4]^{2-}$	-2.83	-2.34	-2.09	-0.79	-1.90
Platinum: $[PtCl_4]^{2-}$	-2.98	-2.10	-2.87	-1.08	-1.44

Supplementary Table 2 Oxidation-reduction potentials of metal ion precursor**.**

**Metal**	Co^{2+}/Co	$[PtCl_4]^{2-}/Pt$	$[PdCl_4]^{2-}/Pd$	Ag^{+}/Ag	Ni^{2+}/Ni
$\mathbf{E^0}$	-0.28 V	0.755 V	0.591 V	0.7996 V	-0.257 V

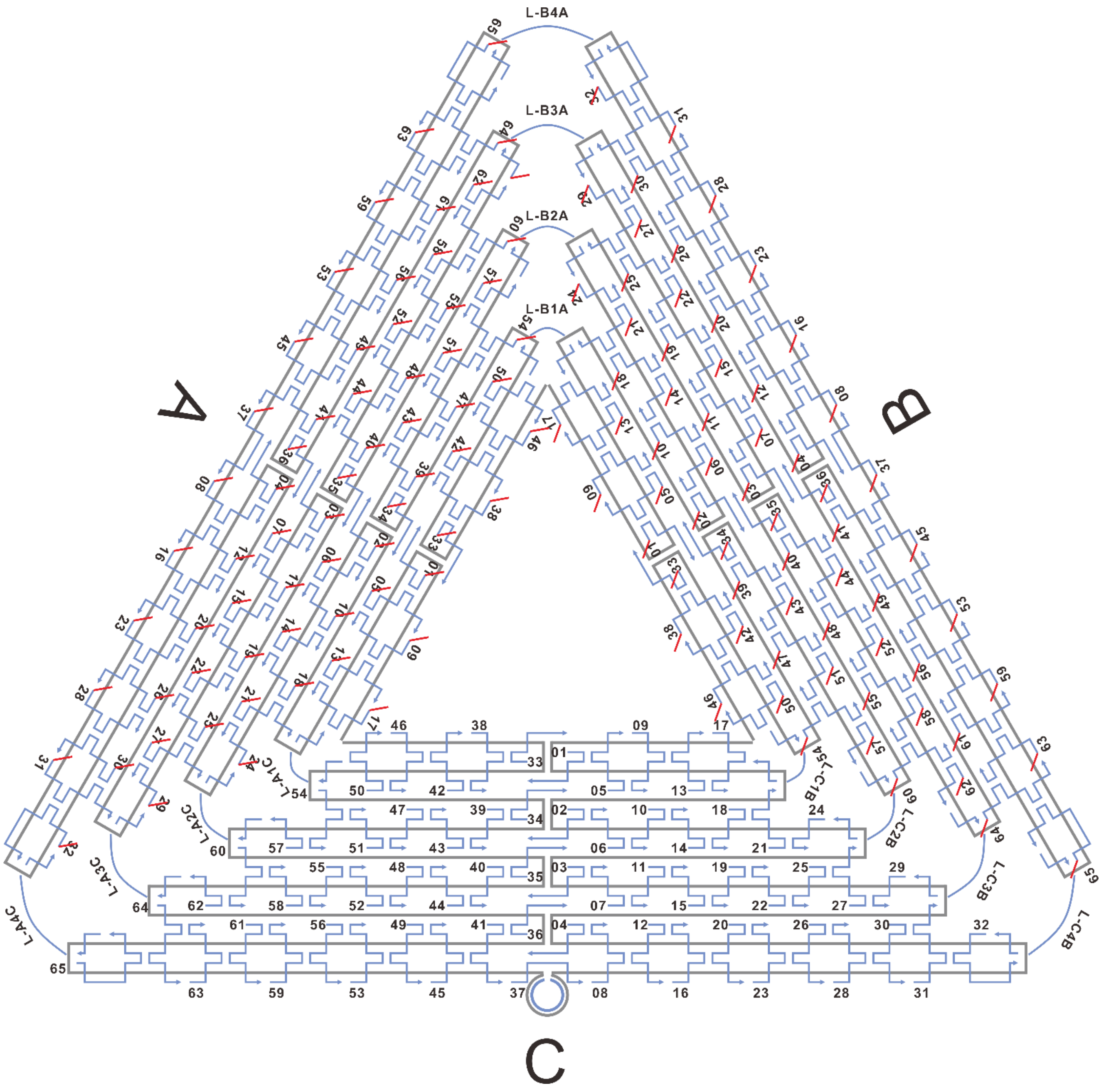

Supplementary Figure 35. Schematic of pcDNA on two sides pattern of triangular DNA origami template.

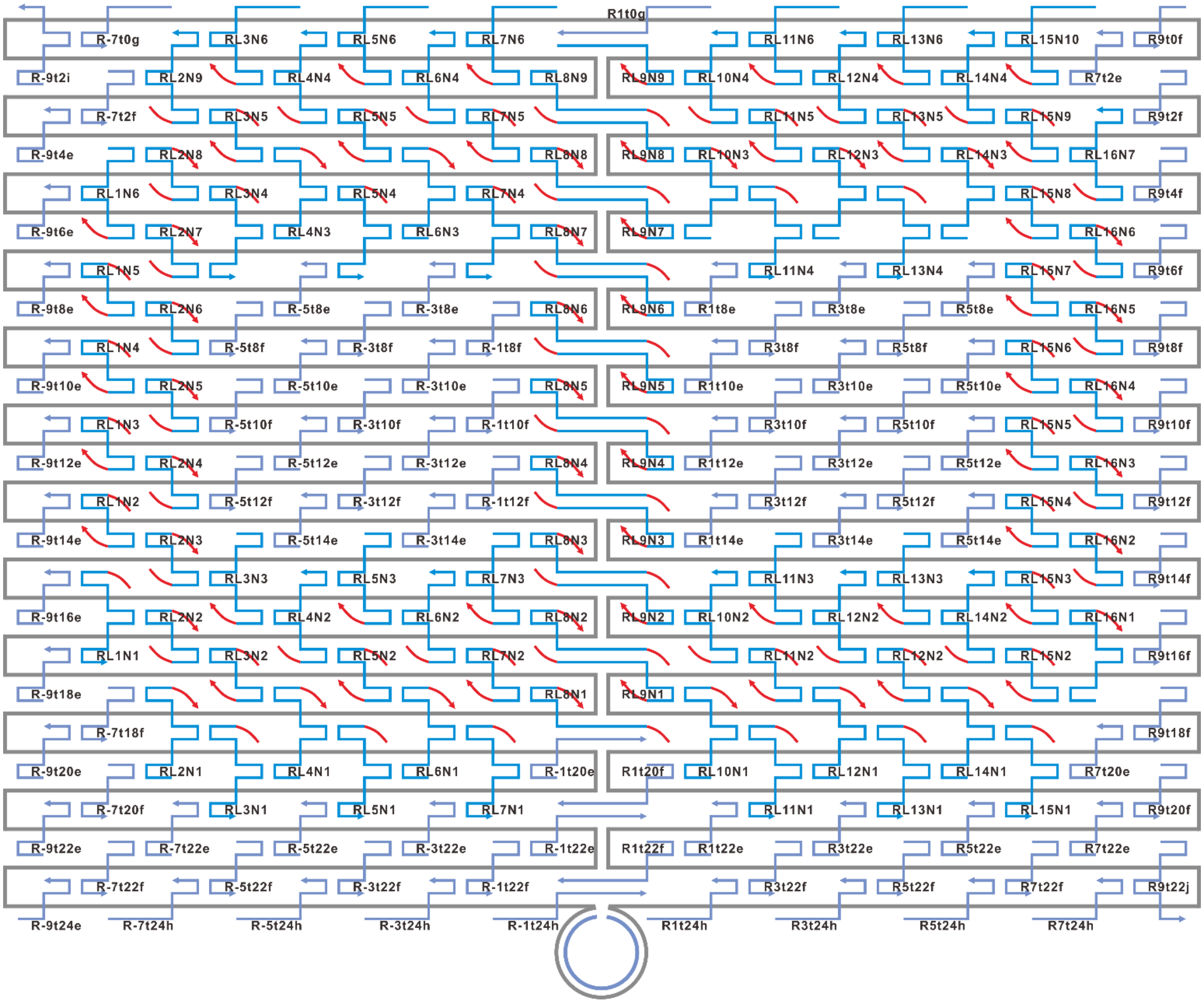

Supplementary Figure 36. Schematic of pcDNA on digit 8 pattern of rectangular DNA origami template.

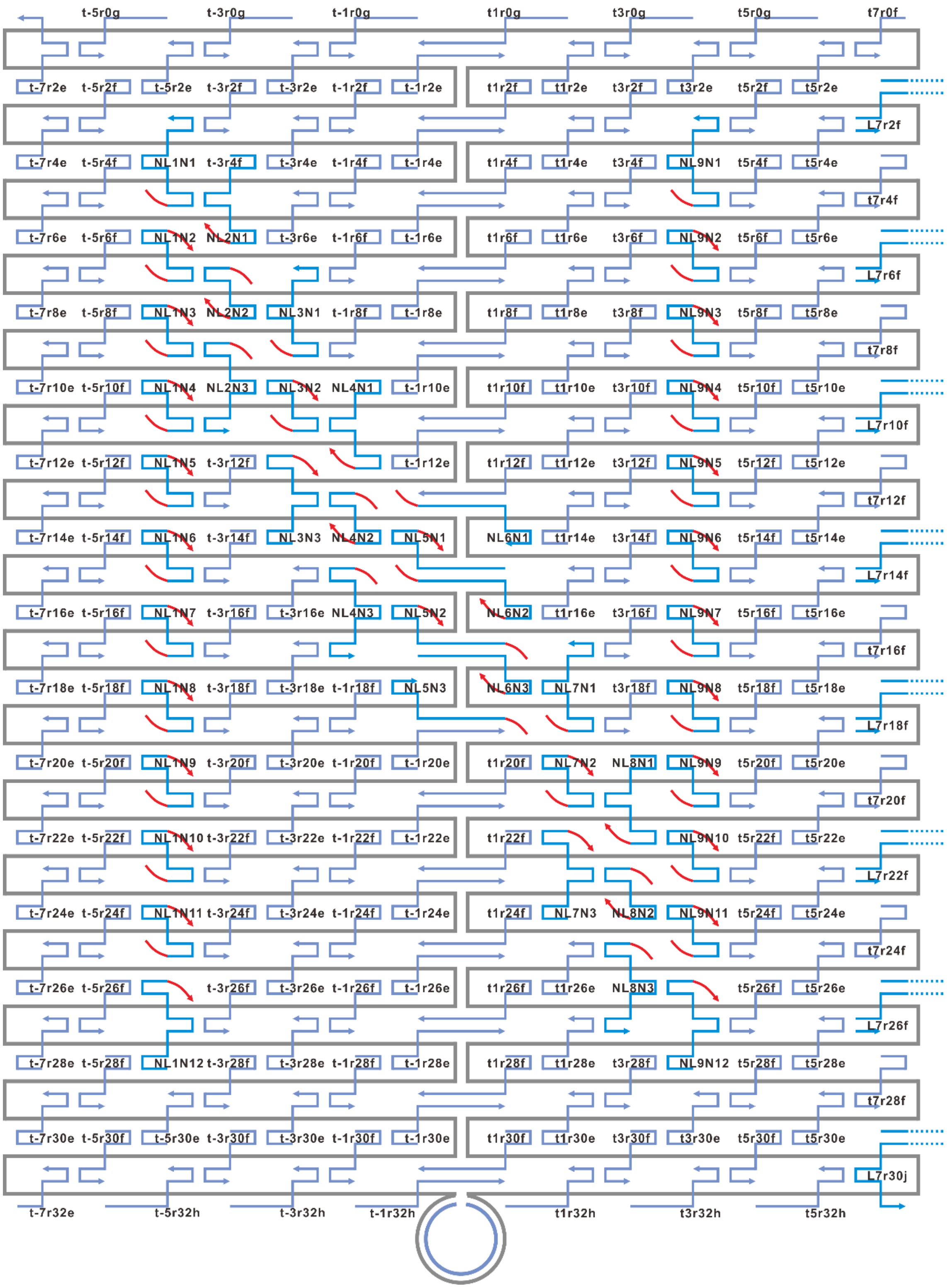

Supplementary Figure 37. Schematic of pcDNA on alphabet 'N' pattern of tall rectangular DNA origami template.

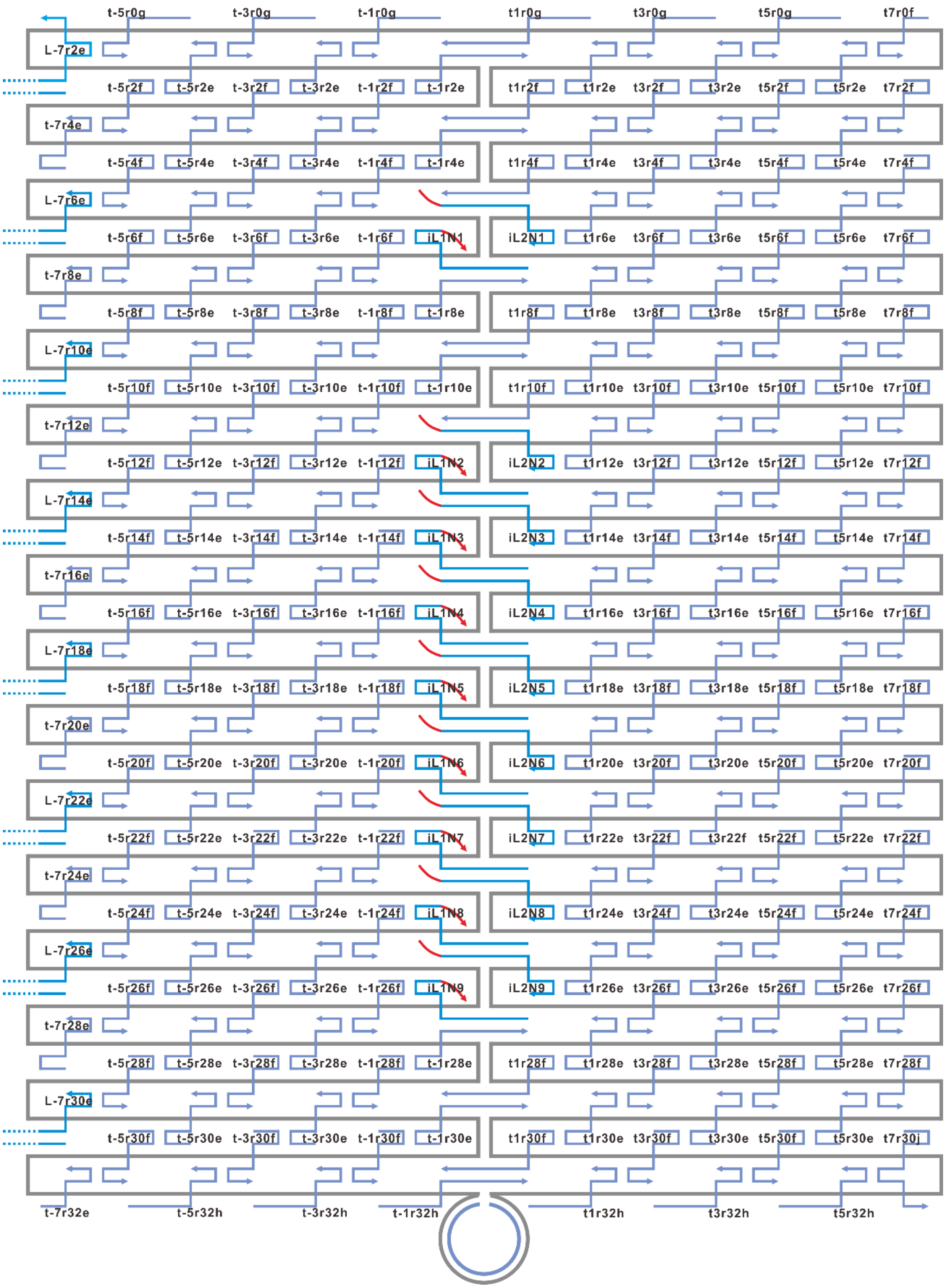

Supplementary Figure 38. Schematic of pcDNA on alphabet ‘i’ pattern of tall rectangular DNA origami template.

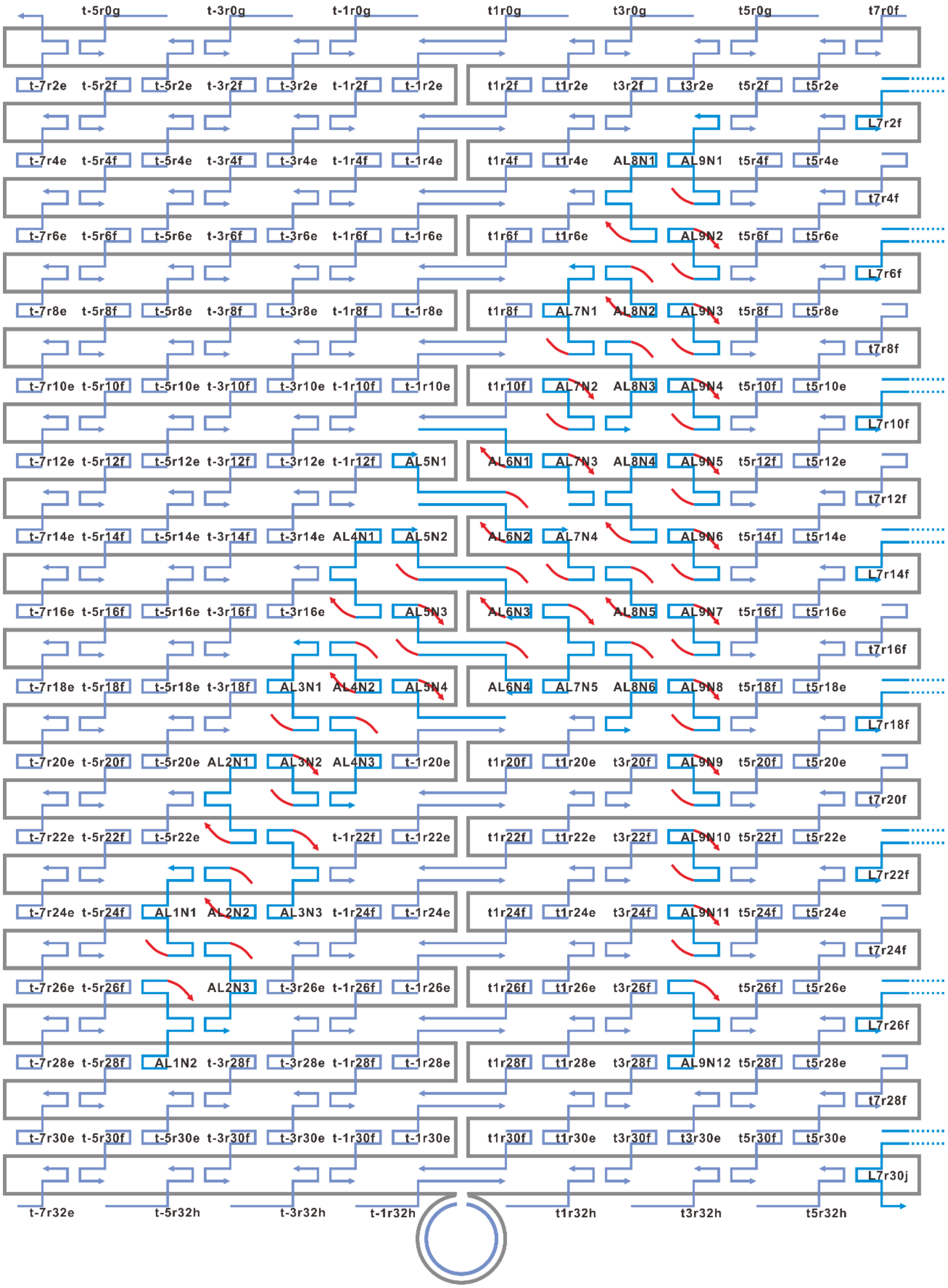

Supplementary Figure 39. Schematic of pcDNA on alphabet 'A' pattern of tall rectangular DNA origami template.

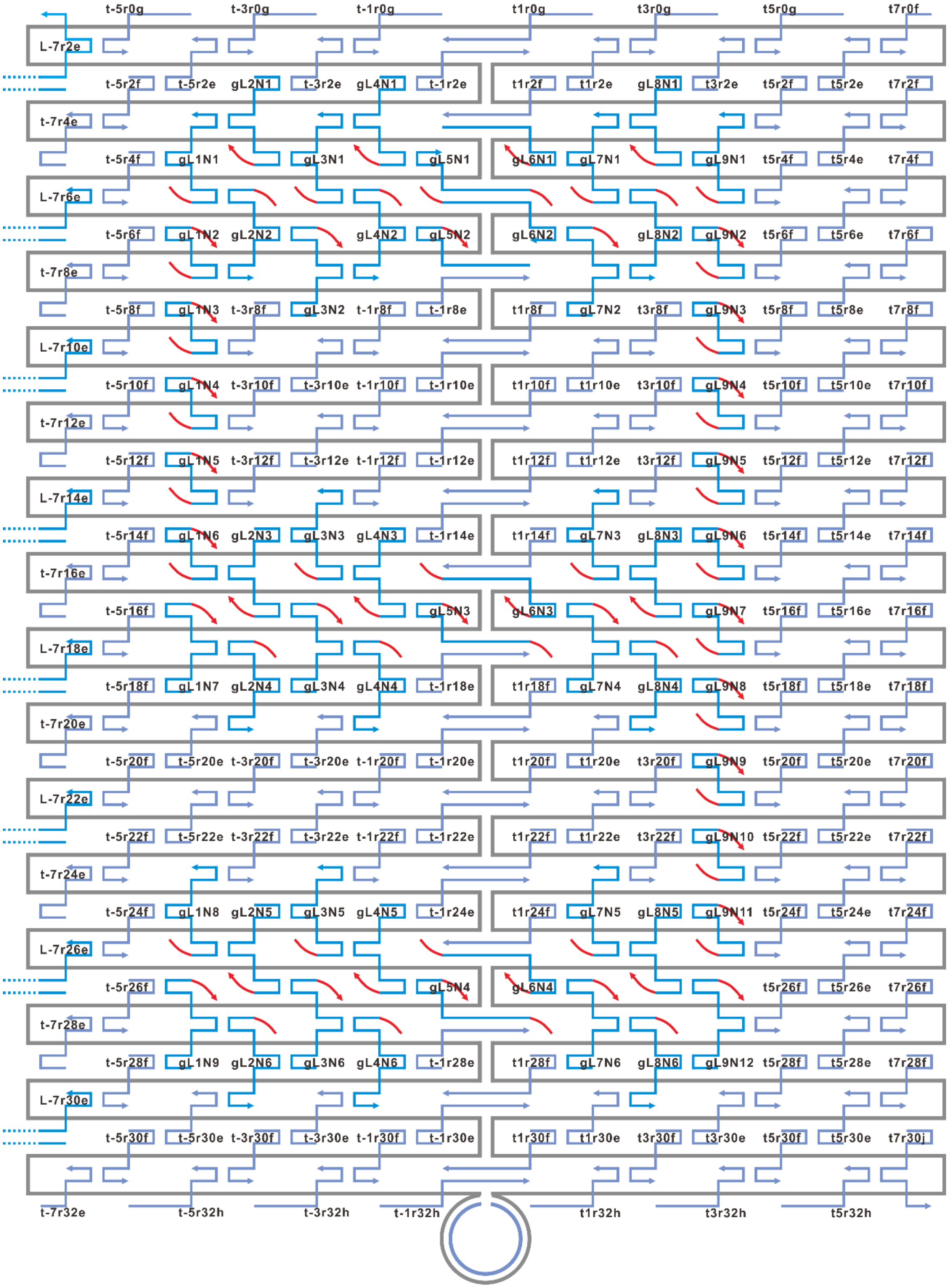

Supplementary Figure 40. Schematic of pcDNA on alphabet ‘g’ pattern of tall rectangular DNA origami template.

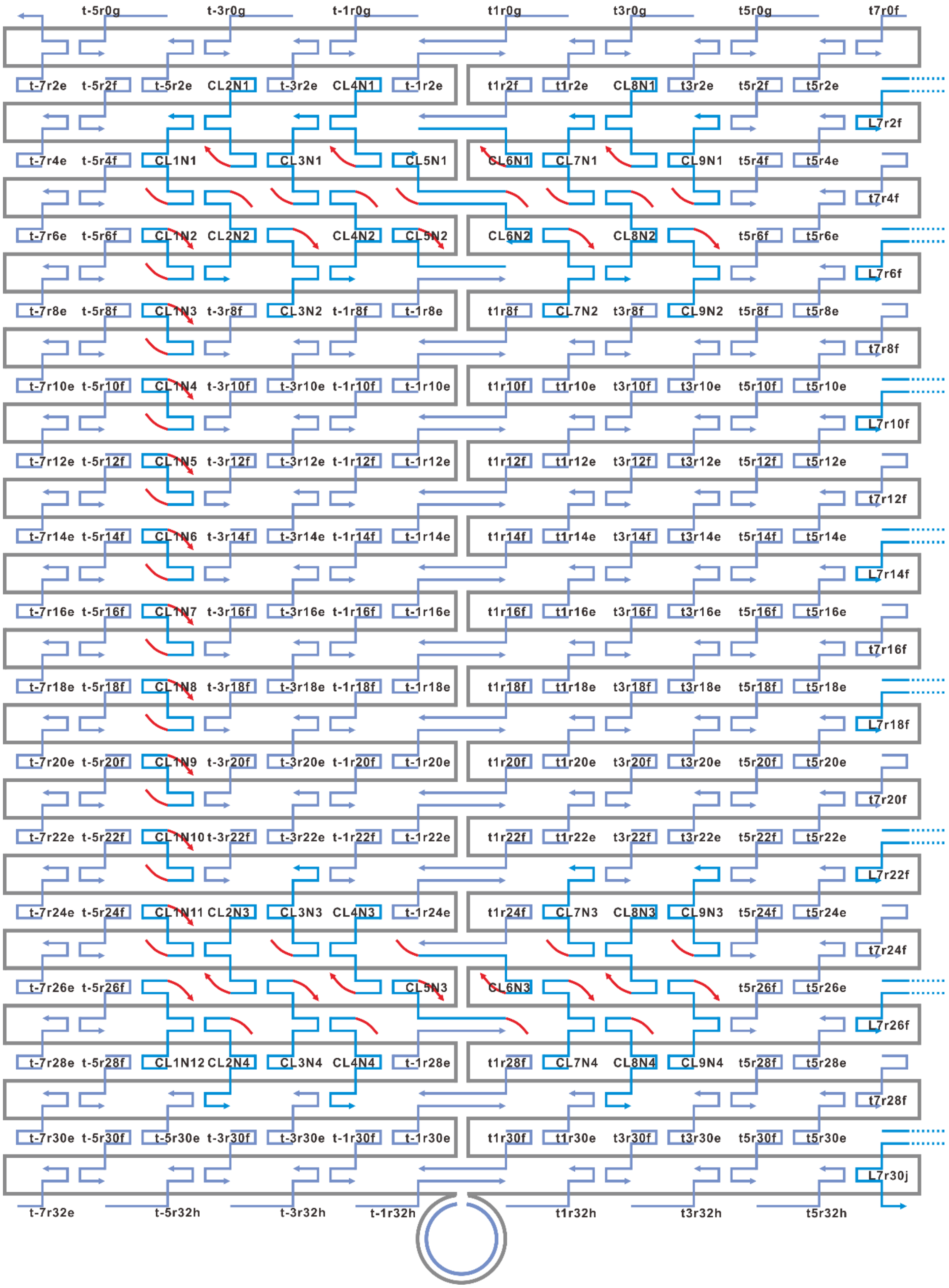

Supplementary Figure 41. Schematic of pcDNA on alphabet 'C' pattern of tall rectangular DNA origami template.

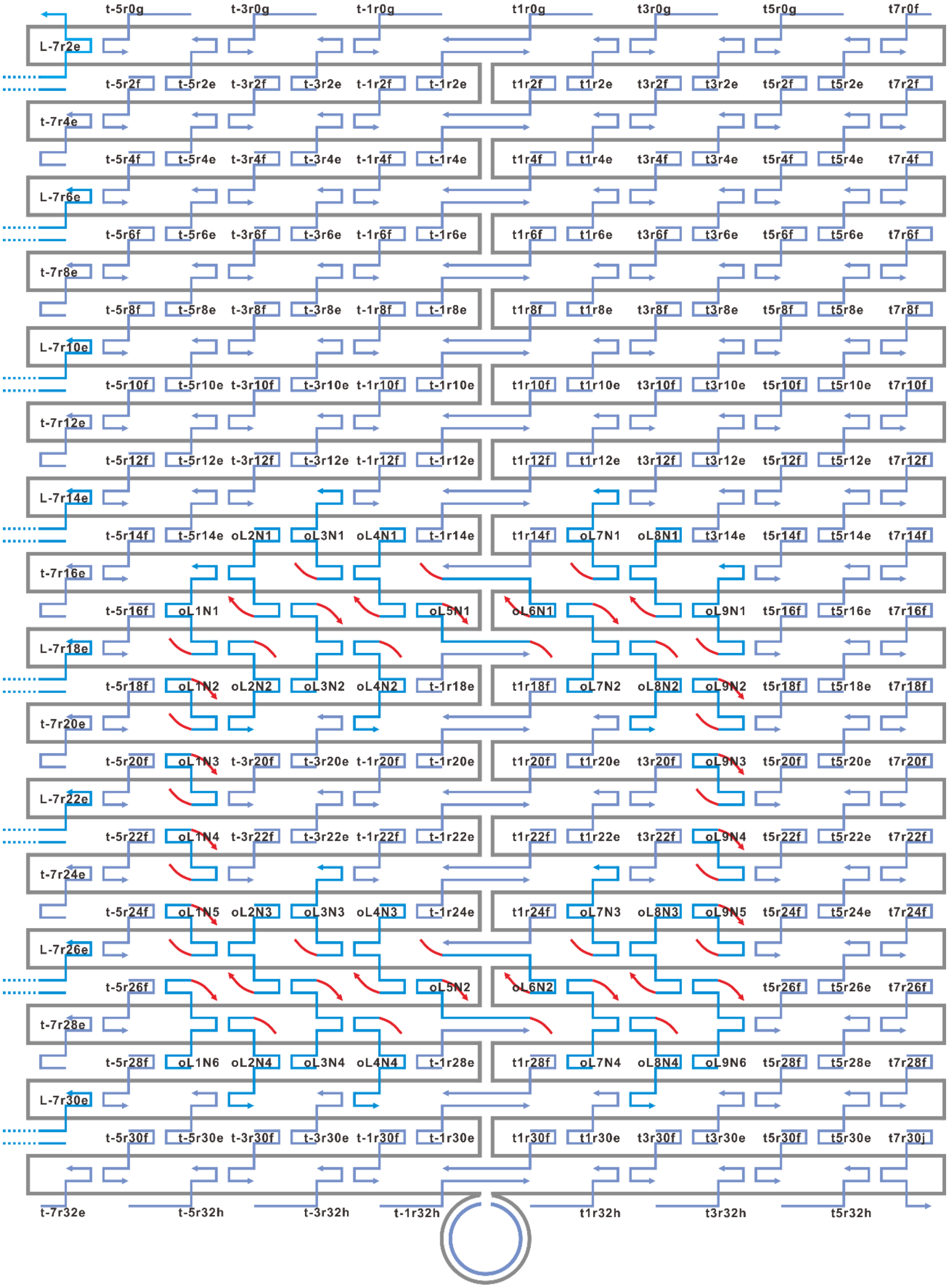

Supplementary Figure 42. Schematic of pcDNA on alphabet ‘o’ pattern of tall rectangular DNA origami template.

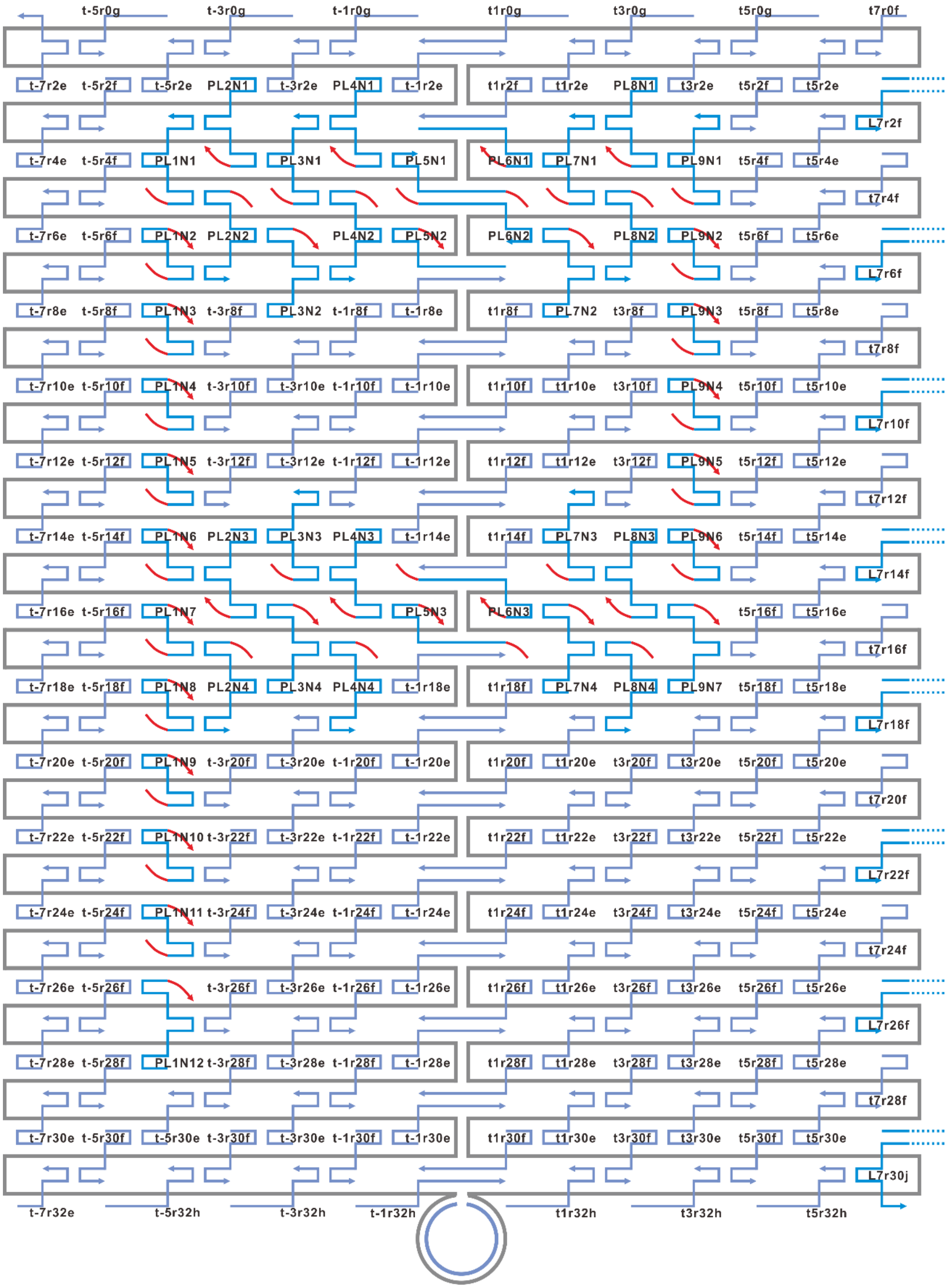

Supplementary Figure 43. Schematic of pcDNA on alphabet 'P' pattern of tall rectangular DNA origami template.

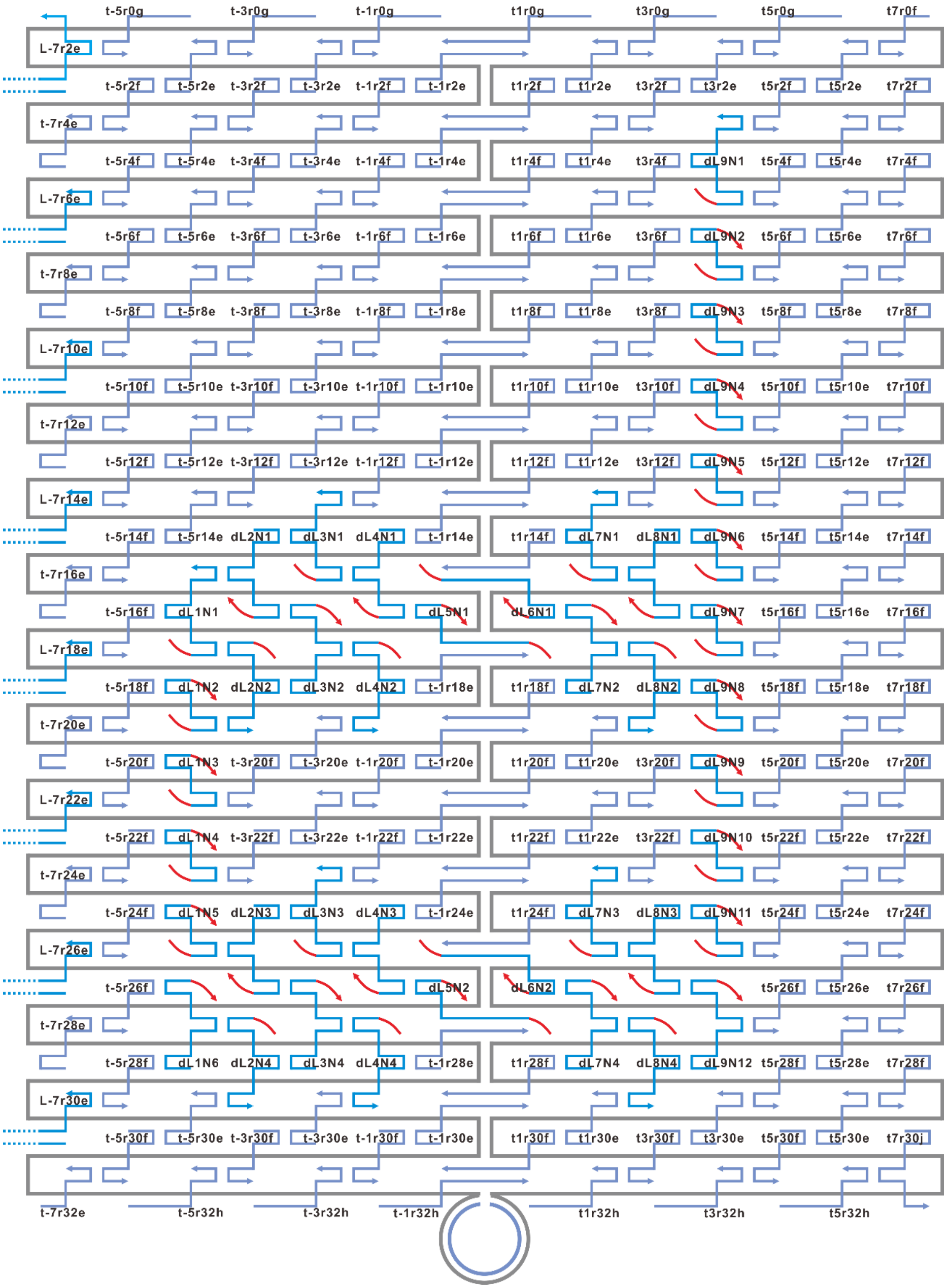

Supplementary Figure 44. Schematic of pcDNA on alphabet 'd' pattern of tall rectangular DNA origami template.

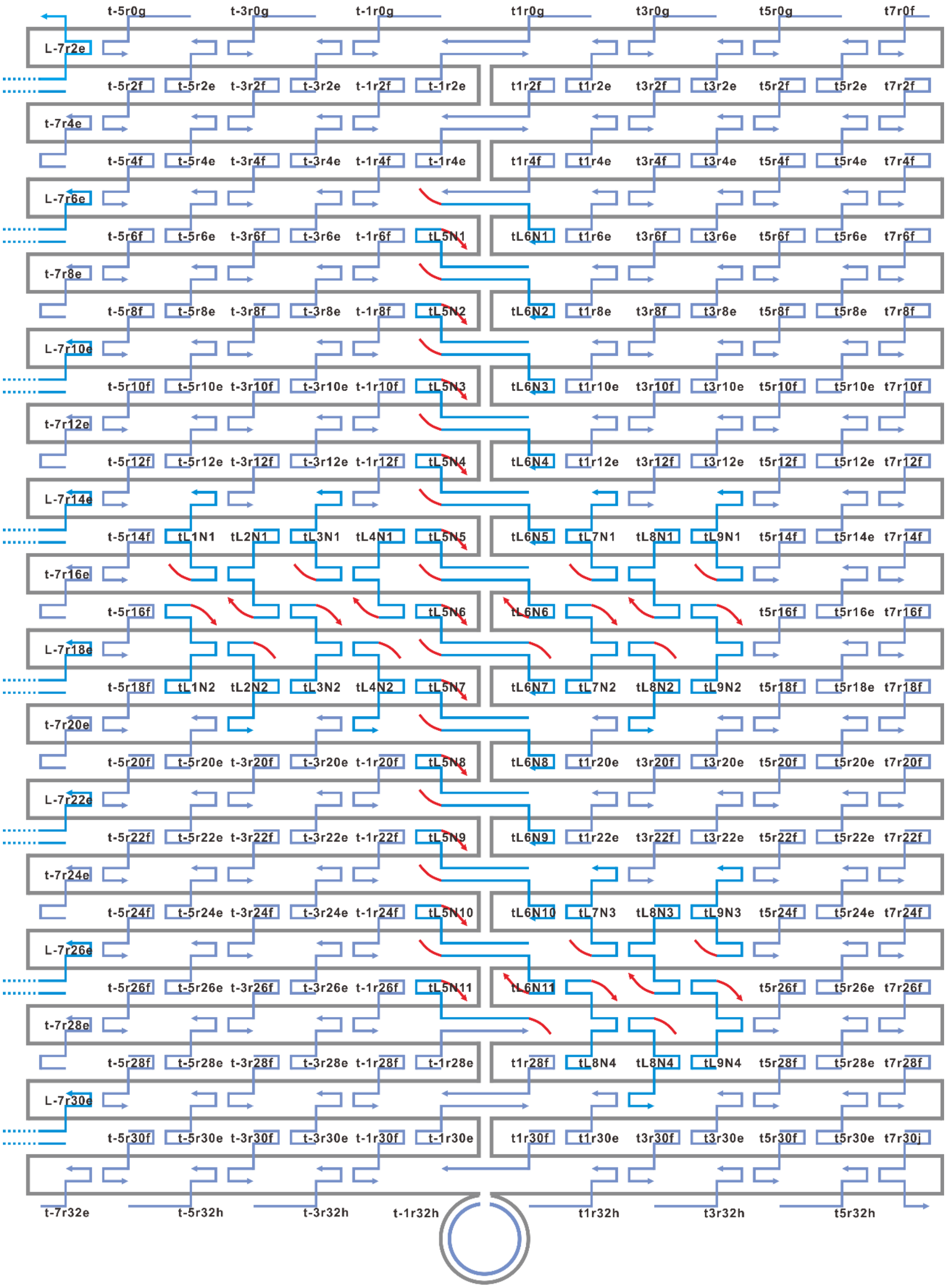

Supplementary Figure 45. Schematic of pcDNA on alphabet ‘t’ pattern of tall rectangular DNA origami template.

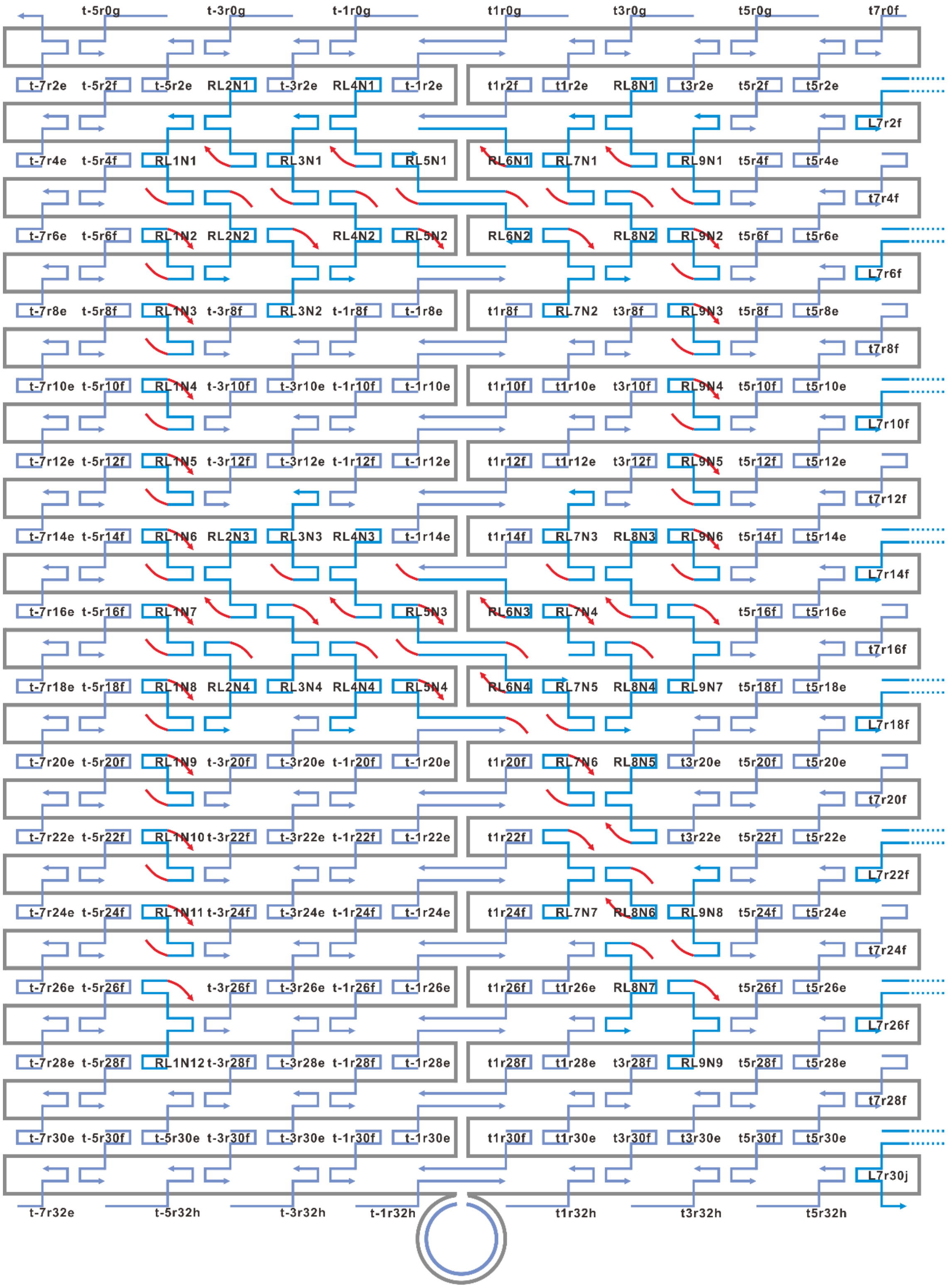

Supplementary Figure 46. Schematic of pcDNA on alphabet ‘R’ pattern of tall rectangular DNA origami template.

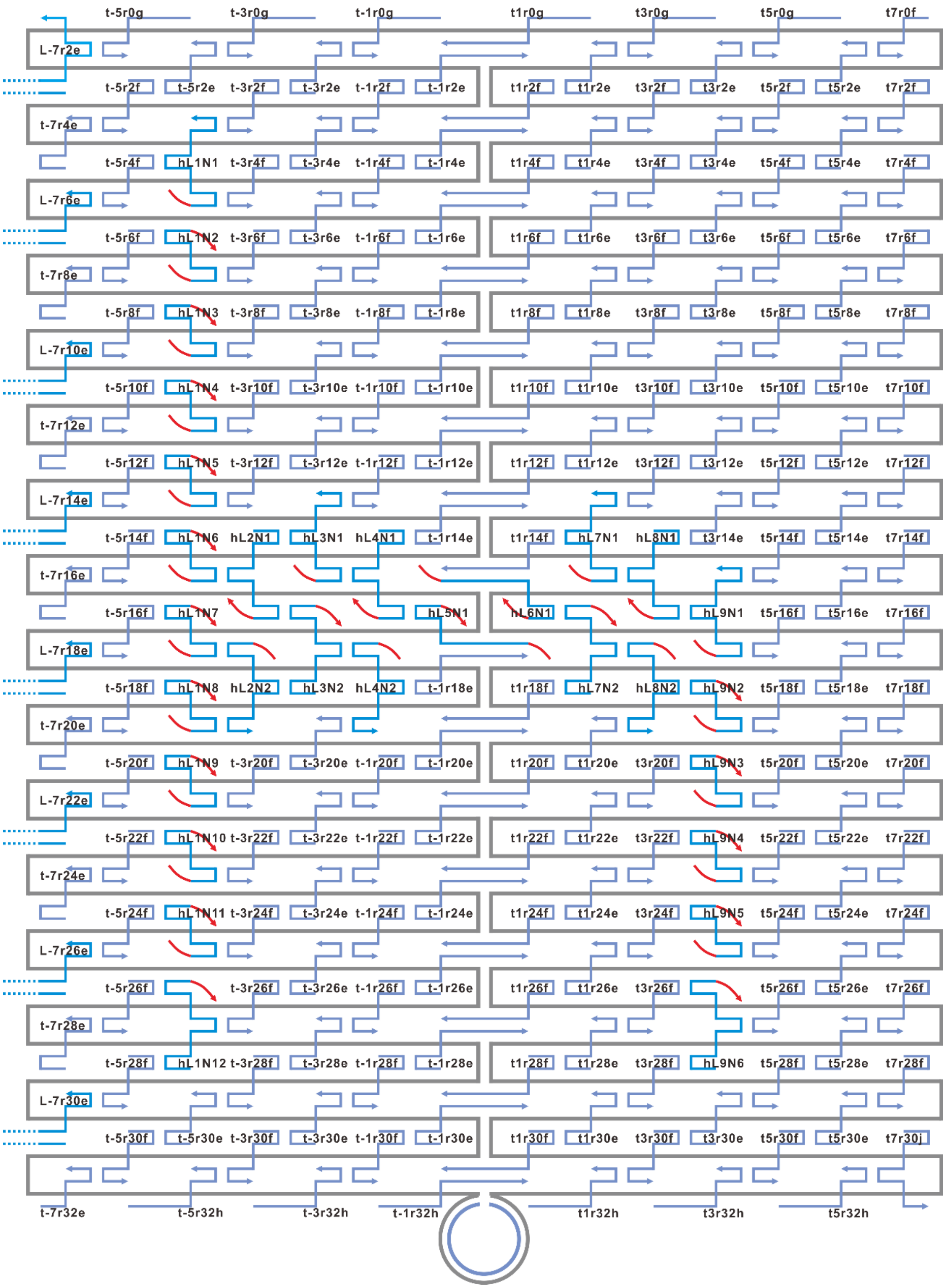

Supplementary Figure 47. Schematic of pcDNA on alphabet 'h' pattern of tall rectangular DNA origami template.

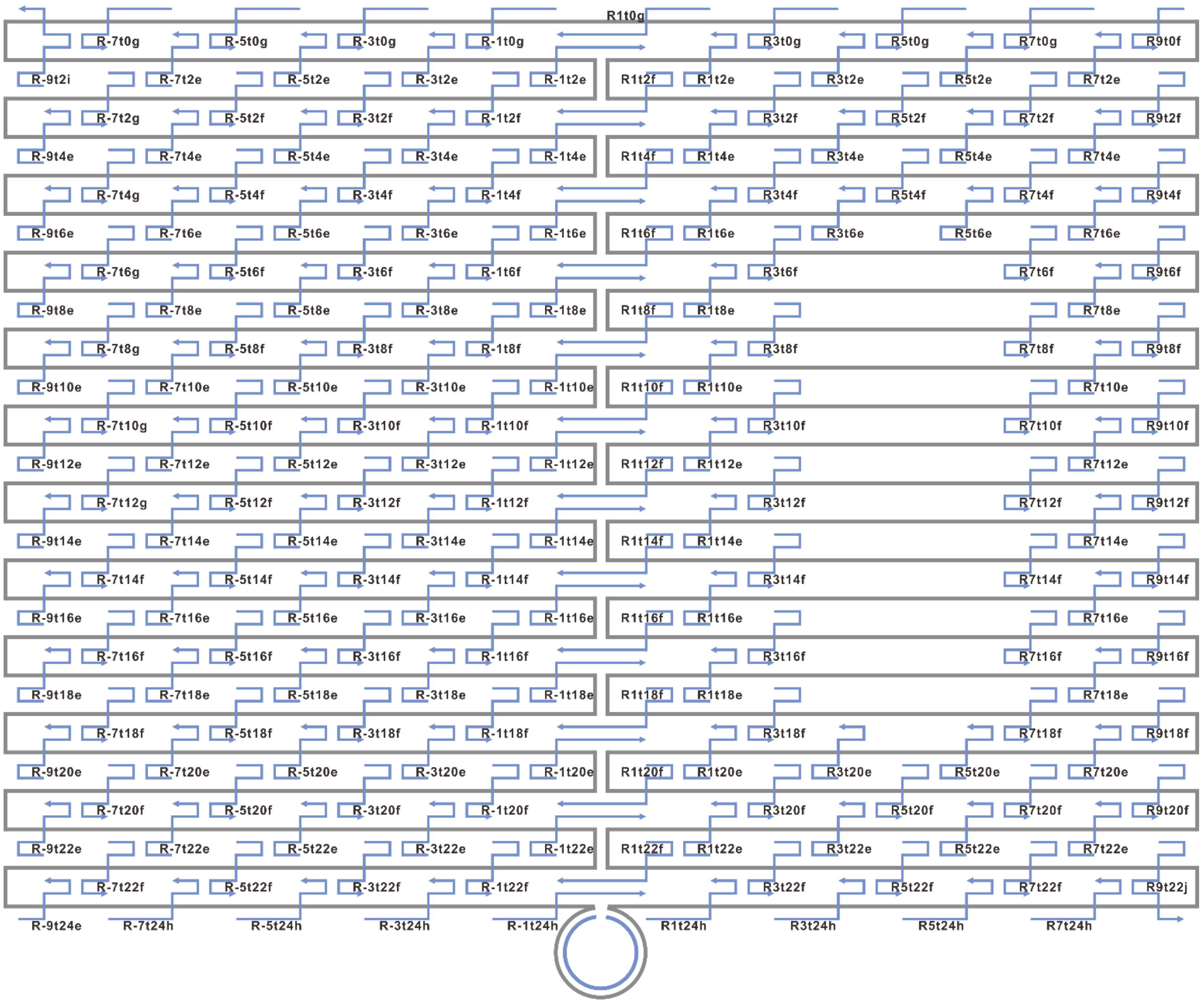

Supplementary Figure 48. Schematic of rectangular hole on rectangular DNA origami template.

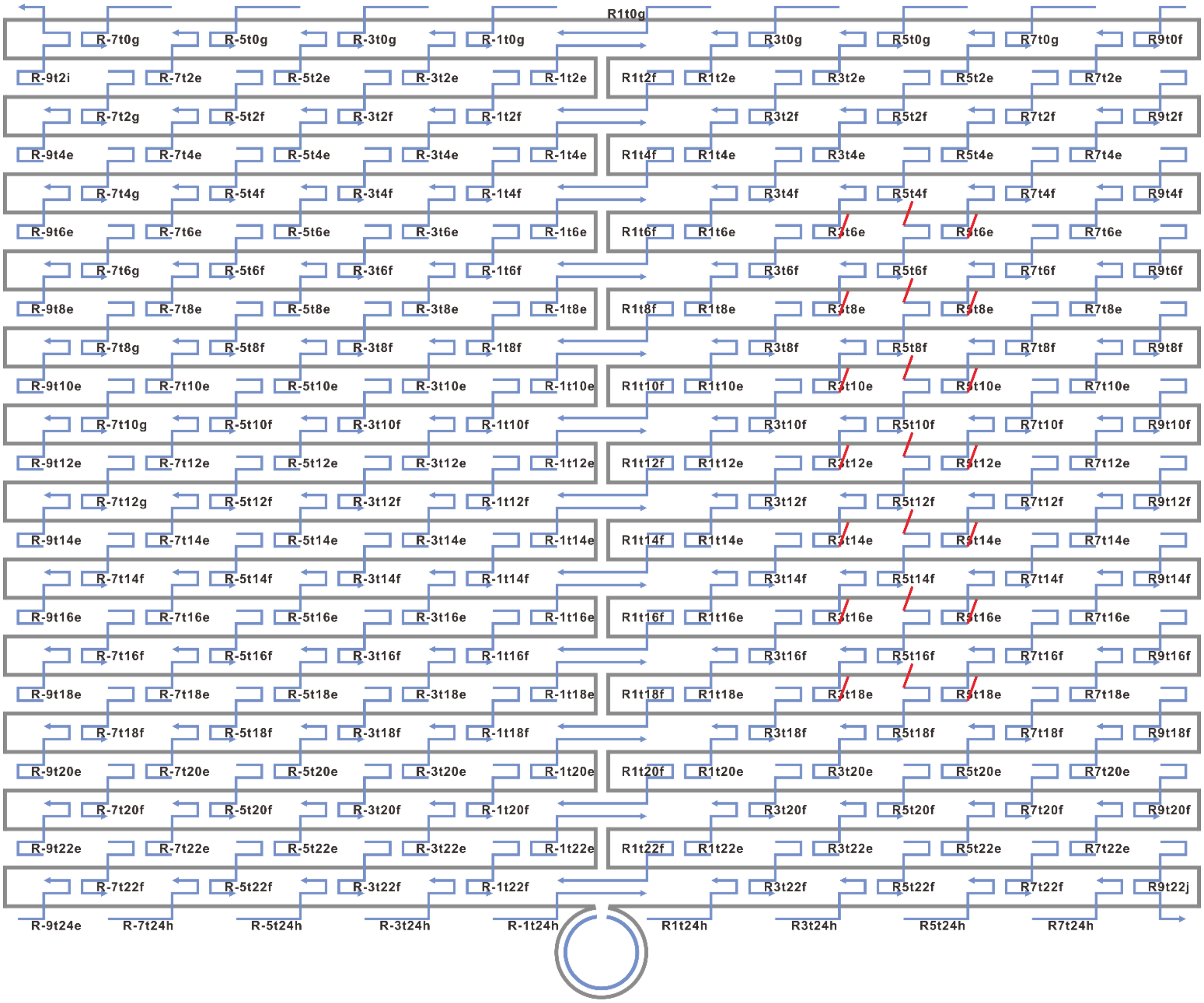

Supplementary Figure 49. Schematic of rectangular pattern on rectangular DNA origami template.

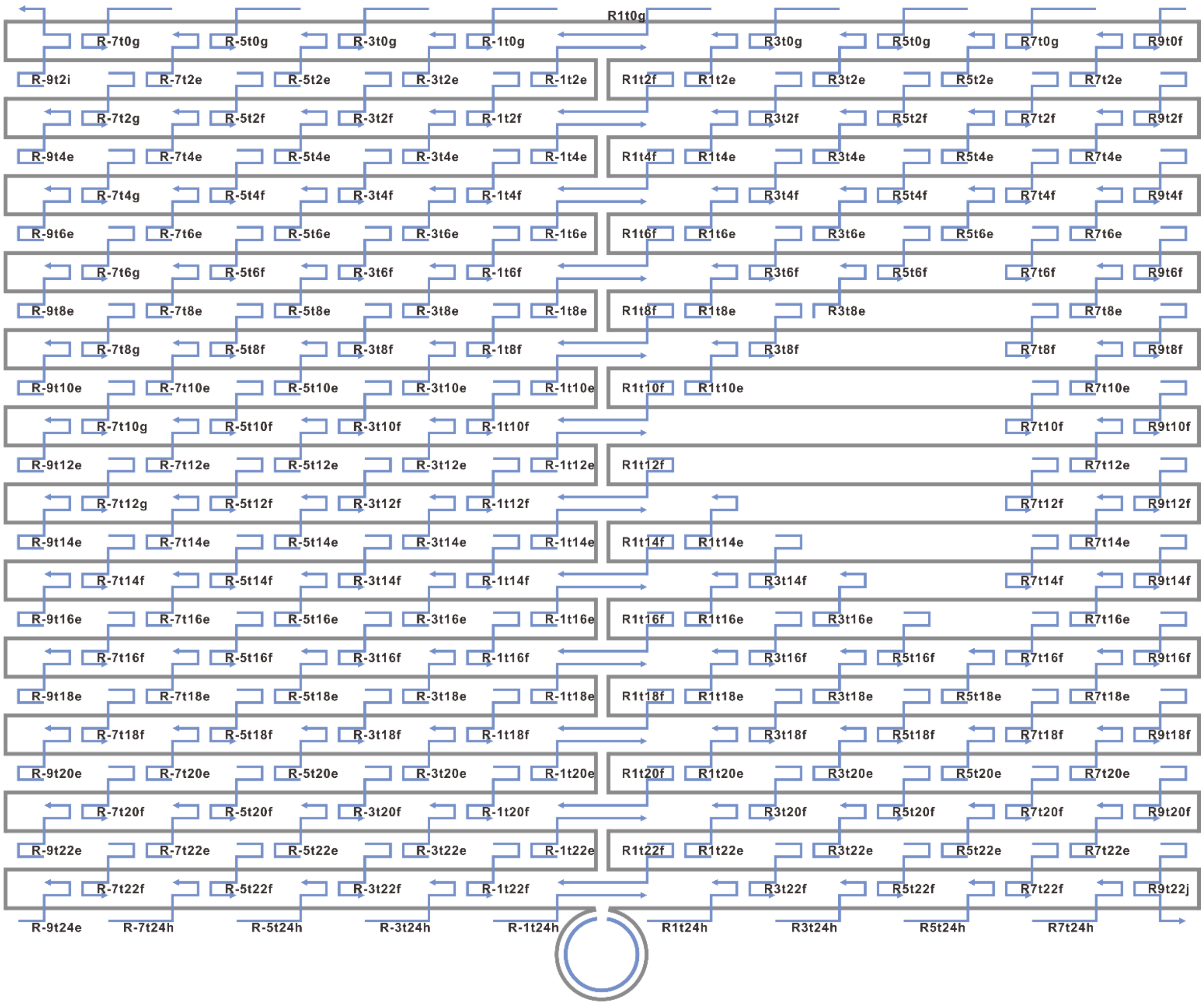

Supplementary Figure 50. Schematic of triangular hole on rectangular DNA origami template.

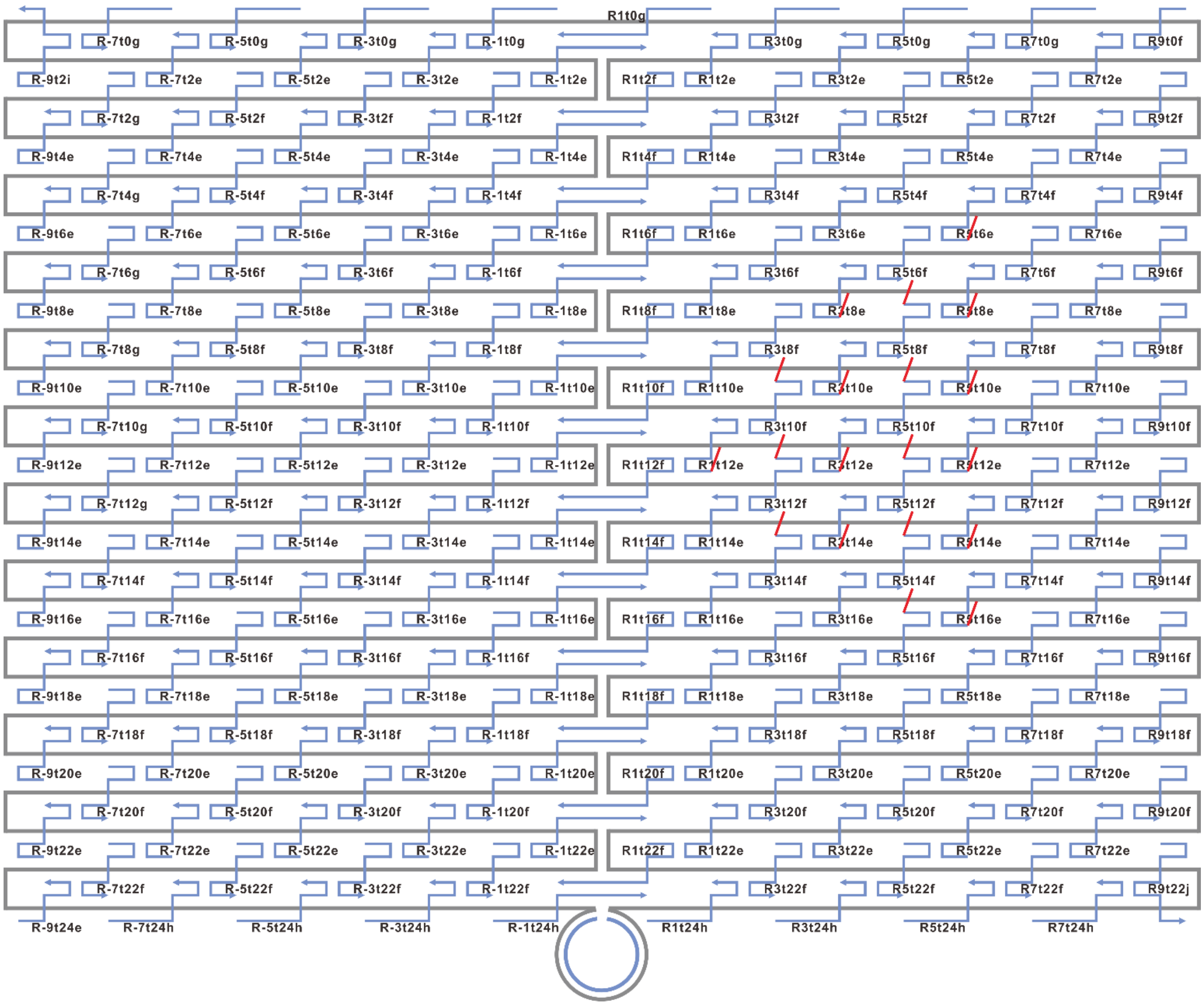

Supplementary Figure 51. Schematic of triangular pattern on rectangular DNA origami template.

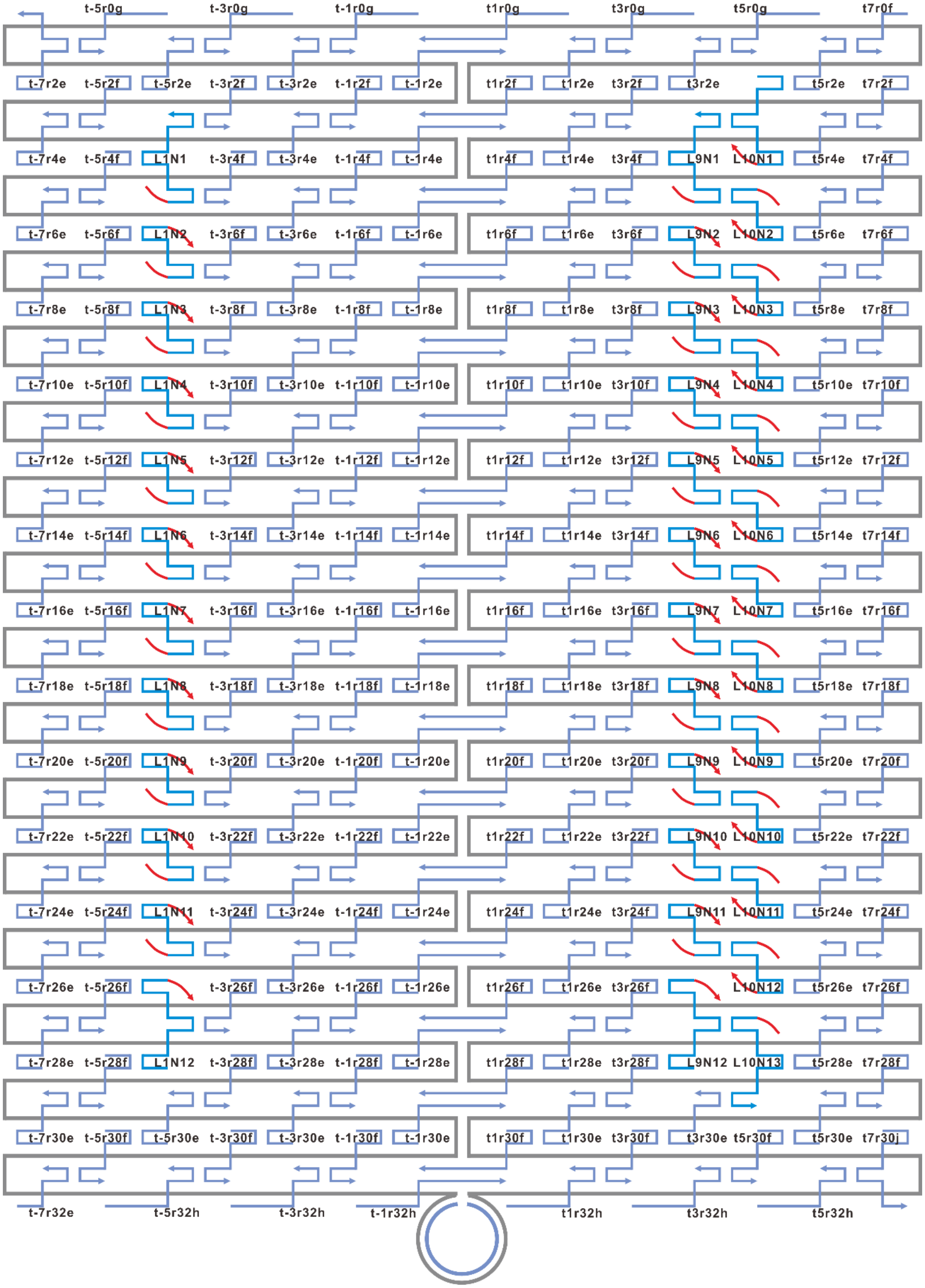

Supplementary Figure 52. Schematic of single and double lines pcDNA of tall rectangular DNA origami template.

Supplementary Notes:

DNA sequences:

For two sides pattern of triangular DNA origami:

The sequence of pcDNA was AGACTAGACTAGACT-5' linked to all the selected staple strands as shown in Figure. S11.

Other sequences can be found in the sequences list for the triangle origami[1].

For digit 8 pattern of rectangular DNA origami:

RL1N1: CTTGAGAGAGCGACGAGTCTGGAAAACTAGCATGTCAAGATTCTCCGTGGGAACCGTTGGTG

RL1N2: ACTTGAGAGAGCGACAGCCTCAGTTATGACCCTGTAATATTGCCTGAGACCTGACGACATAG

RL1N3: ACTTGAGAGAGCGACAGAGCTTATTTAAATATGCAACTAAGCAATAAGACCTGACGACATAG

RL1N4: ACTTGAGAGAGCGACTACTGCGGAATGCTTTAAACAGTTGATGGCTTGACCTGACGACATAG

RL1N5: ACTTGAGAGAGCGACCTCATTATTTAATAAAACGAACTAGCGTCCAAGACCTGACGACATAG

RL1N6: CGGCTACTTACTTAGCCGGAACGCTGACCAACTTTGAAAAGAACTGGGACCTGACGACATAG

RL2N1: GCCAGCTGCCTGCAGGTCGACTCTGCAAGGCGATTAAGTTCGCATCGTGACCTGACGACATAG

RL2N2: ACTTGAGAGAGCGACAACCGTGCGAGTAACAACCCGTCGTCATATGTGACCTGACGACATAG

RL2N3: ACTTGAGAGAGCGACACCCCGGTAAAGGCTATCAGGTCACTTTTGCGGACCTGACGACATAG

RL2N4: ACTTGAGAGAGCGACGGAGAAGCAGAATTAGCAAAATTAAAGTACGGGACCTGACGACATAG

RL2N5: ACTTGAGAGAGCGACTGTCTGGAAGAGGTCATTTTTGCGCAGAAAACGACCTGACGACATAG

RL2N6: ACTTGAGAGAGCGACGAGAATGAATGTTTAGACTGGATAACGGAACAGACCTGACGACATAG

RL2N7: ACTTGAGAGAGCGACACATTATTACCTTATGCGATTTTAGAGGACAGGACCTGACGACATAG

RL2N8: ACTTGAGAGAGCGACATGAACGGCGCGACCTGCTCCATGAGAGGCTTGACCTGACGACATAG

RL2N9: ACTTGAGAGAGCGACTGAGGACTAGGGAGTTAAAGGCCGAAAGGAACAACTAAAGCTTTCCAG

RL3N1: ACTTGAGAGAGCGACGATGTGCTAGAGGATCCCCGGGTACTTTCCAGTCGGGAAACGGGCAAC

RL3N2: ACTTGAGAGAGCGACATGTGAGCATCTGCCAGTTTGAGGGAAAGGGGGACCTGACGACATAG

RL3N3: CAACGCAATTTTTGAGAGATCTACTGATAATC AGAAAAGCAACATTAAGACCTGACGACATAG

RL3N4: ACTTGAGAGAGCGAC TCGAAATCTGTACAGA CCAGGCGCTTAATCATTGTGAATTACAGGTAG

RL3N5: ACTTGAGAGAGCGAC AGGCTTGCAAAGACTT TTTCATGAAAATTGTGGACCTGACGACATAG

RL3N6: CGTAACGATCTAAAGTTTTGTCGTGAATTGCG AATAATAAGGTCGCTGGACCTGACGACATAG

RL4N1: ACTGCCCGCCGAGCTCGAATTCGTTATTACGCCAGCTGGCGGACGACGGACCTGACGACATAG

RL4N2: ACTTGAGAGAGCGACACAGTATCGTAGCCAGCTTTCATCCCCAAAAACAGGAAGACCGGAGAG

RL4N3: TTTCAACTATAGGCTGGCTGACCTTGTATCATCGCCTGATGGAAGTTTGACCTGACGACATAG

RL4N4: ACTTGAGAGAGCGACCCATTAAACATAACCGATATATTCTTTTTTCACGTTGAAAATAGTTAG

RL5N1: ACTTGAGAGAGCGACCTCTTCGCAATCATGGTCATAGCTACTCACATTAATTGCGCCCTGAGA

RL5N2: ACTTGAGAGAGCGACGCCTTCCTGGCCTCAGGAAGATCGGTGCGGGCGACCTGACGACATAG

RL5N3: TATATTTTAGCTGATAAATTAATGTTGTATAAGCAAATATCGCGTCTGGACCTGACGACATAG

RL5N4: ACTTGAGAGAGCGACCGGAGATTTCATCAAGAGTAATCTTAAATTGGGCTTGAGAGAATACCA

RL5N5: ACTTGAGAGAGCGACCGCCCACGCGGGTAAAATACGTAAAAGTACAAGACCTGACGACATAG

RL5N6: TGTAGCATTCCACAGACAGCCCTCATCTCCAA AAAAAAGGACAACCATGACCTGACGACATAG

RL6N1: GTGAGCTAGTTTCCTGTGTGAAATTTGGGAAG GGCGATCGCACTCCAGGACCTGACGACATAG

RL6N2: ACTTGAGAGAGCGACCCAGCTTTGCCATCAA AAATAATTTTAAATTGTAAACGTTGATATTCA

RL6N3: ACGAGTAGTGACAAGAACCGGATATACCAAGCGCGAAACATGCCACTAGACCTGACGACATAG

RL6N4: ACTTGAGAGAGCGACCGAAGGCATGCGCCGACAATGACACTCCAAAAGGAGCCTTACAACGCC

RL7N1: ACTTGAGAGAGCGACCGCAACTGTGTTATCCGCTCACAATGTAAAGCCTGGGGTGGGTTTGCC

RL7N2: ACTTGAGAGAGCGACATAGGAACCCGGCACCGCTTCTGGTCAGGCTGGACCTGACGACATAG

RL7N3: AGGTAAAGAAATCACCATCAATATAATATTTTGTTAAAATTTTAACCAGACCTGACGACATAG

RL7N4: ACTTGAGAGAGCGACAGCGATTATTCATTACCCAAATCACTTGCCCTGACGAGAACGCCAAAA

RL7N5: ACTTGAGAGAGCGACCCGATAGTCCAACCTAAAACGAAATGACCCCCGACCTGACGACATAG

RL7N6: TGAGTTTCGTCACCAGTACAAACTTAATTGTA TCGGTTTAGCTTGATAGACCTGACGACATAG

RL8N1: ACTTGAGAGAGCGACTATACAGTAAGCGCCATTCGCCATTGCCGGAAGACCTGACGACATAG

RL8N2: ACTTGAGAGAGCGACATAGCGATTTAAATCAGCTCATTTTCGCATTAGACCTGACGACATAG

RL8N3: ACTTGAGAGAGCGACCTGTTTAGAAGGCCGGAGACAGTCATTCAAAAGACCTGACGACATAG

RL8N4: ACTTGAGAGAGCGACTAGAAACCAGCTATATTTTCATTTGGTCAATAGACCTGACGACATAG

RL8N5: ACTTGAGAGAGCGACGAGCGTCTATATCGCGTTTTAATTGCCCGAAAGACCTGACGACATAG

RL8N6: ACTTGAGAGAGCGACCGAAGCCCAACACTATCATAACCCGAGGCATAGACCTGACGACATAG

RL8N7: ACTTGAGAGAGCGACTAAATATTCATTCAGTGAATAAGGACGTAACAGACCTGACGACATAG

RL8N8: ACTTGAGAGAGCGACGGAACCGCCTAAAACACTCATCTTGAGGCAAAGACCTGACGACATAG

RL8N9: ACTTGAGAGAGCGACGCCCGTATGTGAATTTCTTAAACATCAGCTTG

RL9N1: ACTTGAGAGAGCGACACCAGGCAAACAGTACCTTTTACACAGATGAAGACCTGACGACATAG

RL9N2: ACTTGAGAGAGCGACAATTTTTGAGCTTAGATTAAGACGTTGAAAACGACCTGACGACATAG

RL9N3: ACTTGAGAGAGCGACGGGTGAGATATCATATGCGTTATAGAAAAAGCGACCTGACGACATAG

RL9N4: ACTTGAGAGAGCGACACCTGTTTAATCAATAATCGGCTGCGAGCATGGACCTGACGACATAG

RL9N5: ACTTGAGAGAGCGACGACTTCAATTCCAGAGCCTAATTTACGCTAACGACCTGACGACATAG

RL9N6: ACTTGAGAGAGCGACGTAAGAGCTTTTTAAGAAAAGTAATATCTTACGACCTGACGACATAG

RL9N7: ACTTGAGAGAGCGACAAGCTGCTGACGGAAATTATTCATAGGGAAGGGACCTGACGACATAG

RL9N8: ACTTGAGAGAGCGACAGAATACACTCCCTCAGAGCCGCCCCACCACCGACCTGACGACATAG

RL9N9: CTTTCGAGAAACAGTTAATGCCCCGTAACAGTGACCTGACGACATAG

RL10N1: GGATTTAGCGTATTAAATCCTTTGTTTTCAGGTTTAACGTTCGGGAGAGACCTGACGACATAG

RL10N2: ACTTGAGAGAGCGACAACAATAATTTTCCCTTAGAATCCCTGAGAAGAGTCAATAGGAATCAT

RL10N3: ATTGAGGGTAAAGGTGAATTATCAATCACCGGAACCAGAGACCCTCAGGACCTGACGACATAG

RL10N4: ACTTGAGAGAGCGACAACCGCCAGGGGTCAGTGCCTTGACTGCCTATTTCGGAACAGGGATAG

RL11N1: ACTTGAGAGAGCGACTGCGTAGACCCGAACGTTATTAATGCCGTCAATAGATAATCAGAGGTG

RL11N2: ACTTGAGAGAGCGACTTAATTAACGGATTCGCCTGATTGAAAGAAATGACCTGACGACATAG

RL11N3: ACGCTCAAAATAAGAATAAACACCGTGAATTTATCAAAATCGTCGCTAGACCTGACGACATAG

RL11N4: ACTTGAGAGAGCGACTAATCAAACCGTCACCGACTTGAGAGACAAAAGGGCGACAAGTTACCA

RL11N5: ACTTGAGAGAGCGACGTTTTAACCCCTCAGAGCCACCACTCTTTTCAGACCTGACGACATAG

RL11N6: CTCAGAGCCACCACCCTCATTTTCCTATTATTCTGAAACAGGTAATAAGACCTGACGACATAG

RL12N1: AGATTAGATTTAAAAGTTTGAGTACACGTAAAACAGAAATCTTTGAATGACCTGACGACATAG

RL12N2: ACTTGAGAGAGCGACACCAAGTTCCTTGCTTCTGTAAATCATAGGTCTGAGAGACGATAAATA

RL12N3: AGCGCCAACCATTTGGGAATTAGATTATTAGCGTTTGCCACCTCAGAGGACCTGACGACATAG

RL12N4: ACTTGAGAGAGCGACCCGCCACCGATACAGGAGTGTACTTGAAAGTATTAAGAGGCCGCCACC

RL13N1: ACTTGAGAGAGCGACATTATTTGACATTATCATTTTGCGTCTTTAGGAGCACTAAGCAACAGT

RL13N2: ACTTGAGAGAGCGACGTGAATAAACAAAATCGCGCAGAGATATCAAAGACCTGACGACATAG

RL13N3: CATATTTAGAAATACCGACCGTGTTACCTTTTTAACCTCCATATGTGAGACCTGACGACATAG

RL13N4: ACTTGAGAGAGCGACTAGCCCCCGCCAGCAAAATCACCAAATAGAAAATTCATATATAACGGA

RL13N5: ACTTGAGAGAGCGACCTTTTGATAGAACCACCACCAGAGTTCGGTCATCTAAAGACGTTGGG

RL13N6: CCCTCAGAACCGCCACCCTCAGAACTGAGACTCCTCAAGAATACATGGGACCTGACGACATAG

RL14N1: CTAAAATAGAACAAAGAAACCACCAGGGTTAGAACCTACCGCGAATTAGACCTGACGACATAG

RL14N2: ACTTGAGAGAGCGACTTCATTTCCAGTACATAAATCAATGGCTTAGGTTGGGTTACTAAATTT

RL14N3: TCACAATCGTAGCACCATTACCATCGTTTTCATCGGCATTCCGCCGCCGACCTGACGACATAG

RL14N4: ACTTGAGAGAGCGACAGCATTGACGTTCCAGTAAGCGTCGAAGGATTAGGATTAGTACCGCCA

RL15N1: ACTTGAGAGAGCGACATAATGGAAGAAGGAGCGGAATTATTGAAAGGAATTGAGGTGAAAAAT

RL15N2: ACTTGAGAGAGCGACAATGGAAAAATTACCTGAGCAAAAACTTCTGAGACCTGACGACATAG

RL15N3: ACTTGAGAGAGCGACCTTCTGACTATAACTATATGTAAACCTTTTTTGACCTGACGACATAG

RL15N4: ACTTGAGAGAGCGACAACAACATATTTAGGCAGAGGCATAATTTCATGACCTGACGACATAG

RL15N5: ACTTGAGAGAGCGACCCTCCCGACGTAGGAATCATTACCCGACAATAGACCTGACGACATAG

RL15N6: ACTTGAGAGAGCGACGTAATTGAATAGCAGCCTTTACAGTTAGCGAAGACCTGACGACATAG

RL15N7: ACTTGAGAGAGCGACGAATAAGTAAGACTCCTTATTACGGTCAGAGGGACCTGACGACATAG

RL15N8: ACTTGAGAGAGCGACCTGTAGCGTAGCAAGGCCGGAAACACACCACGGACCTGACGACATAG

RL15N9: ACTTGAGAGAGCGACGAATTTACCAGGAGGTTGAGGCAGGCGTCAGAGACCTGACGACATAG

RL15N10: TATCACCGTACTCAGGAGGTTTAGCGGGGTTTTGCTCAGTCAGTCTCTGACCTGACGACATAG

RL16N1: TGGATTATGAAGATGATGAAACAAAATTTCATTTGAATTATGCTGATGGACCTGACGACATAG

RL16N2: ACTTGAGAGAGCGACCAAATCCACAAATATATTTTAGTTTTTCGAGCGACCTGACGACATAG

RL16N3: ACTTGAGAGAGCGACCAGTAATAAATTCTGTCCAGACGAGCGCCCAAGACCTGACGACATAG

RL16N4: ACTTGAGAGAGCGACTAGCAAGCAAGAACGCGAGGCGTTAGAGAATAGACCTGACGACATAG

RL16N5: ACTTGAGAGAGCGACACATAAAAGAACACCCTGAACAAACAGTATGTGACCTGACGACATAG

RL16N6: ACTTGAGAGAGCGACTAGCAAACTAAAAGAAACGCAAAGGTCACCAAGACCTGACGACATAG

RL16N7: ACTTGAGAGAGCGACTGAAACCAATCAAGTTTGCCTTTAGTCAGACGATTGGCCTGCCAGAAT

Other sequences can be found in the sequences list for the rectangle origami[2].

For alphabet 'A', 'g', 'P', 'd', 't', 'C', 'o', 'R', 'h', 'N' and 'i' pattern of tall rectangular DNA origami:

gL1N1, PL1N1, CL1N1, RL1N1, hL1N1, NL1N1:

TCGACGACTACTGACTGAGGACTAGGGAGTTAAAGGCCGCTCCAAAAGGAGCCTTAGCGGAGT

gL1N2, PL1N2, CL1N2, RL1N2, hL1N2, NL1N2:

TCGACGACTACTGACCCGGAACGTACCAAGCGCGAAACAAGAGGCTTCTTGGGTCGTAGACA

gL1N3, PL1N3, CL1N3, RL1N3, hL1N3, NL1N3:

TCGACGACTACTGACGCTTGAGATTCATTACCCAAATCATTACTTAGCTTGGGTCGTAGACA

gL1N4, PL1N4, CL1N4, RL1N4, hL1N4, NL1N4:

TCGACGACTACTGACGTAAGAGCACAGGTAGAAAGATTCTAAATTGGCTTGGGTCGTAGACA

gL1N5, PL1N5, CL1N5, RL1N5, hL1N5, NL1N5:

TCGACGACTACTGACACCCTGACAATCGTCATAAATATTGAGGCATACTTGGGTCGTAGACA

gL1N6, PL1N6, CL1N6, RL1N6, hL1N6, NL1N6:

TCGACGACTACTGACCAACATGTTTAGAGAGTACCTTTAAGGTCTTTCTTGGGTCGTAGACA

tL1N1: TCGACGACTACTGACCAACATGTTTAGAGAGTACCTTTAAGGTCTTTACCCTGACAATCGTCA

gL1N7, tL1N2: TATATTTTCATACAGGCAAGGCAAAGCTATATTTTCATTTCTGTAGCTCTTGGGTCGTAGACA

tL1N2, oL1N1: TCGACGACTACTGACCAAGGCAAAGCTATATTTTCATTTCTGTAGCTCAACATGTTTAGAGAG

PL1N7, CL1N7, RL1N7, hL1N7, NL1N7:

TCGACGACTACTGACCAAGGCAAAGCTATATTTTCATTTCTGTAGCTCTTGGGTCGTAGACA

PL1N8, dL1N2, CL1N8, oL1N2, RL1N8, hL1N8, NL1N8:

TCGACGACTACTGACATCAATATAACCCTCATATATTTTCATACAGGCTTGGGTCGTAGACA

PL1N9, dL1N3, CL1N9, oL1N3, RL1N9, hL1N9, NL1N9:

TCGACGACTACTGACTAAACGTTAAAACTAGCATGTCAAAAATCACCCTTGGGTCGTAGACA

PL1N10, dL1N4, CL1N10, oL1N4, RL1N10, hL1N10, NL1N10:

TCGACGACTACTGACAACCGTGCGAGTAACAACCCGTCGTTAAATTGCTTGGGTCGTAGACA

PL1N11, dL1N5, CL1N11, oL1N5, RL1N11, hL1N11, NL1N11:

TCGACGACTACTGACCCAGGGTTTTGGGAAGGGCGATCGCGCATCGTCTTGGGTCGTAGACA

AL1N1, gL1N8: TCGACGACTACTGACCCAGGGTTTTGGGAAGGGCGATCGCGCATCGTAACCGTGCGAGTAACA

AL1N12, gL1N9, PL1N12, dL1N6, CL1N12, oL1N6, RL1N12, hL1N12, NL1N12:

AGCTGATTACTCACATTAATTGCGTGTTATCCGCTCACAAGGGTAACGCTTGGGTCGTAGACA

gL2N1, PL1N1, CL2N1, RL2N1:

TGTAGCATAACTTTCAACAGTTTCTAATTGTATCGGTTTAGGTCGCTGCTTGGGTCGTAGACA

gL2N2, PL2N2, CL2N2, RL2N2:

TCGACGACTACTGACAGGCTTGCAAAGACTTTTTCATGATGACCCCCAGCGATTAAGGCGCAG

NL2N1: TCGGTTTAGGTCGCTGAGGCTTGCAAAGACTTTTTCATGATGACCCCCCTTGGGTCGTAGACA

NL2N2: TCGACGACTACTGACAGCGATTAAGGCGCAGACGGTCAATGACAAGACTTGGGTCGTAGACA

NL2N3: TCGACGACTACTGACACCGGATATGGTTTAATTTCAACTACGGAACAACATTATTAACACTAT

gL2N3, PL2N3, dL2N1, tL2N1, oL2N1, RL2N3, hL2N1:

TCAGAAGCCTCCAACAGGTCAGGATTTAAATATGCAACTAGGTCAATACTTGGGTCGTAGACA

gL2N4, PL2N4, dL2N2, tL2N2, oL2N2, RL2N4, hL2N2:

TCGACGACTACTGACACCTGTTTAGAATTAGCAAAATTAGGATAAAAATTTTTAGGATATTCA

gL2N5, dL2N3, CL2N5, oL2N3:

GTTTGAGGTCAGGCTGCGCAACTG TTCCCAGTCACGACGTGTTTCCTGCTTGGGTCGTAGACA

gL2N6, dL2N4, CL2N6, oL2N4:

TCGACGACTACTGACTGTGAAATTTGCGCTCACTGCCCGCTTTTCACCAGTGAGATGGTGGTT

gL3N1, PL3N1, CL3N1, RL3N1:

TCGACGACTACTGACCCATTAAACATAACCGATATATTCTCAGCTTGCTTTCGAGTGGGATTT

gL3N2, PL3N2, CL3N2, RL3N2:

AGTAATCTTCATAAGGGAACCGAACTAAAACACTCATCTT GGAAGTTTCTTGGGTCGTAGACA

NL3N1: TCGACGACTACTGACTGTGAATTTCATCAAGAGTAATCTTCATAAGGGAACCGAACTAAAACA

NL3N2: TCGACGACTACTGACCAGACGACTTAATAAAACGAACTATTAATCATCTTGGGTCGTAGACA

NL3N3: GAAGCAAAAAAGCGGATTGCATCAATGTTTAGACTGGATATCGTTTACCTTGGGTCGTAGACA

gL3N3, PL3N3, dL3N1, tL3N1, oL3N1, RL3N3, hL3N1:

TCGACGACTACTGACTGTCTGGACCAGACCGGAAGCAAAAAAGCGGATTGCATCAATGTTTAG

gL3N4, PL3N4, dL3N2, tL3N2, oL3N2, RL3N4, hL3N2:

CAACGCAAAGCAATAAAGCCTCAGGATACATTTCGCAAATAAGTACGGCTTGGGTCGTAGACA

gL3N5, dL3N3, CL3N3, oL3N3:

TCGACGACTACTGACGACGGCCAAAGCGCCATTCGCCATGGACGACGACAGTATCGTAGCCAG

gL3N6, dL3N4, CL3N4, oL3N4:

TGGTTTTTCTTTCCAGTCGGGAAAAATCATGGTCATAGCTTGTAAAACCTTGGGTCGTAGACA

gL4N1, PL4N1, CL4N1, RL4N1:

CGTAACGAAAATGAATTTTCTGTAGTGAATTTCTTAAACAACAACCATCTTGGGTCGTAGACA

gL4N2, PL4N2, CL4N2, RL4N2:

TCGACGACTACTGACCGCCCACGCGGGTAAAATACGTAAGAGGCAAAAGAATACACTGACCAA

NL4N1: CGATTTTAGGAAGAAAAATCTACGGATAAAAACCAAAATAAGGGGGTACTTGGGTCGTAGACA

NL4N2: TCGACGACTACTGACATAGTAAAAAAAGATTAAGAGGAACGAGCTTCCTTGGGTCGTAGACA

NL4N3: TCGACGACTACTGACAAAGCGAAAGTTTCATTCCATATATTTAGTTTGACCATTAAGCATAAA

gL4N3, PL4N3, dL4N1, tL4N1, oL4N1, RL4N3, hL4N1:

AAGAGGAACGAGCTTCAAAGCGAAAGTTTCATTCCATATATTTAGTTTCTTGGGTCGTAGACA

gL4N4, PL4N4, dL4N2, tL4N2, oL4N2, RL4N4, hL4N2:

TCGACGACTACTGACGACCATTAAGCATAAAGCTAAATCCTTTTGCGGGAGAAGCCCGGAGAG

gL4N5, dL4N3, CL4N3, oL4N3:

GAAGATCGTGCCGGAAACCAGGCAGTGCCAAGCTTGCATGCCGAGCTCCTTGGGTCGTAGACA

gL4N6, dL4N4, CL4N4, oL4N4:

TCGACGACTACTGACGAATTCGTCCTGTCGTGCCAGCTGCGGTTTGCGTATTGGGAATCAAAA

gL5N1, PL5N1, CL5N1, RL5N1: TCGACGACTACTGACGGAACCGCTGCGCCGACAATGACAGCTTGATA

gL5N2, PL5N2, tL5N1, CL5N2, RL5N2, iL1N1:

GAATTAGACCAACCTAAAACGAAATGCCACTACTTGGGTCGTAGACA

tL5N2: TAGCAAACTGTACAGACCAGGCGCGAGGACAGCTTGGGTCGTAGACA

tL5N3: GTAATTGAACCAGTCAGGACGTTGAGAACTGGCTTGGGTCGTAGACA

tL5N4, iL1N2: ACAATTTTAAAGAAGTTTTGCCAGGCGAGAGGCTTGGGTCGTAGACA

tL5N5, NL5N1, iL1N3: TATCATTCATATCGCGTTTTAATTGCCCGAAACTTGGGTCGTAGACA

gL5N3, PL5N3, dL5N1, tL5N6, oL5N1, RL5N3, hL5N1, NL5N2:

TCGACGACTACTGACAACATGTATCTGCGAACGAGTAGAACAGTTGACTTGGGTCGTAGACA

iL1N4: AACATGTATCTGCGAACGAGTAGAACAGTTGACTTGGGTCGTAGACA

tL5N7, iL1N5: AACTTTTTTTATGACCCTGTAATAGGTTGTACCTTGGGTCGTAGACA

RL5N4, NL5N3: TCGACGACTACTGACAACTTTTTTTATGACCCTGTAATA GGTTGTACCTTGGGTCGTAGACA

tL5N8, iL1N6: GTGAATAAAAAGGCTATCAGGTCATTTTTGAGCTTGGGTCGTAGACA

tL5N9, iL1N7: TATACAGTGCCATCAAAAATAATTTTTAACCACTTGGGTCGTAGACA

tL5N10, iL1N8: TTTGAGTACCGGCACCGCTTCTGGCACTCCAGCTTGGGTCGTAGACA

gL5N4, dL5N2, tL5N11, CL5N3, oL5N2:

TCGACGACTACTGACCTGAACCTAGAGGATCCCCGGGTACCTGCAGGCTTGGGTCGTAGACA

iL1N9: TCGACTCTCAAATATCCCCGGGTACCTGCAGGCTTGGGTCGTAGACA

gL6N1, PL6N1, CL6N1, RL6N1: CCGATAGTCTCCCTCAGAGCCGCCCCACCACCCTTGGGTCGTAGACA

gL6N2, PL6N2, tL6N1, CL6N2, RL6N2, iL2N1:

TCGACGACTACTGACCGAAGGCAGCCAGCAAAATCACCA CCATTTGG

tL6N2: TCGACGACTACTGACATGAACGGGTAGAAAATACATACA CAGTATGT

tL6N3: TCGACGACTACTGACCTCATTATGCGCTAATATCAGAGA GTCAGAGG

tL6N4, iL2N2: TCGACGACTACTGACCTTTTGCAATCCTGAATCTTACCA ACCCAGCT

tL6N5, NL6N1, iL2N3: TCGACGACTACTGACGACTTCAACAAGAACGGGTATTAATCTTTCCT

gL6N3, PL6N3, dL6N1, tL6N6, oL6N1, RL6N3, hL6N1, NL6N2:

TCGACGACTACTGACTTCCCAATATTTAGGCAGAGGCATACAACGCCCTTGGGTCGTAGACA

iL2N4: TCGACGACTACTGACTTCCCAATATTTAGGCAGAGGCATACAACGCC

tL6N7, iL2N5: TCGACGACTACTGACCAAAAACACAAATATATTTTAGTT CGCGAGAA

RL6N4: TCGACGACTACTGACCAAAAACACAAATATATTTTAGTTCGCGAGAACTTGGGTCGTAGACA

NL6N3: CAAAAACACAAATATATTTTAGTTCGCGAGAACTTGGGTCGTAGACA

tL6N8, iL2N6: TCGACGACTACTGACAGATCTACCCTTGCTTCTGTAAATATATGTGA

tL6N9, iL2N7: TCGACGACTACTGACATAGGAACAACAGTACCTTTTACACAGATGAA

tL6N10, iL2N8: TCGACGACTACTGACCCAGCTTTACATTATCATTTTGCGTTTAAAAG

gL6N4, dL6N2, tL6N11, CL6N3, oL6N2:

TCGACGACTACTGACTCGACTCTCAAATATCAAACCCTCTCACCTTGCTTGGGTCGTAGACA

iL2N9: TCGACGACTACTGACTCGACTCTCAAATATCAAACCCTC TCACCTTG

gL7N1, PL7N1, CL7N1, RL7N1:

TCGACGACTACTGACATTACCAT ATCACCGGAACCAGAGACCCTCAGAACCGCCACGTTCCAG

gL7N2, PL7N2, CL7N2, RL7N2:

TTATTACGTAAAGGTGGCAACATACCGTCACCGACTTGAGGTAGCACCCTTGGGTCGTAGACA

AL7N1: TCGACGACTACTGACACAAGAATAAGACTCCTTATTACGTAAAGGTGGCAACATACCGTCACC

AL7N2: TCGACGACTACTGACGAGCGTCTGAACACCCTGAACAAAGATAACCCCTTGGGTCGTAGACA

AL7N3: CCGCACTCTTAGTTGCTATTTTGCACGCTAACCTTGGGTCGTAGACA

AL7N4: TCGACGACTACTGACCAGTAATA AATCAATAATCGGCTGACCAAGTA

gL7N3, PL7N3, dL7N1, tL7N1, oL7N1, RL7N3, hL7N1

TCGACGACTACTGACCAGTAATAAATCAATAATCGGCTGACCAAGTACCGCACTCTTAGTTGC

RL7N4: CTTCTGACAGAATCGCCATATTTATTTCGAGCCTTGGGTCGTAGACA

AL7N5, gL7N4, PL7N4, dL7N2, tL7N2, oL7N2, hL7N2:

ACAAAGAAAATTTCATCTTCTGACAGAATCGCCATATTTATTTCGAGCCTTGGGTCGTAGACA

NL7N1: TCGACGACTACTGACTTAATTAAATCGCAAGACAAAGAAAATTTCATCTTCTGACAGAATCGC

RL7N6, NL7N2: TCGACGACTACTGACAACAATAACAGTACATAAATCAATCGTCGCTACTTGGGTCGTAGACA

RL7N7, NL7N3: TTATTAATGAACAAAGAAACCACCTTTTCAGGTTTAACGTTCGGGAGACTTGGGTCGTAGACA

gL7N5, dL7N3, tL7N3, CL7N3, oL7N3:

TCGACGACTACTGACTCTGGTCACCCGAACGTTATTAATGAACAAAGAAACCACCTTTTCAGG

gL7N6, dL7N4, tL7N4, CL7N4, oL7N4:

GCCAACAGATACGTGGCACAGACATGAAAAATCTAAAGCAAATCAATACTTGGGTCGTAGACA

gL8N1, PL8N1, CL8N1, RL8N1:

TGCCTTGACAGTCTCTGAATTTACCCCTCAGAGCCACCACTCTTTTCACTTGGGTCGTAGACA

AL8N1: GCCACCACTCTTTTCATAATCAAATAGCAAGGCCGGAAACTAAAGGTGCTTGGGTCGTAGACA

gL8N2, PL8N2, CL8N2, RL8N2:

TCGACGACTACTGACTAATCAAATAGCAAGGCCGGAAACTAAAGGTGAATTATCATAAAAGAA

AL8N2: TCGACGACTACTGACAATTATCATAAAAGAAACGCAAAGAAGAACTGCTTGGGTCGTAGACA

AL8N3: TCGACGACTACTGACGCATGATTTGAGTTAAGCCCAATAGACGGGAGAATTAACTTTCCAGAG

AL8N4: CCTAATTTAAGCCTTAAATCAAGAATCGAGAACAAGCAAGCGAGCATGCTTGGGTCGTAGACA

AL8N5: TCGACGACTACTGACTAGAAACCAGAGAATATAAAGTACCAGTAGGGCTTGGGTCGTAGACA

gL8N3, PL8N3, dL8N1, tL8N1, oL8N1, RL8N3, hL8N1:

CAAGCAAGCGAGCATGTAGAAACCAGAGAATATAAAGTACCAGTAGGGCTTGGGTCGTAGACA

AL8N6, gL8N4, PL8N4, dL8N2, tL8N2, oL8N2, RL8N4, hL8N2:

TCGACGACTACTGACCTTAATTGCTAAATTTAATGGTTTTGCTGATGCAAATCCATTTTCCCT

RL8N5, NL8N1: TAGAATCCCCTTTTTTAATGGAAACGGATTCGCCTGATTGAAAGAAATCTTGGGTCGTAGACA

RL8N6, NL8N2: TCGACGACTACTGACTGCGTAGAAGAAGGAGCGGAATTACGTATTAACTTGGGTCGTAGACA

gL8N5, dL8N3, tL8N3, CL8N3, oL8N3:

CGGAATTACGTATTAAATCCTTTGGTTGGCAAATCAACAG GAGAGCCACTTGGGTCGTAGACA

RL8N7, NL8N3: TCGACGACTACTGACATCCTTTGGTTGGCAAATCAACAGGAGAGCCAGCAGCAAAATATTTTT

gL8N6, dL8N4, tL8N4, CL8N4, oL8N4:

TCGACGACTACTGACGCAGCAAAATATTTTTGAATGGCTACCAGTAATAAAAGGGCAAACTAT

AL9N1, gL9N1, PL9N1, dL9N1, CL9N1, RL9N1, NL9N1:

TCGACGACTACTGACTGAAACCATTATTAGCGTTTGCCACCTCAGAGCCGCCACCGCCAGAAT

AL9N2, gL9N2, PL9N2, dL9N2, RL9N1, NL9N2:

TCGACGACTACTGACGAATAAGTGACGGAAATTATTCATGTCACCAACTTGGGTCGTAGACA

CL9N2: ATACCCAAACACCACGGAATAAGTGACGGAAATTATTCATGTCACCAACTTGGGTCGTAGACA

AL9N3, gL9N3, PL9N3, dL9N3, RL9N3, NL9N3:

TCGACGACTACTGACAAGAAACAATAACGGAATACCCAAACACCACGCTTGGGTCGTAGACA

AL9N4, gL9N4, PL9N4, dL9N4, RL9N4, NL9N4:

TCGACGACTACTGACCAAAATAAACAGGGAAGCGCATTAATAAGAGCCTTGGGTCGTAGACA

AL9N5, gL9N5, PL9N5, dL9N5, RL9N5, NL9N5:

TCGACGACTACTGACATTTTCATCTTGCGGGAGGTTTTGGCCAGTTACTTGGGTCGTAGACA

AL9N6, gL9N6, PL9N6, dL9N6, RL9N6, NL9N6:

TCGACGACTACTGACGGTAAAGTATCCCATCCTAATTTACCGTTTTTCTTGGGTCGTAGACA

tL9N1: TCGACGACTACTGACGGTAAAGTATCCCATCCTAATTTACCGTTTTTATTTTCATCTTGCGGG

AL9N7, gL9N7, dL9N7, NL9N7:

TCGACGACTACTGACGACCGTGTTAAAGCCAACGCTCAACGACAAAACTTGGGTCGTAGACA

PL9N7, tL9N2: TATGTAAA GAAATACCGACCGTGTTAAAGCCAACGCTCAACGACAAAACTTGGGTCGTAGACA

oL9N1, hL9N1: TCGACGACTACTGACGACCGTGTTAAAGCCAACGCTCAACGACAAAAGGTAAAGTATCCCATC

AL9N8, gL9N8, dL9N8, oL9N2, hL9N2, NL9N8:

TCGACGACTACTGACATAGCGATTATAACTATATGTAAAGAAATACCCTTGGGTCGTAGACA

AL9N9, gL9N9, dL9N9, oL9N3, hL9N3, NL9N9:

TCGACGACTACTGACACCAAGTTAATTTCATTTGAATTATTGAAAACCTTGGGTCGTAGACA

AL9N10, gL9N10, dL9N10, oL9N4, hL9N4, NL9N10:

TCGACGACTACTGACTTCCTGATCACGTAAAACCAAGTTAATTTCATCTTGGGTCGTAGACA

tL9N3, CL9N3, RL9N8:

TCGACGACTACTGACAATTGAGGAAACAATTCGACAACTTCATCATATTCCTGATCACGTAAA

AL9N11, L9N11, dL9N11, oL9N5, hL9N5, NL9N11:

TCGACGACTACTGACAATTGAGGAAACAATTCGACAACTTCATCATACTTGGGTCGTAGACA

AL9N12, gL9N12, dL9N12, tL9N4, CL9N4, oL9N6, RL9N9, hL9N6, NL9N12:

GTCACACGATTAGTCTTTAATGCGGCAACAGTGCCACGCTTTGAAAGGCTTGGGTCGTAGACA

Other sequences can be found in the sequences list for the tall rectangle origami[2].

For single and double lines pcDNA of tall rectangular DNA origami:

L1N1: TCGACGACTACTGACTGAGGACTAGGGAGTTAAAGGCCGCTCCAAAAGGAGCCTTAGCGGAGT

L1N2: TCGACGACTACTGACCCGGAACGTACCAAGCGCGAAACAAGAGGCTTCTTGGGTCGTAGACA

L1N3: TCGACGACTACTGACGCTTGAGATTCATTACCCAAATCATTACTTAGCTTGGGTCGTAGACA

L1N4: TCGACGACTACTGACGTAAGAGCACAGGTAGAAAGATTCTAAATTGGCTTGGGTCGTAGACA

L1N5: TCGACGACTACTGACACCCTGACAATCGTCATAAATATTGAGGCATACTTGGGTCGTAGACA

L1N6: TCGACGACTACTGACCAACATGTTTAGAGAGTACCTTTAAGGTCTTTCTTGGGTCGTAGACA

L1N7: TCGACGACTACTGACCAAGGCAAAGCTATATTTTCATTTCTGTAGCTCTTGGGTCGTAGACA

L1N8: TCGACGACTACTGACATCAATATAACCCTCATATATTTTCATACAGGCTTGGGTCGTAGACA

L1N9: TCGACGACTACTGACTAAACGTTAAAACTAGCATGTCAAAAATCACCCTTGGGTCGTAGACA

L1N10: TCGACGACTACTGACAACCGTGCGAGTAACAACCCGTCGTTAAATTGCTTGGGTCGTAGACA

L1N11: TCGACGACTACTGACCCAGGGTTTTGGGAAGGGCGATCGCGCATCGTCTTGGGTCGTAGACA

L1N12: AGCTGATTACTCACATTAATTGCGTGTTATCCGCTCACAAGGGTAACGCTTGGGTCGTAGACA

L9N1: TCGACGACTACTGACTGAAACCATTATTAGCGTTTGCCACCTCAGAGCCGCCACCGCCAGAAT

L9N2: TCGACGACTACTGACGAATAAGTGACGGAAATTATTCATGTCACCAACTTGGGTCGTAGACA

L9N3: TCGACGACTACTGACAAGAAACAATAACGGAATACCCAAACACCACGCTTGGGTCGTAGACA

L9N4: TCGACGACTACTGACCAAAATAAACAGGGAAGCGCATTAATAAGAGCCTTGGGTCGTAGACA

L9N5: TCGACGACTACTGACATTTTCATCTTGCGGGAGGTTTTGGCCAGTTACTTGGGTCGTAGACA

L9N6: TCGACGACTACTGACGGTAAAGTATCCCATCCTAATTTACCGTTTTTCTTGGGTCGTAGACA

L9N7: TCGACGACTACTGACGACCGTGTTAAAGCCAACGCTCAACGACAAAACTTGGGTCGTAGACA

L9N8: TCGACGACTACTGACATAGCGATTATAACTATATGTAAAGAAATACCCTTGGGTCGTAGACA

L9N9: TCGACGACTACTGACACCAAGTTAATTTCATTTGAATTATTGAAAACCTTGGGTCGTAGACA

L9N10: TCGACGACTACTGACTTCCTGATCACGTAAAACCAAGTTAATTTCATCTTGGGTCGTAGACA

L9N11: TCGACGACTACTGACAATTGAGGAAACAATTCGACAACTTCATCATACTTGGGTCGTAGACA

L9N12: GTCACACGATTAGTCTTTAATGCGGCAACAGTGCCACGCTTTGAAAGGCTTGGGTCGTAGACA

L10N1: AATGCCCCATAAATCCTCATTAAAAGAACCACCACCAGAGTTCGGTCACTTGGGTCGTAGACA

L10N2: TCGACGACTACTGACTAGCCCCCTCGATAGCAGCACCGTAGGGAAGGCTTGGGTCGTAGACA

L10N3: TCGACGACTACTGACTAAATATTTTATTTTGTCACAATCCCGAGGAACTTGGGTCGTAGACA

L10N4: TCGACGACTACTGACACGCAATAATGAAATAGCAATAGCAGAGAATACTTGGGTCGTAGACA

L10N5: TCGACGACTACTGACACATAAAAACAGCCATATTATTTATTAGCGAACTTGGGTCGTAGACA

L10N6: TCGACGACTACTGACCCTCCCGACGTAGGAATCATTACCGAACAAGACTTGGGTCGTAGACA

L10N7: TCGACGACTACTGACAAAATAATAATTCTGTCCAGACGACAAATTCTCTTGGGTCGTAGACA

L10N8: TCGACGACTACTGACTACCAGTAGATAAATAAGGCGTTAGGCTTAGGCTTGGGTCGTAGACA

L10N9: TCGACGACTACTGACTTGGGTTAAGCTTAGATTAAGACGATTAATTACTTGGGTCGTAGACA

L10N10: TCGACGACTACTGACCATTTAACACAAAATCGCGCAGAGATATCAAACTTGGGTCGTAGACA

L10N11: TCGACGACTACTGACATTATTTGTATCAGATGATGGCAAAAGTATTACTTGGGTCGTAGACA

L10N12: TCGACGACTACTGACGACTTTACAAGGTTATCTAAAATAAGTATTAACTTGGGTCGTAGACA

L10N13: TCGACGACTACTGACCACCGCCTCGAACTGATAGCCCTATTATTTACATTGGCAGCAATATTA

Other sequences can be found in the sequences list for the tall rectangle origami[2].

Supplementary references

1. S. Pal, et al. DNA Directed Self-Assembly of Anisotropic Plasmonic Nanostructures. *J. Am. Chem. Soc.* **133**, 17606-17609 (2011).

2. P. W. K. Rothemund. Folding DNA to create nanoscale shapes and patterns. *Nature* **440**, 297-302 (2006).
